# Catalytic Asymmetric Fluorination of Alkyl 1-indanone-2-carboxylates Ruled by Pybox-Eu(III) Combination

**DOI:** 10.3390/molecules24061141

**Published:** 2019-03-22

**Authors:** Albert Granados, Pau Sarró, Adelina Vallribera

**Affiliations:** Department of Chemistry, Universitat Autònoma de Barcelona and Centro de Innovación en Química Avanzada (ORFEO-CINQA), Cerdanyola del Vallès, 08193 Barcelona, Spain; albert.granados@uab.es (A.G.); psarro@iciq.es (P.S.)

**Keywords:** europium, fluorination, enantioselectivity, pybox, keto ester

## Abstract

A highly enantioselective catalytic method for the synthesis of quaternary α-fluoro derivatives of 3-oxo esters is described. The reaction uses europium (III) triflate and commercially available chiral pybox-type C2-symmetric ligand. Excellent results in terms of yields and enantioselectivities were assured using the electrophilic NFSI reagent under mild reaction conditions.

## 1. Introduction

Fluorine has drawn great interest from the scientific community. It has been used for a broad variety of purposes in all fields of chemistry, from the polymer industry, with hydrophobic plastics such as Teflon, to the nuclear power industry, or in the production of refrigerants and new solvents [1,2,3,4]. Of special interest is its use in pharmaceutical chemistry, where about 20% of all pharmaceuticals contain fluorinated moieties (30% in the case of agrochemicals) [5,6,7,8,9,10,11,12,13]. It is well known that the exchange of a hydrogen by a fluorine atom in a therapeutic molecule can produce a dramatic change on its physical and chemical properties, along with its biological activity. Generally, the addition of fluorine containing substituents in a drug enhances its metabolic stability and allows modulating its lipophilicity and bioavailability [12,13]. Thus, due to its importance in the pharmaceutical industry, the enantioselective synthesis of fluorinated molecules [14,15,16] is a great challenge in synthetic organic chemistry, especially under catalytic conditions.

**Scheme 1:**
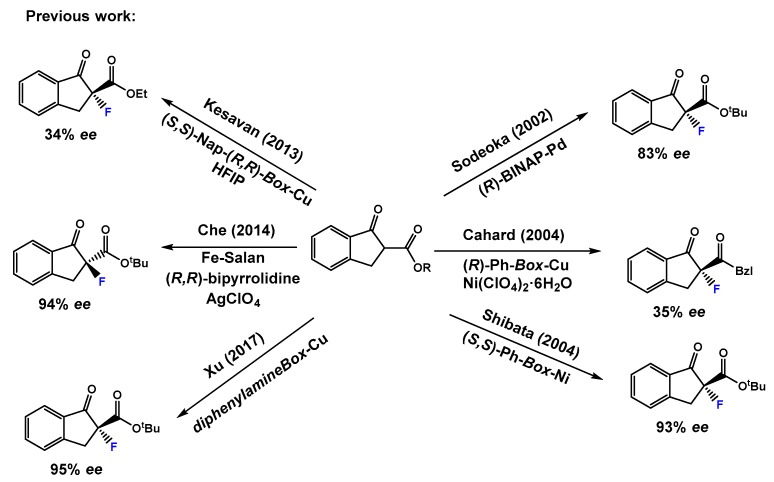
Previous relevant work on enantioselective α-fluorination of alkyl 1-indanone-2-carboxylates using NFSI under metal catalyst.

**Figure 1:**
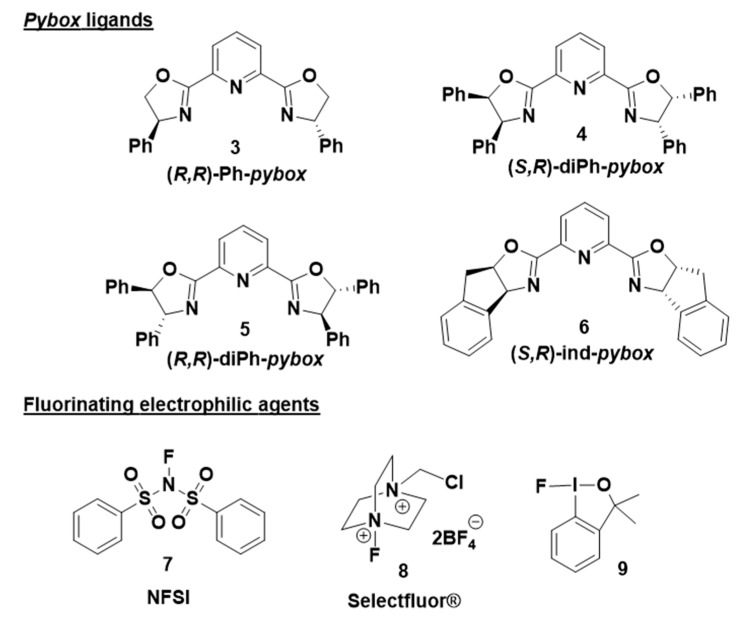
Fluorinating agents and *pybox* C2-symmetry ligands used in the optimization of the electrophilic enantioselective fluorination reaction.

Optically active α-fluorinated cyclic β-keto esters are attractive compounds. On the one hand, they are regarded as non-enolizable and configurationally stable β-keto esters. Moreover, since the ketone is easily converted to other functional groups, they are versatile synthetic precursors. The development of stable sources of electrophilic fluorinating agents (Figure 1) such as *N*-fluorobenzensulfonimide [17] (NFSI), Selectfluor^®^ [18] and 1-fluoro-1,3-dihydro-3,3-dimethyl-1,2-benziodoxole, have had a great impact on catalytic enantioselective electrophilic fluorination. An important breakthrough in this chemistry occurred when Togni and Hintermann [19] described the catalytic α-fluorination reactions of α-substituted acyclic β-keto esters using a taddol-titanium complex and Selectfluor^®^, yielding the fluorinated compounds in 33–90% *ee*. Remarkable contributions of the group of Sodeoka [20,21,22] using palladium-BINAP chiral complexes; Togni [23,24] using Ru(II) chiral complexes; and Cahard [25] using Cu(II) bis(oxazoline) (*Box*) chiral complexes merit also to be mentioned. Much effort has been devoted to the α-fluorination of alkyl 1-indanone-2-carboxylates under metal catalyst (Scheme 1). Sodeoka’s group using a BINAP-Pd(II) complex in the reaction of *tert*-butyl 1-indanone-2-carboxylate and NFSI afforded the α-fluorination product in 83% *ee*. [20] Then, Cahard and collaborators described [25] the reaction of benzyl 1-indanone-2-carboxylate and NFSI under (*R*)-Ph-*Box*-Cu(II) catalysis obtaining 35% *ee* (1 example). In 2004, Shibata’s group performed a study using the combination of Ni(ClO_4_)_2_·6H_2_O and Ph-*Box*-Cu(II) as catalyst and they succeeded with obtaining a 93% *ee* in the reaction of *tert*-butyl 1-indanone-2-carboxylate and NFSI [26]. Moreover, using Nap-*Box*-Cu(II) and NFSI as fluorinating reagent, Kesavan’s group obtained 34% *ee* in the α-fluorination of ethyl 1-indanone-2-carboxylate [27]. The use of iron(III)-salan complexes, NFSI, and AgClO_4_ has also been described, affording a large scope of α-fluorinated alkyl 1-indanone-2-carboxylates in good to excellent *ee* values up to 97%. [28] Moreover, Xu and collaborators [29] have reported a fast and highly enantioselective fluorination of different alkyl 1-indanone-2-carboxylates catalyzed by a chiral non commercial *diphenylamineBox* and Cu(OTf)_2_. The reactions were conducted using a ball mill apparatus [29] (Fritsch Planetary Micro Mill model “Pulverisette 7”) in the absence of solvent and yielding the fluorinated compounds with enantioselectivities up to 99% *ee* (19 examples, 27–99% *ee*). However, the obtained *ee’s* depended on the milling period and rotational speed, and normally in this kind of chemistry, contamination of products can occur as a result of wear and tear principally from the balls, and partially from the casing, which can be a problem, for example, in preparing building blocks for pharmaceuticals. To overcome these disadvantages, and due to our consolidated experience in the enantioselective α-amination of β-keto esters catalyzed by the combination of lanthanides and *pybox* ligands [30,31,32,33], herein we report the results on catalytic enantioselective electrophilic α-fluorination reaction of alkyl 1-indanone-2-carboxylates employing fully commercially available reagents.

## 2. Results and Discussion

**Scheme 2:**
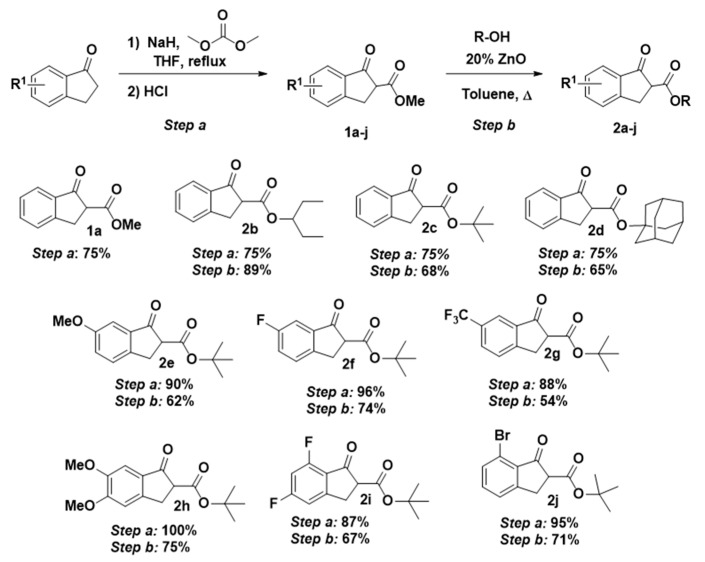
Preparation of alkyl 1-indanone-2-carboxylates **1** and **2.**

In the first part of this present work, we prepared a series of β-keto esters **2** derived from the corresponding 1-indanones. The acylation reaction of 1-indanones was carried out using sodium hydride as a base and dimethylcarbonate as electrophile in THF, obtaining compounds **1a**–**h** and **1j** in excellent yields (Scheme 2, 75–100%). In the case of **1i** (87% yield), methyl 1*H*-imidazole-1-carboxylate was used as acylating reagent; otherwise, exchange of the fluorine atom in 5 position by methoxide was observed [33]. Next, following the reported methodology of our research group [34], we performed the transesterification reaction to successfully afford the secondary and tertiary β-keto esters **2** shown in Scheme 2 (54–89%).

Our previous experience suggested that the use of β-keto esters bearing a bulkier OR group might be useful to achieve enantioinduction, and secondary 3-pentyl group fulfills this requirement. Therefore, we selected **2b** to investigate the effect of different combinations of lanthanides and *pybox* ligands in the α-fluorination process. In the first attempt, we used europium (III) triflate and (*R*,*R*)-Ph-*pybox* (**3**) as catalyst and NFSI (**7**) as the fluorinating agent (Table 1, entry 1). Selection of NFSI was based in previous reported enantioselective fluorination reactions [25,28]. Under these conditions, product **10b** was afforded in a 76% yield but a negligible enantiomeric ratio. Next, we changed the metal source, moving to lanthanum and ytterbium and obtaining in both cases **10b** in nearly racemic form. At this point, we decided to change the chiral ligand. From our previous experience [31,32], we envisioned good results using more sterically hindered *pybox* ligands **4** and **5.** They are non-commercially available and were prepared as previously described [31,35]. The *ee*’s improved but not enough (30–56 % *ee*, entries 4–7 of Table 1). Then, we studied the commercially available (*S,R*)-ind-*pybox,* **6**, and the results improved in a great extend, achieving the highest yield (87%) in combination with La(III) and affording enhanced enantioselective ratios specially in the case of Eu(III) (78% *ee*) (entries 8–10 of Table 1). Selectfluor^®^ (**8**) and 1-fluoro-1,3-dihydro-3,3-dimethyl-1,2-benziodoxole (**9**) were assayed giving worse results in terms of yield and *ee* (entries 11 and 12, Table 1). The hypervalent iodine fluorinated reagent **9** produces the oxidation of the α-position, affording the corresponding α-hydroxylated β-keto ester as the only detected product. This oxidation process has previously been described in the electrophilic cyanation of β-keto esters using 1-cyano-1,3-dihydro-3,3-dimethyl-1,2-benziodoxole [36]. So, we proceed again with the optimized reaction, using NFSI, but now at a low temperature (−30 °C). The reaction of **2b** and **1a** catalyzed by ind-*pybox*-La(III) gave rise to the fluorinating products in good yields (60 and 80% respectively) and moderate *ee’s* (64 and 63% respectively). Additionally, performing the reaction of **2c** (*tert*-butyl ester) in the same conditions, product **10c** was obtained in an enhanced 76% *ee* (entry 15, Table 1). Finally, changing to ind-*pybox*-Eu(III) compound **10c** was achieved in an excellent 96% *ee* (entry 16, Table 1).

**Figure 2:**
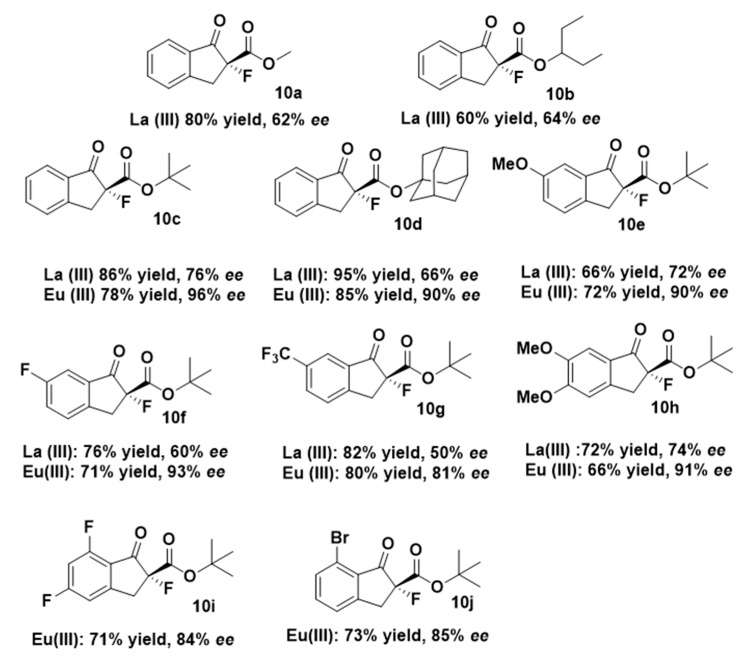
Scope of the reaction of alkyl 1-indanone-2-carboxylates **2** with different groups in aromatic positions. All the reactions were carried out at the same conditions (−30 °C), see experimental part.

Therefore, as the best performance in terms of selectivity was obtained using sterically hindered *tert*-butyl 1-oxo-indanecarboxylate **2c** (entry 16, Table 1), we also performed the reaction with adamantan-1-yl 1-oxo-2-indanecarboxylate in the same conditions, obtaining **10d** in a slightly lower 90% *ee* (Figure 2). Thus, we focused our attention on other *tert*-butyl 1-indanone-2-carboxylates possessing different groups in the aromatic ring. α-Fluorinated compounds **10e**–**j** were achieved in high yields (66–85%) and enantiomeric excesses up to 93% when using *ind*-pybox-Eu(III) combination. Lanthanium gave lower selectivities in all the studied substrates (Figure 2). First, we selected three different substituents in 6 positions of the aromatic ring. The change from OMe (**10e**, 90% *ee*) to CF_3_ (**10g**, 81% *ee*) lessen the *ee* and the reaction time from 24h to 8h. Addition of an extra OMe group in 5 position (**10h**) did not improve the *ee* (91%). Compounds **10i** and **10j** were obtained in 84 and 85% *ee* respectively. In general, the presence of electron withdrawing groups harmed the *ee* (**10g**, **10i** and **10j**, Figure 2), except in the case of **10f** (F in 6 position), which was obtained in an excellent 93% *ee*.

The assignment of the absolute configuration of compounds **10** was done by comparison of the sign of the optical rotation values already described for **10c**–**f** [28]. For new compounds **10b** and **10g–j** the assignment was based on the comparison of the circular dichroism (CD) (all studied compounds show a negative Cotton Effect, see supplementary information). In all cases, the absolute configuration was assigned to be *R*. The advantage of this method is that we could also have access to **(*S)*-10** enantiomers by using commercially available (*R,S*)-ind-*pybox.*

**Scheme 3:**
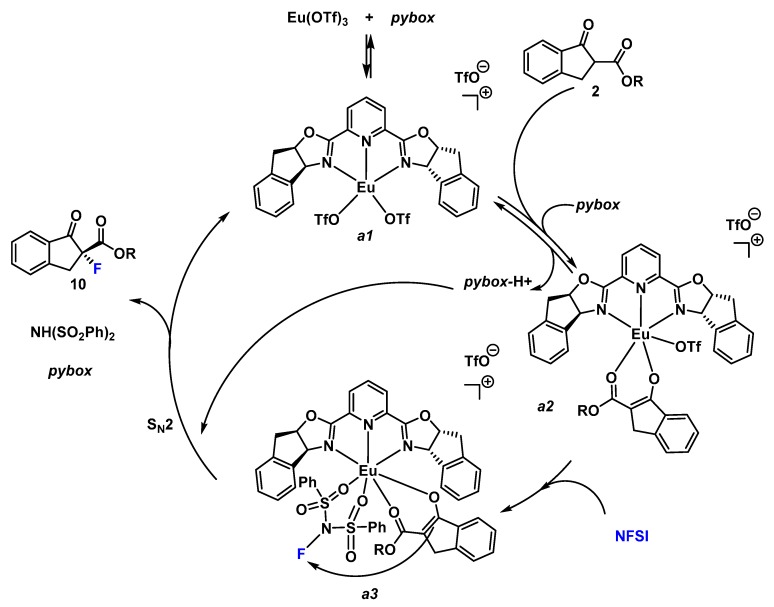
Plausible catalytic cycle.

Furthermore, a plausible catalytic cycle is proposed for this enantioselective fluorination reaction (Scheme 3) [30,33]. First, we propose the formation of the [*pybox*Eu(OTf)_2_]^+^ (**a1**) complex that can exchange the labile triflate ligand by the enolate form of the β-keto ester affording the complex [*pybox*Eu(OTf)(**enolate-2**]^+^ (**a2**). Similar complexes were detected by ESI Mass Spectometry when mixing La(OTf)_3_ and *ip*-pybox [30]. The pybox ligand is the responsible base for the formation of the **enolate-2**. Abstraction of the intercarbonylic proton can be produced directly from **2** (as indicated in Scheme 3); or once the β-keto ester is coordinated to the europium enhancing its acidity. Next, a coordination of the NFSI, through the donor oxygen atom, to the europium forming a ternary complex is proposed. The known large coordination number of lanthanides makes that possible. The enantioselectivity of the reaction stems from the efficient blockage of one of the diastereotopic faces of the Eu(III) enolate by one unit of the C2-symmetric ligand. Subsequent reaction affords the formation of the stereogenic C–F bond through a S_N_2 mechanism.

## 3. Materials and Methods

**General transesterification procedure:** In a round-bottomed flask, the corresponding 1-indanone (1 eq.), ZnO (0.2 eq.) and the corresponding alcohol (10 equivalents) were dissolved in toluene (5 mL/mol 1-indanone). The reaction mixture was heated up to 140 °C under a distillation setup until total conversion of the 1-indanone was observed by TLC (from 6 to 8 h). If necessary, more toluene was added. Afterwards, the reaction mixture was filtered through Celite^®^ and the solvent was removed under reduced pressure. The product obtained was purified by column chromatography on silica-gel.

*Pentan-3-yl 1-oxo-2,3-dihydro-1H-indene-2-carboxylate* (**2b**): Following the general procedure, 1.00 g (5.26 mmol, 1 eq) of **1a** was allowed to react with 3-pentanol (52.7 mmol, 10 eq.) and ZnO (0.09 g, 1.06 mmol, 0.2 eq.) in 6 mL of toluene for 5 h. After purification by column chromatography on silica-.gel (hexanes (95): AcOEt (5)) a brown oil was obtained in 90% yield (1.17 g). IR (ATR) *ν* = 2968, 1711 (C=O), 1608, 1572, 1207, 1092 cm^−1^; ^1^H-NMR (360 MHz [D]CDCl_3_, 298 K, TMS) δ = 10.50 (bs, 0.08H, O*H*, enol), 7.79 (d, ^3^*J_(H,H)_* = 7.5 Hz, 1H, Ar*H*), 7.64 (t, ^3^*J_(H,H)_* = 7.1 Hz, 1H, Ar*H*), 7.52 (d, ^3^*J_(H,H)_* = 7.5 Hz, 1H, Ar*H*), 7.50–7.44 (m, 0.17H, Ar*H*, enol), 7.41 (^3^*J_(H,H)_*, *J* = 7.5 Hz, 1H, Ar*H*), 4.98 (quint, ^3^*J_(H,H)_* = 6.1 Hz, 0.16H, OC*H*, enol), 4.86 (quint, ^3^*J_(H,H)_* = 6.3 Hz, 1H, OC*H*,), 3.74 (dd, ^3^*J_(H,H)_* = 4.2 Hz, ^3^*J_(H,H)_* = 8.7 Hz, 1H, CH_2_C*H*), 3.59 (d, ^3^*J_(H,H)_* = 4.2 Hz, 0.42H, C*H*_2_C, enol), 3.55 (d, ^3^*J_(H,H)_* = 3.7 Hz, 1H, C*H*_2_CH), 3.40 (dd, ^3^*J_(H,H)_* = 8.3 Hz, *J* = 17.5 Hz, 1H, C*H*_2_CH), 1.70 (m, 1H, C*H*_2_CH_3_, enol), 1.65 (m, 4H, C*H*_2_CH_3_), 1.02–0.86 ppm (m, 7.36, CH_2_C*H*_3_); ^13^C-NMR (101 MHz, [D]CDCl_3_, 298 K, TMS) δ = 199.6, 169.0, 153.6, 143.1, 137.0, 135.3, 135.2, 129.1, 127.7, 126.7, 126.5, 124.6, 124.6, 120.6, 102.7, 78.3, 76.4, 53.6, 32.52, 30.3, 26.6, 26.4, 9.6, 9.4 ppm; HRMS (ESI): (M + Na)^+^ Calcd for C_15_H_18_O_3_Na 269.1148; found 269.1144.

*Tert-butyl-5,7-difluoro-1-oxo-2,3-dihydro-1H-indene-2-carboxylate* (**2i**): Following the general procedure, 0.50 g (2.21 mmol, 1 eq) of **1i** was allowed to react with *tert*-butanol (22.1 mmol, 10 eq.) and ZnO (0.04 g, 0.45 mmol, 0.2 eq.) in 5 mL of toluene for 5 h. After purification by column chromatography on silica-.gel (hexanes (95): AcOEt (5)) a purple oil was obtained in 64% yield (0.38 g). IR (ATR) *ν* = 2961, 1758 (C=O), 1722, 1617, 1283, 1045 cm^−1^; ^1^H-NMR (360 MHz [D]CDCl_3_, 298 K, TMS) δ = 10.62 (bs, 0.07H, O*H*, enol), 6.97 (d, ^3^*J_(H,F)_* = 7.3 Hz, 1H, Ar*H*), 6.74 (t, ^3^*J_(H,F)_* = 9.1 Hz, 1H, Ar*H*), 3.64 (dd, ^3^*J_(H,H)_* = 3.9 Hz, ^3^*J_(H,H)_* = 8.2 Hz, 1H, CH_2_C*H*), 3.48 (dd, ^3^*J_(H,H)_* = 4.4 Hz, ^3^*J_(H,H)_* = 16.7 Hz, C*H*_2_CH), 3.30 (dd, ^3^*J_(H,H)_* = 8.3 Hz, *J* = 17.6 Hz, 1H, C*H*_2_CH), 1.47 (s, 9H, C(C*H*_3_)_3_); ^19^F-NMR (235 MHz [D]CDCl_3_, 298 K, TMS) δ = −99.9 (d, ^3^*J_(F,F)_* = 13.7 Hz, 1F), −111.3 ppm (d, ^3^*J_(F,F)_* = 13.7 Hz, 1F); ^13^C-NMR (91 MHz, [D]CDCl_3_, 298 K, TMS) δ = 194.5 (d, ^3^*J_(C,F)_* = 2.2 Hz), 167.8 (dd, ^3^*J_(C,F)_* = 3.9 Hz, ^1^*J_(C,F)_* = 259.5 Hz), 167.5 (s), 160.0 (dd, ^3^*J_(C,F)_* = 14.2 Hz, ^1^*J_(C,F)_* = 267.5 Hz), 157.7 (m), 120.1 (m), 109.5 (dd,^4^*J_(C,F)_* = 4.2 Hz, ^2^*J_(C,F)_* = 22.5 Hz), 103.9 (dd, ^2^*J_(C,F)_* = 22.7 Hz, ^2^*J_(C,F)_* = 24.7 Hz), 82.5 (s), 55.0 (s), 27.8 (s) ppm; HRMS (ESI): (M + Na)^+^ Calcd for C_14_H_14_F_2_O_3_Na 291.0803; found 291.0805.

**General procedure for the enantioselective electrophilic fluorinations:** In a 10 mL Schlenk flask in presence of 4 Å MS, Ln(OTf)_3_ (0.13 eq.) and the desired *pybox* ligand (0.15 eq.) were dissolved with dry ACN (5 mL). The colorless reaction mixture was left stirring at room temperature under argon atmosphere overnight. Next, the corresponding indanone derivative **2** (80 mg; 1 eq.) was added to the reaction mixture and left stirring at room temperature for 1 h. Then, the reaction mixture was cooled down until −30 °C and, once at this temperature, NFSI (1.15 eq.) was added to the mixture in one portion. The reaction mixture was left at this temperature under argon atmosphere until complete conversion of the reagent. Afterwards, the solvent was removed under reduced pressure and the product was purified by column chromatography on silica gel, yielding the α-fluorinated compound.

*Pentan-3-yl (R)-2-fluoro-1-oxo-2,3-dihydro-1H-indene-2-carboxylate* (**10b**)*:* Following the general procedure using La(OTf)_3_ and *(S,R)*-ind-*pybox,* 79 mg (0.29 mmol, 60% yield) of **10b** were obtained as a tan oil from 0.37 mmol of **2b**. It was purified by column chromatography on silica-gel (hexane/AcOEt 5:1). IR (ATR) *ν* = 2970, 1760 (C=O), 1607 (C=O), 1464, 1287, 1214, 1191, 1156, 1100 cm^−1^; ^1^H-NMR (250 MHz [D]CDCl_3_, 298 K, TMS) δ = 7.85 (d, ^3^*J_(H,H)_* = 8.1 Hz, 1H, Ar*H*), 7.71 (t, ^3^*J_(H,H)_* = 7.4 Hz, 1H, Ar*H*), 7.57 (m, 2H, Ar*H*), 4.90 (quint, ^3^*J_(H,H)_* = 6.3 Hz, 1H, OC*H*), 3.78 (dd, ^2^*J_(H,H)_* = 11.4 Hz, ^3^*J_(H,F)_* = 17.8 Hz, 1H, C*H*_2_CF), 3.40 (dd, ^2^*J_(H,H)_* = 22.8 Hz, ^3^*J_(H,F)_* = 25.7 Hz, 1H, C*H*_2_CF), 1.55 (m, 4H, C*H*_2_CH_3_), 0.84 (m, 6H); ^13^C-NMR (91 MHz [D]CDCl_3_, 298 K, TMS) δ = 195.5 (d, ^2^*J_(C,F)_* = 18.2 Hz), 167.1 (d, ^2^*J_(C,F)_* = 27.8 Hz), 150.9 (d, ^3^*J_(H,F)_* = 3.7 Hz), 136.4 (d, *J* = 3.0 Hz, Ar-C), 133.4 (s), 128.6 (s), 126.5 (d, ^4^*J_(C,F)_* = 1.2 Hz), 125.5, 94.5 (d, ^1^*J_(C,F)_* = 201.2 Hz), 79.6 (s), 38.4 (d, ^2^*J_(C,F)_* = 24.1 Hz), 26.4 (s), 26.3 (s), 9.5 (s), 9.3 (s). ^19^F-NMR (235 MHz [D]CDCl_3_, 298 K, TMS) δ = −164.8 ppm (s, 1F); HPLC: Daicel Chiralpack AD-H, Hexane/*^i^*PrOH=99.5:0.5, 0.75 mL/min, 254 nm, t*_r_*(minor) = 33.4 min, t*_r_*(major) = 40.7 min (64% *ee*); [α]_20_^D^: −10.3 (c=5.9, CHCl_3_). HRMS (ESI): (M + Na)^+^ Calcd for C_15_H_17_O_3_FNa 287.1054; found 287.1059.

*Tert-butyl (R)-2-fluoro-1-oxo-6-(trifluoromethyl)-2,3-dihydro-1H-indene-2-carboxylate* (**10g**): Following the general procedure, using Eu(OTf)_3_ and (*S,R*)-ind-*pybox*, 67 mg (0.21 mmol, 80% yield) of **10g** were obtained as a brown oil from 0.31 mmol of the starting material. It was purified by column chromatography on silica-gel (hexane/AcOEt 7:3). IR (ATR) *ν* = 2916, 2849, 1736 (C=O), 1467, 1330, 1259, 1179 cm^−1^; ^1^H-NMR (360 MHz [D]CDCl_3_, 298 K, TMS) δ = 8.11 (s, 1H, Ar*H*), 7.95 (d, ^3^*J_(H,H)_* = 7.9 Hz, 1H, Ar*H*), 7.68 (d, ^3^*J_(H,H)_* = 7.9 Hz, 1H, Ar*H*), 3.82 (dd, ^2^*J_(H,H)_* = 10.7 Hz, ^3^*J_(H,F)_* = 18.5 Hz, 1H, C*H*_2_CF), 3.47 (dd, ^2^*J_(H,H)_* = 18.2 Hz, ^3^*J_(H,F)_* = 21.8 Hz, 1H, C*H*_2_CF), 1.45 (s, 9H, OC(C*H*_3_)_3_); ^19^F-NMR (235 MHz [D]CDCl_3_, 298 K, TMS) δ = −63.2 (s, 3F, C*F*_3_), -163.9 pm (s, 1F, C*F*); ^13^C-NMR (91 MHz [D]CDCl_3_, 298 K, TMS) δ = 194.7 (d, ^2^*J_(C,F)_* = 18.6 Hz), 165.6 (d, ^2^*J_(C,F)_* = 27.5 Hz), 154.7 (d, ^3^*J_(C,F)_* = 3.6 Hz), 133.9 (s), 132.8 (q, ^4^*J_(C,F)_* = 3.2 Hz), 131.4 (q, ^2^*J_(C,F)_* = 33.5 Hz), 127.4 (s), 123.4 (q, ^1^*J_(C,F)_* = 272.5 Hz), 122.5 (q, ^3^*J_(C,F)_* = 4.0 Hz), 94.2 (d, ^1^*J_(C,F)_* = 203.5 Hz), 84.7 (s), 38.3 (d, ^2^*J_(C,F)_* = 24.2 Hz), 27.2 (s) ppm; HPLC: Daicel Chiralpack AD-H, Hexane/*^i^*PrOH=99.9:0.1 0.7 mL/min, 254 nm, t*_R_*(minor) = 20.2 min, t*_S_*(major) = 23.1 min (81% *ee*); [α]_20_^D^: −5.6 (c=6.2, CHCl_3_). HRMS (ESI): (M + Na)^+^ Calcd for C_15_H_14_O_3_F_4_Na 341.0771; found 341.0772.

*Tert-butyl (R)-2,5,7-trifluoro-1-oxo-2,3-dihydro-1H-indene-2-carboxylate* (**10i**): Following the general procedure, using Eu(OTf)_3_ and (*S,R*)-ind-*pybox*, 45 mg (0.15 mmol, 71% yield) of **10i** were obtained as a colorless oil from 0.22 mmol of **2i**. It was purified by column chromatography on silica-gel (hexane/AcOEt 9:1). IR (ATR) *ν* = 2921, 1751 (C=O), 1721, 1484, 1298, 1149,1040 cm^−1^; ^1^H-NMR (400 MHz [D]CDCl_3_, 298 K, TMS) δ = 7.01 (d, ^3^*J_(H,F)_* = 6.5 Hz, 1H, Ar*H*), 6.86 (t, ^3^*J_(H,F)_* = 8.2 Hz, 1H, Ar*H*), 3.74 (dd, ^2^*J_(H,H)_* = 10.0, ^3^*J_(H,F)_* = 17.6 Hz, 1H), 3.42 (dd, ^2^*J_(H,H)_* = 15.5, ^3^*J_(H,F)_* = 23.9 Hz, 1H), 1.48 ppm (s, 9H, OC(C*H*_3_)_3_); ^19^F-NMR (376 MHz [D]CDCl_3_, 298 K, TMS) δ = −94.9 (d, ^4^*J_(F,F)_* = 14.7 Hz, 1F, Ar*F*), −106.4 (d, ^4^*J_(F,F)_* = 14.7 Hz, 1F, Ar*F*), −162.5 ppm (s, 1F, C*F*); ^13^C-NMR (101 MHz [D]CDCl_3_, 298 K, TMS) δ = 193.2 (d, ^2^*J_(C,F)_* = 18.2 Hz), 167.8 (dd, ^3^*J_(C,F)_* = 3.9 Hz, ^1^*J_(C,F)_* = 259.5 Hz), 165.8 (d, ^2^*J_(C,F)_* = 24.5 Hz), 160.0 (dd, ^3^*J_(C,F)_* = 14.2 Hz, ^1^*J_(C,F)_* = 267.5 Hz), 153.4 (m), 120.1, 109.8 (dd,^4^*J_(C,F)_* = 4.2 Hz, ^2^*J_(C,F)_* = 22.5 Hz), 95.4 (d, ^1^*J_(C,F)_* = 202.4 Hz), 82.5 (s), 38.9 (d, ^2^*J_(C,F)_* = 24.1 Hz), 27.8 (s) ppm; HPLC: Daicel Chiralpack AD-H, Hexane/*^i^*PrOH=97:3, 1.0 mL/min, 254 nm, t*_r_*(major) = 8.7 min, t*_r_*(minor) = 12.9 min (84% *ee*); [α]_20_^D^: −10.7 (c=6.8, CHCl_3_). HRMS (ESI): (M + Na)^+^ Calcd for C_14_H_13_F_3_O_3_Na 309.0709; found 309.0709.

*Tert-butyl (R)-2-fluoro-7-bromo-1-oxo-2,3-dihydro-1H-indene-2-carboxylate* (**10j**): Following the general procedure, using Eu(OTf)_3_ and (*S,R*)-ind-*pybox*, 48 mg (0.14 mmol, 73% yield) of **10j** were obtained as a colorless oil from 0.20 mmol of **2j**. It was purified by column chromatography on silica-gel (hexane/AcOEt 9:1). ^1^H-NMR (360 MHz [D]CDCl_3_, 298 K, TMS) δ = 7.61 (d, ^3^*J_(H,H)_* = 7.6 Hz, 1H, Ar*H*), 7.51 (t, ^3^*J_(H,H)_* = 7.6 Hz, 1H, Ar*H*), 7.45 (d, ^3^*J_(H,H)_* = 7.6 Hz, 1H, Ar*H*), 3.69 (dd, ^2^*J_(H,H)_* = 11.2, ^3^*J_(H,F)_* = 17.6 Hz, 1H), 3.37 (dd, ^2^*J_(H,H)_* = 17.6, ^3^*J_(H,F)_* = 22.9 Hz, 1H), 1.44 ppm (s, 9H, OC(C*H*_3_)_3_); ^19^F-NMR (235 MHz [D]CDCl_3_, 298 K, TMS) δ = −163.8 ppm (s, 1F); ^13^C-NMR (91 MHz [D]CDCl_3_, 298 K, TMS) δ = 193.2 (d, ^2^*J_(C,F)_* = 19.2 Hz), 165.8 (d, ^2^*J_(C,F)_* = 27.6 Hz), 153.4 (d, ^3^*J_(C,F)_* = 3.6 Hz), 136.7 (s), 133.4 (s), 131.3 (s), 125.4 (s), 121.2 (s), 94.5 (d, ^1^*J_(C,F)_* = 202.4 Hz), 84.5 (s), 37.4 (d, ^2^*J_(C,F)_* = 24.1 Hz), 27.8 (s) ppm; HPLC: Daicel Chiralpack AD-H, Hexane/*^i^*PrOH=97:3, 1.0 mL/min, 254 nm, t*_r_*(major) = 12.1 min, t*_r_*(minor) = 18.9 min (85% *ee*); [α]_20_^D^: −34.7 (c=6.3, CHCl_3_). HRMS (ESI): (M + Na)^+^ Calcd for C_14_H_14_BrFO_3_Na 351.0003; found 351.0005.

## 4. Conclusions

In conclusion, we have established a catalytic method for the highly enantioselective α-fluorination of a series of alkyl 1-oxo-indanecarboxylates, using europium (III) triflate and (*S,R*)-ind-*pybox* as pre-catalyst, and NFSI as electrophilic fluorinating agent in acetonitrile at −30 °C. Selectivities up to 96% *ee* could be obtained in mild conditions, and using a commercially available chiral ligand. Results reveal a dependence of the enantiocontrol on the sterically hindrance of the ester in substrates. Access to both enantiomers of the α-fluorinated oxo ester is guaranteed by the commercial availability of both (*R,S*) and (*S,R*) ind-*pybox* C2-symmetric ligands.

## Figures and Tables

**Table 1:**
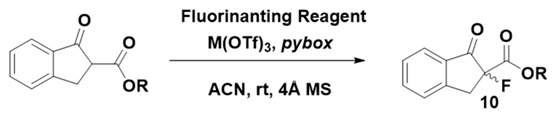
Optimization of the enantioselective α-fluorination reaction.

Entry	2	Metal	Ligand	Fluorinating Reagent	Yield (%)	*ee* (%)[a]
1	**2b**	Eu^+3^	**3**	NFSI	76	2
2	**2b**	La^+3^	**3**	NFSI	74	5
3	**2b**	Yb^+3^	**3**	NFSI	68	2
4	**2b**	La^+3^	**4**	NFSI	81	56
5	**2b**	Eu^+3^	**4**	NFSI	45	30
6	**2b**	Yb^+3^	**4**	NFSI	78	54
7	**2b**	La^+3^	**5**	NFSI	85	50
8	**2b**	La^+3^	**6**	NFSI	87	60
9	**2b**	Eu^+3^	**6**	NFSI	72	78
10	**2b**	Yb^+3^	**6**	NFSI	70	60
11	**2b**	La^+3^	**6**	**8**	59	14
12	**2b**	La^+3^	**6**	**9**	0	*n.d.*
13[b]	**1b**	La^+3^	**6**	NFSI	60	64
14[b]	**1a**	La^+3^	**6**	NFSI	80	63
15[b]	**2c**	La^+3^	**6**	NFSI	86	76
16[b]	**2c**	Eu^+3^	**6**	NFSI	78	96

[a] The racemic compounds **10** were prepared by reaction of the alkyl 1-indanone-2-carboxylates with Selectfluor^®^ (1 equiv.) in CH_3_CN at 50 °C. The *ee*’s were determined by using chiral HPLC (see supplementary information). [b] Reaction done at −30 °C.

## Supplementary Materials

The following are available online: synthesis and characterization of starting materials **1a-j** and **2a-j**, and pybox ligands **4** and **5**. Spectra, HPLC and CD of compounds **10a-j**.
