# Supplementary material for: Catalytic Asymmetric Fluorination of Alkyl 1-indanone-2-carboxylates Ruled by Pybox-Eu(III) Combination
Source: Molecules. 2019 Mar 22;24(6):1141. doi: 10.3390/molecules24061141 (PMC6471818; doi:10.3390/molecules24061141)

Supporting Information

Catalytic asymmetric fluorination of alkyl 1-indanone-2-carboxylates ruled by *pybox*-Eu(III) combination

Albert Granados,[†] Pau Sarró,[†] Adelina Vallribera[†,*]

†Department of Chemistry, Centro de Innovación en Química Avanzada (ORFEO-CINQA). Universitat Autònoma de Barcelona, 08193, Cerdanyola del Vallès, Barcelona (Spain).

Table of Contents

1. **General Information……………………………………………… S2**
2. **Starting Materials ………………………………………………..... S2**
3. **General procedure for the acylation reactions …………………. S2**
4. **Data of compounds 10…………………………………………… S3**
5. **Synthesis of pybox ligands 4 and 5 ……………………………… S5**
6. **Spectra, HPLC and CD of compounds…………………………… S6**

1. General Information

GLC chromatography was performed on a capillary column (5% biphenyl and 95% dimethylpolysiloxane) of 15 m × 0.25 mm with stationary phase diameter of 0.25 µm. Column chromatography was performed on silica gel (230−400 mesh). IR spectra were determined either by transmission or by attenuated total reflectance mode (ATR). Enantiomeric excesses were determined, unless otherwise stated, by HPLC using a chiral column Chiracel Daicel-AD-H. NMR spectra were recorded operating at 250, 360, and 400 MHz. Optical rotations are reported as follows: $[\alpha]_D^{rt}$ (c in g per 100 mL, solvent). ^{13}C NMR spectra were registered at 63, 91, and 101 MHz. Elemental analyses are the average of two determinations. HRMS were recorded by a Bruker micrOTOF-QII mass spectrometer (fly time analyzer) through positive electrospray ionization.

2. Starting materials

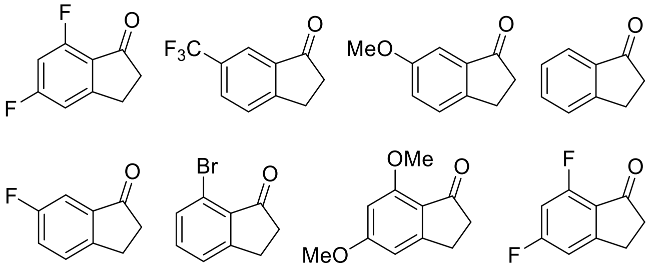

Purchased from
comercial sources

3. General procedure for the acylation reactions

In a round-bottomed flask NaH 60% in grease (3 eq.) was placed. Then, it was dissolved in dry THF (0.2 mL/mmol NaH) while stirring under argon atmosphere, forming a white suspension. Next, dimethyl carbonate (3 eq.) was added (methyl 1*H*-imidazole-1-carboxylate in case **1i**) to the suspension and the corresponding ketone (1 eq.) was dissolved with dry THF (1 mL/mol ketone) in an addition funnel. The ketone solution was added to the reaction mixture dropwise in a period of 3-5 minutes. The reaction mixture was heated to reflux until total conversion of the reagent was observed by TLC (3-5 h). Afterwards, 1 M HCl was added to the mixture until pH = 2-3. The aqueous mixture was then extracted with dichloromethane. The organic fraction was dried over anhydrous Na_2SO_4 and the solvent was removed under reduced pressure.

Finally, the product was purified by column chromatography on silica-gel when necessary.

4. **Data of compounds 10 (already described) obtained following the general procedure of enantioselective α-fluorination described in the experimental part of the manuscript:**

Methyl (R)-2-fluoro-1-oxo-2,3-dihydro-1H-indene-2-carboxylate (**10a**):[12] Following the general procedure, 70 mg (80% yield) of **10a** were obtained as a white oily solid from 0.42 mmol of **1a** using La(OTf)$_3$ and (*S*,*R*)-ind-*pybox*. It was purified by column chromatography on silica-gel (hexane/AcOEt 4:1). ^{1}H NMR (250 MHz [D]CDCl$_3$, 298 K, TMS) δ = 7.83 (d, *J* = 7.9 Hz, 1H, Ar*H*), 7.71 (t, *J* = 7.9 Hz, 1H, Ar*H*), 7.49 (m, 2H, Ar*H*), 3.80 (m, 4H, C*H*$_2$CF and OC*H*$_3$), 3.44 ppm (dd, *J* = 23.4 Hz, *J* = 23.6 Hz, 1H, C*H*$_2$CF); ^{19}F NMR (235 MHz [D]CDCl$_3$, 298 K, TMS) δ = -160.9 ppm (s, 1F); HPLC: Daicel Chiralpack AD-H, Hexane/^{i}PrOH=99.5:0.5, 0.8 mL/min, 254 nm, t_r(minor) = 38.7 min, t_r(major) = 40.7 min (62% *ee*); $[\alpha]_{20}^{D}$: -8.6 (c=5.9 , CHCl$_3$).

Tert-butyl (R)-2-fluoro-1-oxo-2,3-dihydro-1H-indene-2-carboxylate (**10c**):[12] Following the general procedure, using Eu(OTf)$_3$ and (*S*,*R*)-ind-*pybox*, 67 mg (78% yield) of **10c** were obtained as a colorless oil from 0.34 mmol of **2c**. It was purified by column chromatography on silica-gel (hexane/AcOEt 9:1). ^{1}H NMR (250 MHz [D]CDCl$_3$, 298 K, TMS) δ = 7.84 (d, $^3J_{(H,H)}$ = 7.6 Hz, 1H, Ar*H*), 7.70 (t, $^3J_{(H,H)}$ = 7.6 Hz, 1H, Ar*H*), 7.47 (m, 2H, Ar*H*), 3.74 (dd, $^3J_{(H,H)}$ = 10.7 Hz, $^2J_{(H,F)}$ = 18.3 Hz, 1H, C*H*$_2$CF), 3.40 (dd, $^2J_{(H,H)}$ = 22.6 Hz, $^3J_{(H,F)}$ = 24.9 Hz, 1H, C*H*$_2$CF), 1.44 (s, 9H, OC(C*H*$_3$)$_3$); ^{19}F NMR (235 MHz [D]CDCl$_3$, 298 K, TMS) δ = -164.4 ppm (s, 1F); HPLC: Daicel Chiralpack AD-H, Hexane/^{i}PrOH=99:1 1.0 mL/min, 254 nm, t_r(minor) = 24.6 min, t_r(major) = 33.7 min (96% *ee*); $[\alpha]_{20}^{D}$: 11.2 (c=5.7 , CHCl$_3$).

Adamantan-1-yl (R)-2-fluoro-1-oxo-2,3-dihydro-1H-indene-2-carboxylate (**10d**):[12] Following the general procedure, using Eu(OTf)$_3$ and (*S*,*R*)-ind-*pybox*, 74 mg (85% yield) of **10d** were obtained as a yellowish oil from 0.26 mmol of **2d**. It was purified by column chromatography on silica-gel (hexane/AcOEt 4:1). ^{1}H NMR (250 MHz [D]CDCl$_3$, 298 K, TMS) δ = 7.82 (d, $^3J_{(H,H)}$ = 7.8 Hz, 1H, Ar*H*), 7.70 (t, $^3J_{(H,H)}$ = 7.8 Hz, 1H, Ar*H*), 7.46 (m, 2H, Ar*H*), 3.73 (dd, $^2J_{(H,H)}$ = 10.5 Hz, $^3J_{(H,F)}$ = 18.1 Hz, 1H, C*H*$_2$CF), 3.39 (dd, $^2J_{(H,H)}$ = 22.7 Hz, $^3J_{(H,F)}$ = 23.8 Hz, 1H, C*H*$_2$CF), 2.08 (m, 9H,

O*Ada*), 1.63 ppm (s, 6H, O*Ada*); ^{19}F NMR (235 MHz [D]$CDCl_3$, 298 K, TMS) δ = -164.5 ppm (s, 1F); HPLC: Daicel Chiralpack AD-H, Hexane/^{i}PrOH=99:1, 1.0 mL/min, 254 nm, t_r(minor) = 23.5 min, t_r(major) = 31.4 min (90% *ee*); $[\alpha]_{20}^{D}$: 6.9 (c=5.7 , $CHCl_3$).

Tert-butyl (R)-2-fluoro-6-methoxy-1-oxo-2,3-dihydro-1H-indene-2-carbox-ylate (**10e**):[12] Following the general procedure, using $Eu(OTf)_3$ and (*S*,*R*)-ind-*pybox*, 61 mg (72% yield) of **10e** were obtained as a yellowish oil from 0.33 mmol of **2c**. It was purified by column chromatography on silica-gel (hexane/AcOEt 9:1). ^{1}H NMR (250 MHz [D]$CDCl_3$, 298 K, TMS) δ = 7.38 (d, $^3J_{(H,H)}$ = 8.0 Hz, 1H, Ar*H*), 7.24 (m, 2H, Ar*H*), 3.85 (s, 3H, OCH_3), 3.65 (dd, $^2J_{(H,H)}$ = 10.5, $^3J_{(H,F)}$ = 17.6 Hz, 1H, CH_2CF), 3.31 (dd, $^2J_{(H,H)}$ = 22.7, $^3J_{(H,F)}$ = 24.2 Hz, 1H, CH_2CF), 1.44 ppm (s, 9H, OC(CH_3)$_3$); ^{19}F NMR (235 MHz [D]$CDCl_3$, 298 K, TMS) δ = -163.9 ppm (s, 1F); HPLC: Daicel Chiralpack AD-H, Hexane/^{i}PrOH=99:1, 1.0 mL/min, 254 nm, t_r(minor) = 18.1 min, t_r(major) = 20.0 min (90% *ee*); $[\alpha]_{20}^{D}$: 10.9 (c=5.8 , $CHCl_3$).

Tert-butyl (R)-2,6-difluoro-1-oxo-2,3-dihydro-1H-indene-2-carboxylate (**10f**):[12] Following the general procedure, using $Eu(OTf)_3$ and (*S*,*R*)-ind-*pybox*, 61 mg (71% yield) of **10f** were obtained as a yellowish oil from 0.32 mmol of the starting material. It was purified by column chromatography on silica-gel (hexane/AcOEt 9:1). ^{1}H NMR (250 MHz $CDCl_3$, 298 K) 7.45 (m, 3H, Ar*H*), 3.71 (dd, $^2J_{(H,H)}$ = 10.5, $^3J_{(H,F)}$ = 18.5 Hz, 1H, CH_2CF), 3.32 (m, 1H, CH_2CF), 1.44 (s, 9H, OC(CH_3)$_3$); ^{19}F NMR (235 MHz [D]$CDCl_3$, 298 K, TMS) δ = -112.8 (s, 1F), -163.8 pm (s, 1F); HPLC: Daicel Chiralpack AD-H, Hexane/ ^{i}PrOH=99.5:0.5 1.0 mL/min, 254 nm, t_r(minor) = 25.0 min, t_r(major) = 28.6 min (93% *ee*); $[\alpha]_{20}^{D}$: 8.3 (c=6.0 , $CHCl_3$).

Tert-butyl (R)-2-fluoro-5,6-dimethoxy-1-oxo-2,3-dihydro-1H-indene-2-carbox-ylate (**10h**):[12] Following the general procedure, using $Eu(OTf)_3$ and (*S*,*R*)-ind-*pybox*, 57 mg (66% yield) of **10h** were obtained as a yellowish oil from 0.33 mmol of **2h**. It was purified by column chromatography on silica-gel (hexane/AcOEt 9:1). ^{1}H NMR (360 MHz [D]$CDCl_3$, 298 K, TMS) δ = 7.22 (s, 1H, Ar*H*), 6.90 (s, 2H, Ar*H*), 4.01 (s, 3H, OCH_3), 3.94 (s, 3H, OCH_3), 3.64 (dd, $^2J_{(H,H)}$ = 10.5 Hz, $^3J_{(H,F)}$ = 17.6 Hz, 1H), 3.30 (dd, $^2J_{(H,H)}$ = 22.7, $^3J_{(H,F)}$ = 24.2 Hz, 1H), 1.44 ppm (s, 9H, OC(CH_3)$_3$); ^{19}F NMR (235 MHz [D]$CDCl_3$, 298 K, TMS) δ = -163.6 ppm (s, 1F); HPLC: Daicel Chiralpack AD-H,

Hexane/^{i}PrOH=85:15, 0.5 mL/min, 254 nm, t_r(major) = 17.1 min, t_r(minor) = 19.6 min (91% *ee*); $[\alpha]_{20}^{D}$: -34.4 (c=6.1 , $CHCl_3$).

5. Synthesis of *pybox* ligands 4 and 5

To and argon purged solution of 2,6-pyridine carbonitrile (0.8 g, 6 mmol, 1 eq) in dry methanol (25 mL) was added 0.001 g of sodium, and the solution was stirred at room temperature for 48 hours. Glacial acetic acid (0.05 mL) was subsequently added, and the solution was stirred for additional 5 minutes. Then the solution was dried in vacuo affording an off white solid. This intermediate was reintroduced in dry dichloromethane (30 mL) and the desired amino-alcohol (13.4 mmol, 2.2 eq) was added in one portion to the solution. The mixture was refluxed under inert atmosphere for 4 hours, and then stirred for 48 h at room temperature. The mixture was concentrated in vacuo and filtered from MeOH. The solid was then collected, dissolved in dichloromethane, dried over Na_2SO_4 and concentrated under vacuum, affording the final *pybox* ligand.

2,6-Bis[(4S,5R)-4,5-diphenyl-4,5-dihydro-1,3-oxazol-2-yl]pyridine (**4**):[16] ^{1}H NMR (250 MHz [D]$CDCl_3$, 298 K, TMS) δ = 8.39 (d, $^3J_{(H,H)}$ = 8.5 Hz, 2H, Ar*H*), 8.04 (t, *J* = 8.5 Hz, 1H, Ar*H*), 7.03-7,09 (m, 20H, *Ph*), 6.14 (d, *J* = 10.5 Hz, 2H, C*H*O), 5.84 (d, *J* = 10.5 Hz, 2H, C*H*N). ^{13}C NMR (63 MHz [D]$CDCl_3$, 298 K, TMS) δ = 164.2, 138.1, 137.9, 136.5, 128.4, 128.1, 127.9, 127.5, 127.2, 127.0, 126.9, 86.7, 74.9.

2,6-bis[(4*R*,5*R*)-4,5-diphenyl-4,5-dihydro-1,3-oxazol-2-yl]pyridine (**5**):[14b] ^{1}H NMR (360 MHz [D]$CDCl_3$, 298 K, TMS) δ = 8.39 (d, *J* = 9.5 Hz, 2H, Ar*H*), 7.98 (t, *J* = 9.5 Hz, 1H, Ar*H*), 7.30-7,37 (m, 20H, *Ph*), 5.54 (d, *J* = 8.5 Hz, 2H, C*H*O), 5.33 (d, *J* = 8.5 Hz, 2H, C*H*N). ^{13}C NMR (91 MHz [D]$CDCl_3$, 298 K, TMS) δ = 163.8, 142.1, 140.6, 138.2, 129.6, 129.4, 128.7, 127.7, 127.4, 127.0, 90.9, 79.9.

6. Spectra, HPLC and CD of compounds

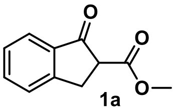

1a

^{1}H NMR (400 MHz, $CDCl_3$)

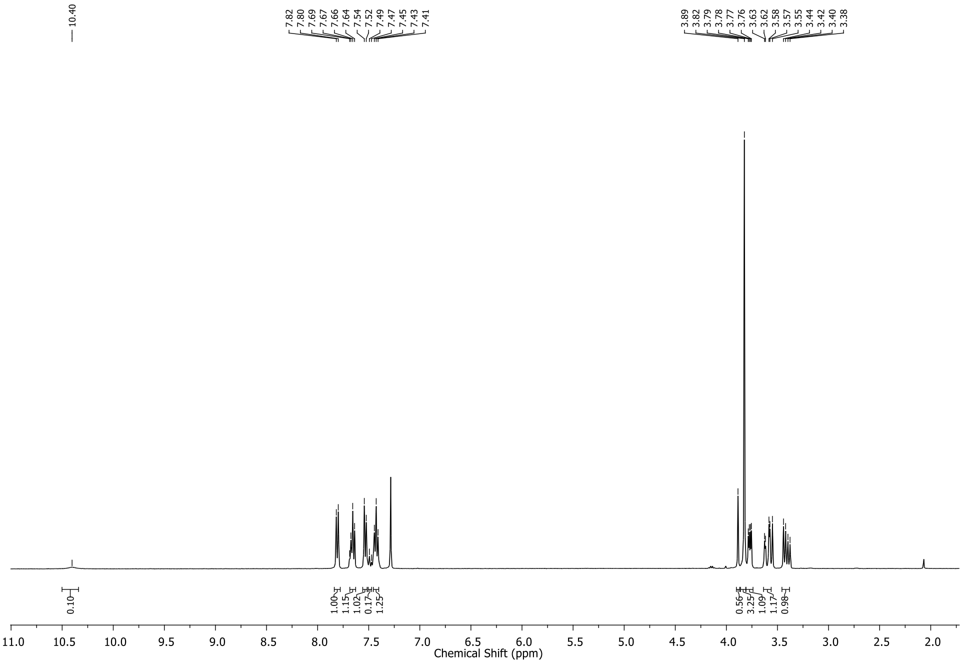

^{13}C[^{1}H] NMR (91 MHz, $CDCl_3$)

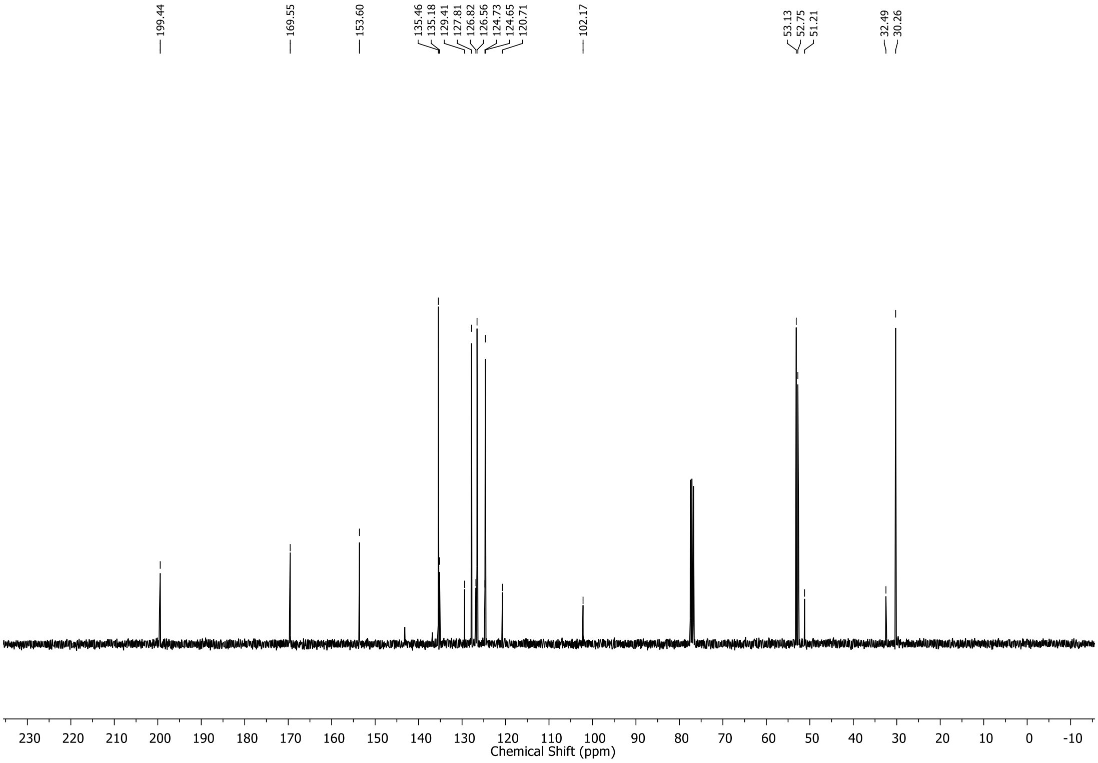

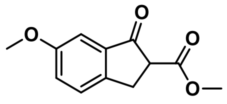

1e

^{1}H NMR (250 MHz, CDCl$_3$)

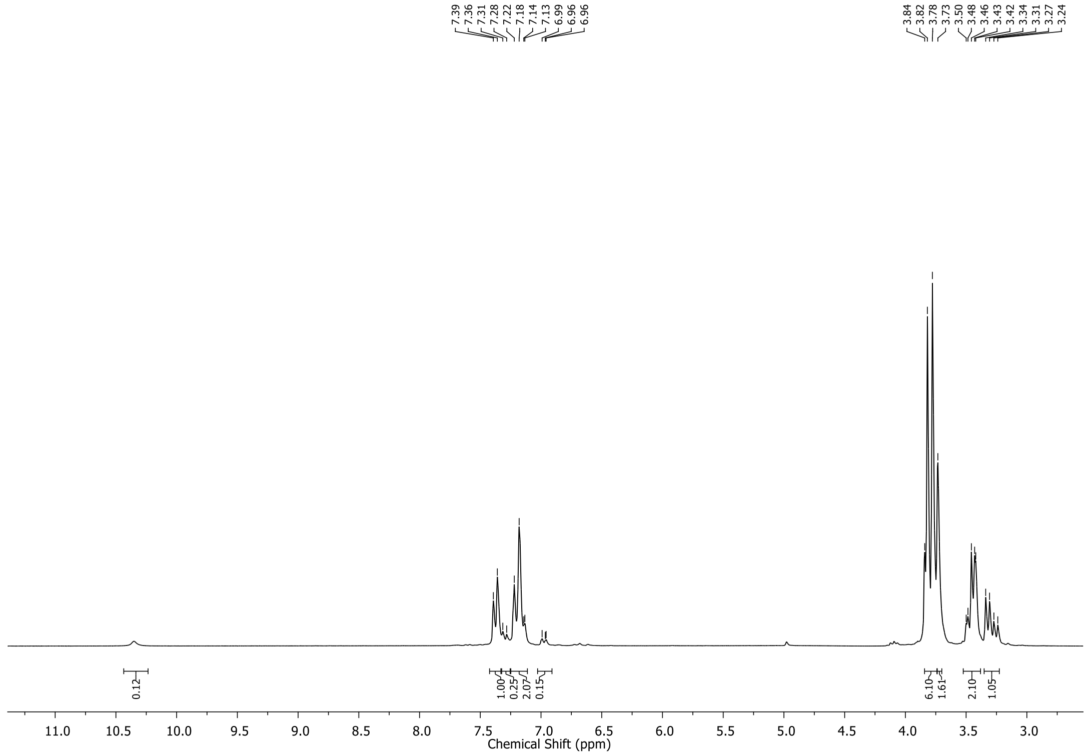

^{13}C[^{1}H] NMR (63 MHz, CDCl$_3$)

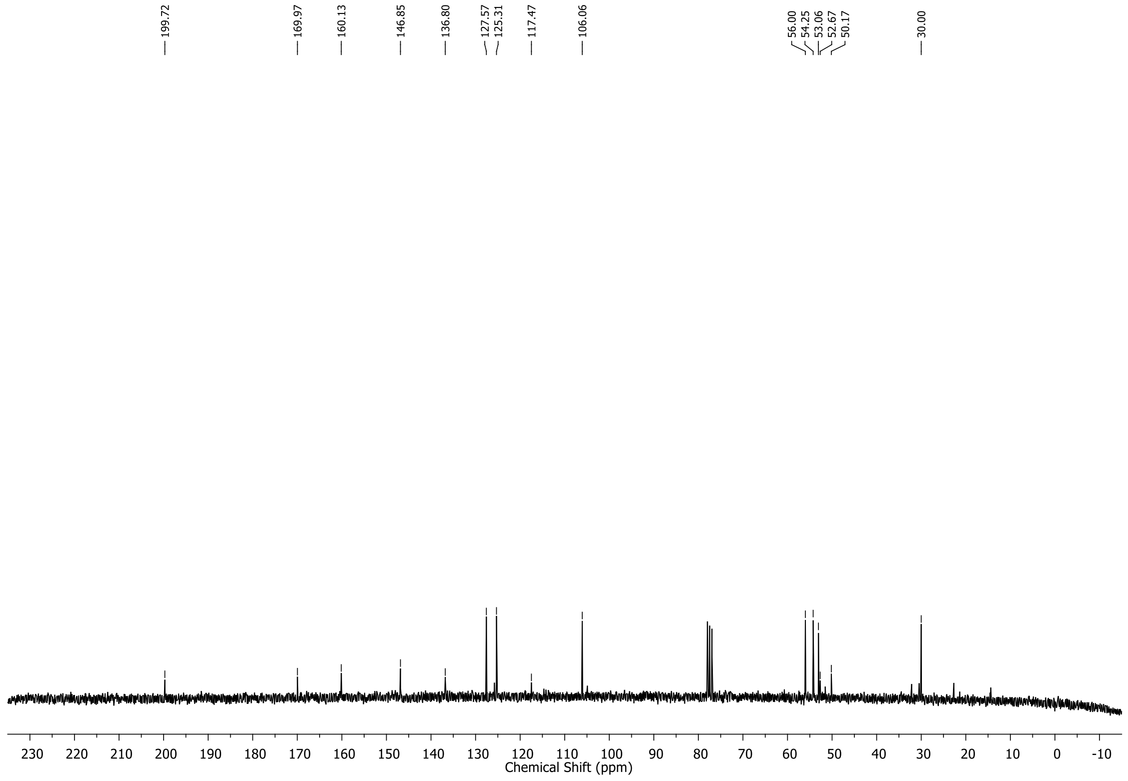

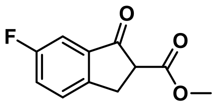

1f

^{1}H NMR (250 MHz, $CDCl_3$)

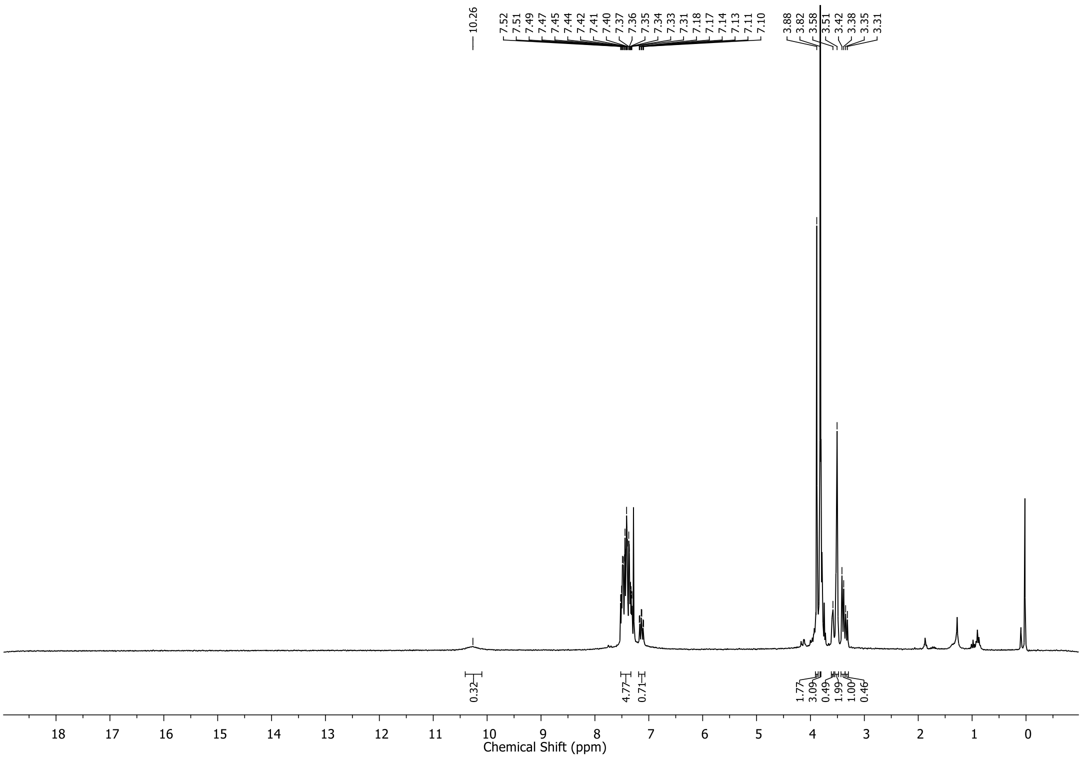

^{13}C[^{1}H] NMR (91 MHz, $CDCl_3$)

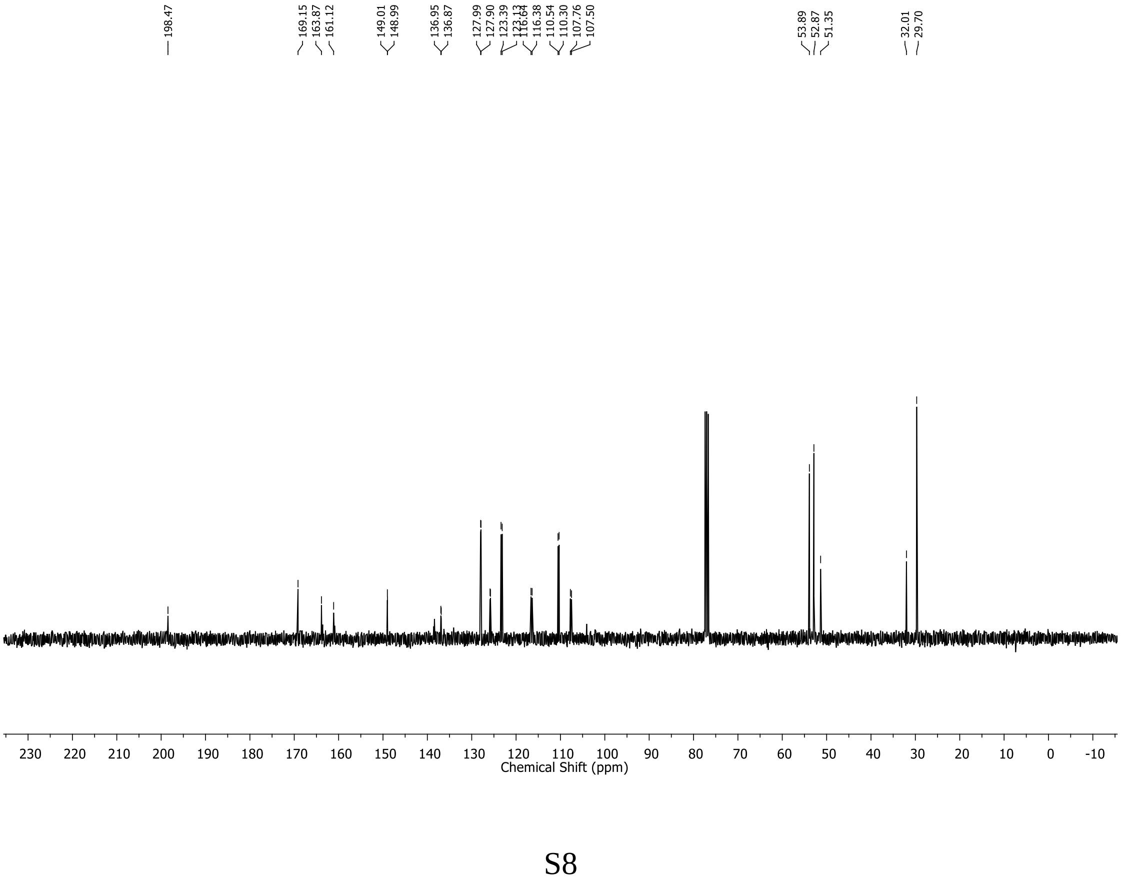

^{19}F NMR (236 MHz, $CDCl_3$)

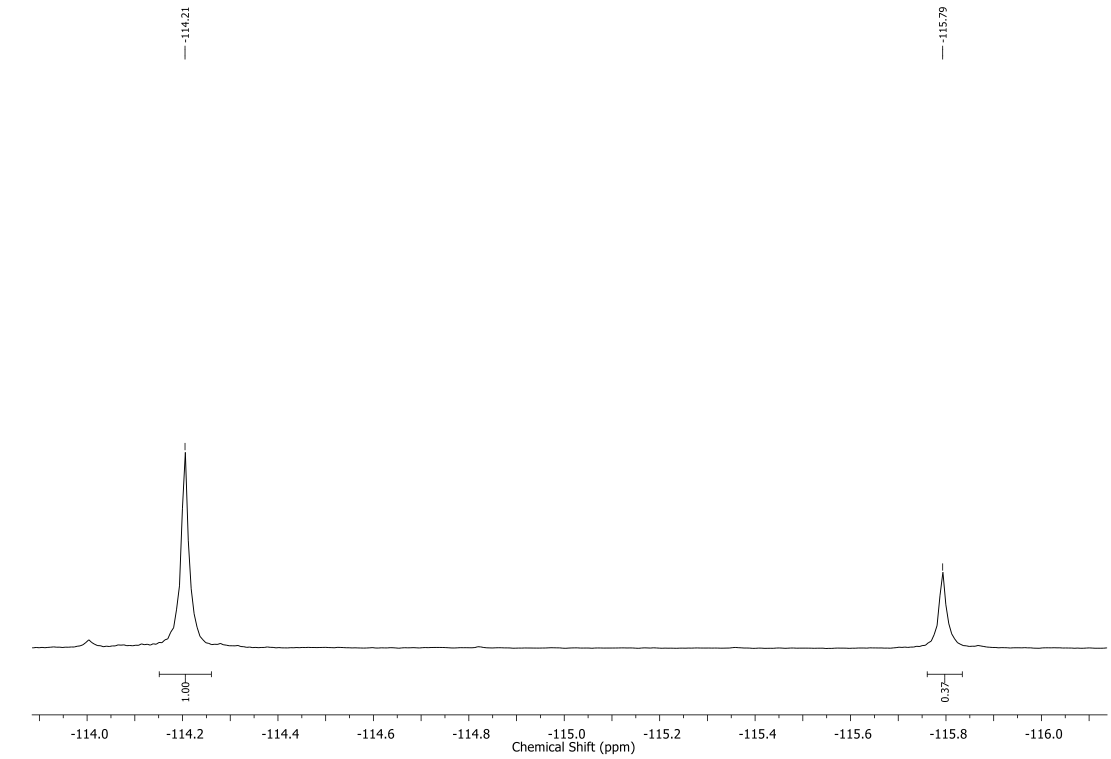

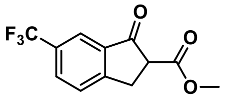

1g

^{1}H NMR (400 MHz, $CDCl_3$)

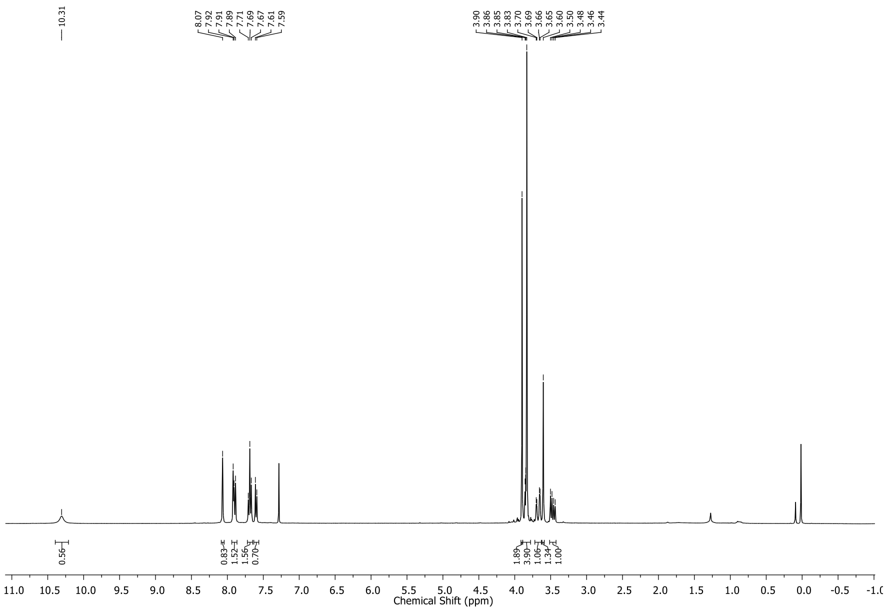

^{19}F NMR (236 MHz, $CDCl_3$)

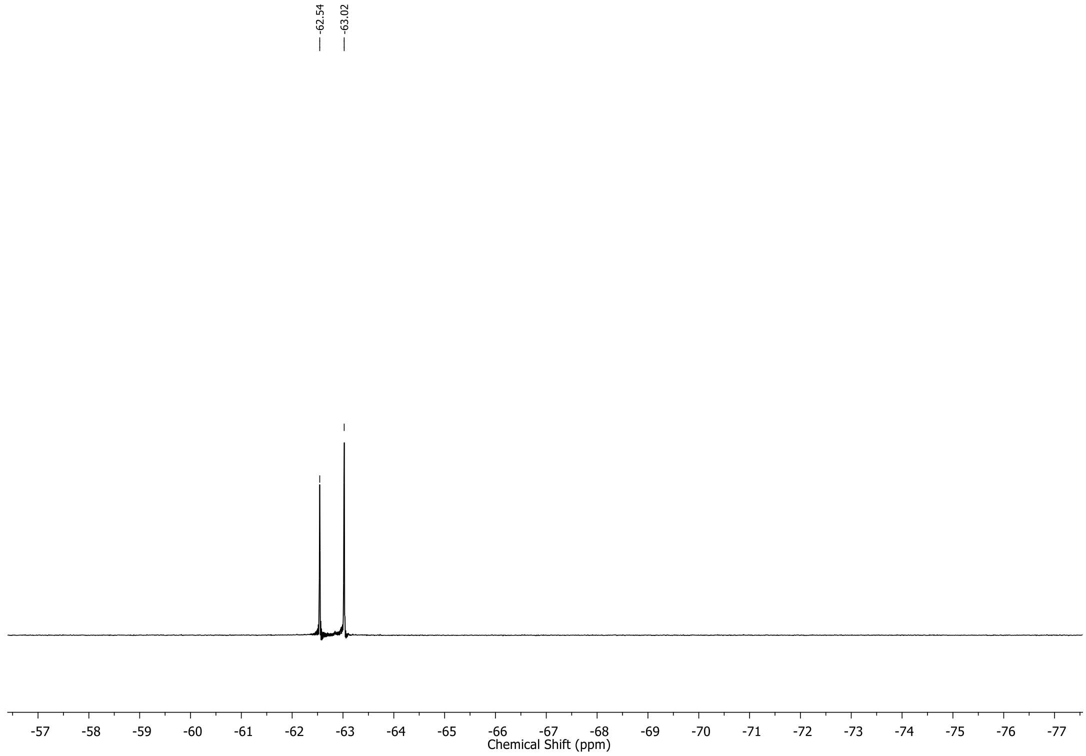

^{13}C[^{1}H] NMR (101 MHz, $CDCl_3$)

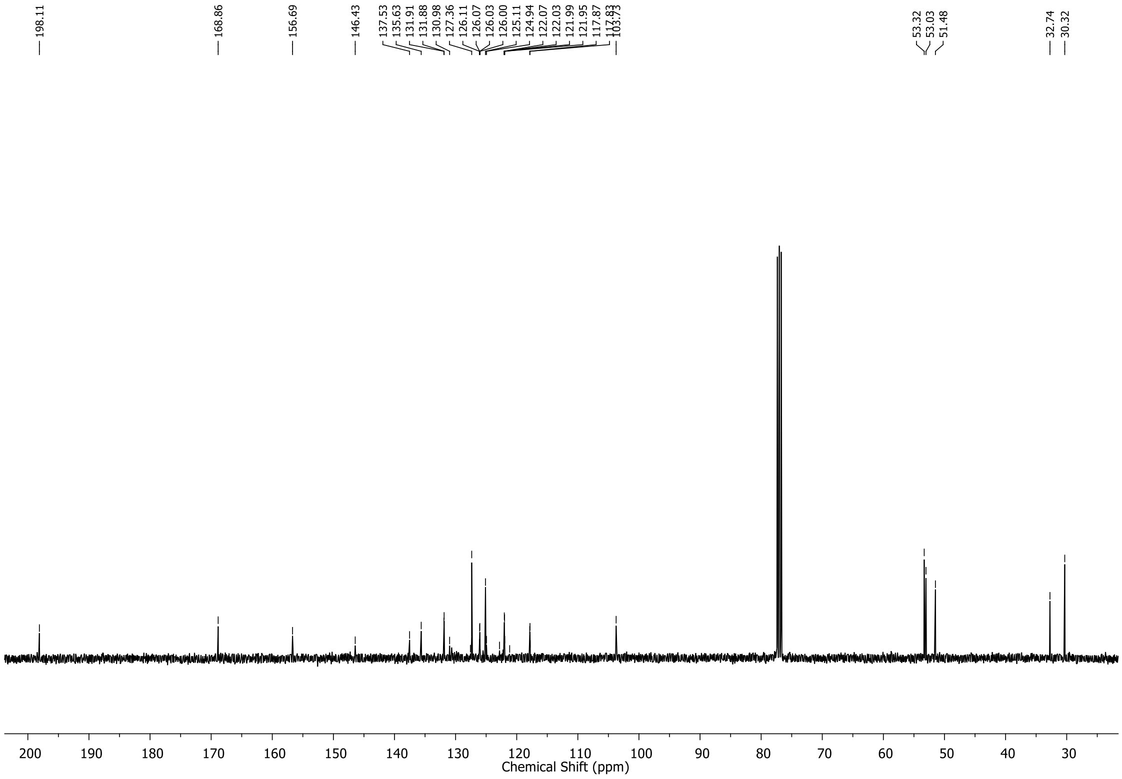

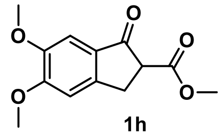

1h

^{1}H NMR (360 MHz, $CDCl_3$)

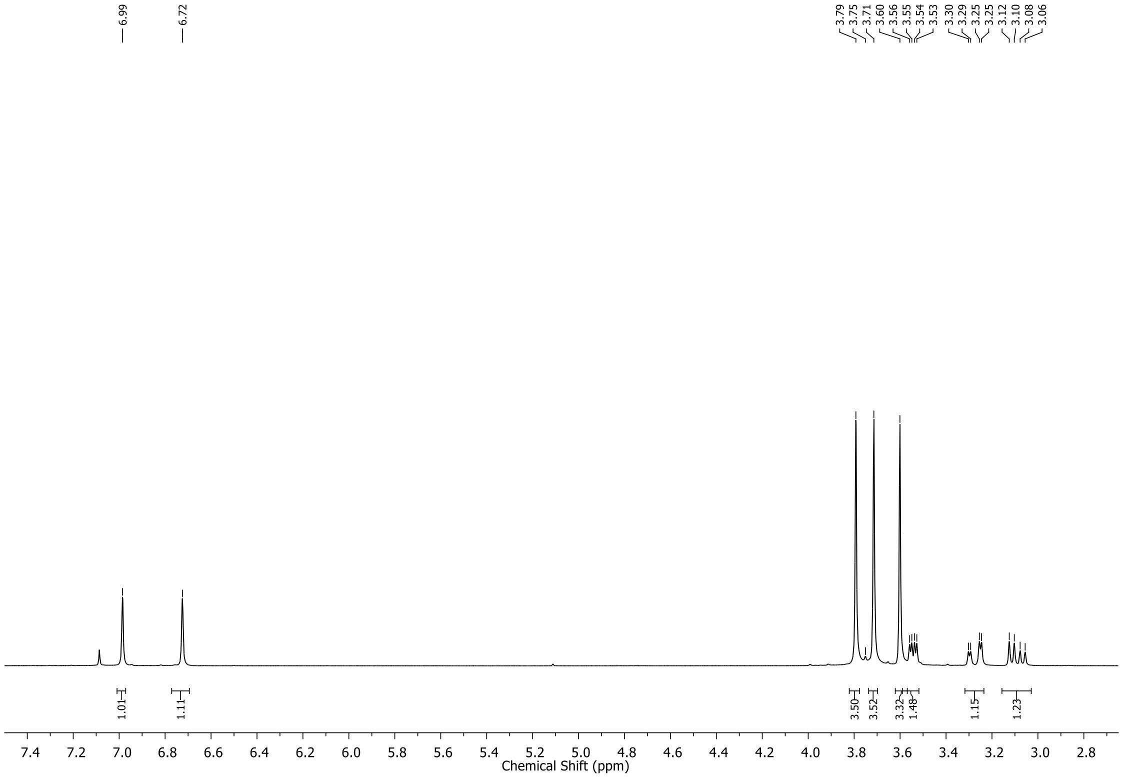

^{13}C[^{1}H] NMR (91 MHz, $CDCl_3$)

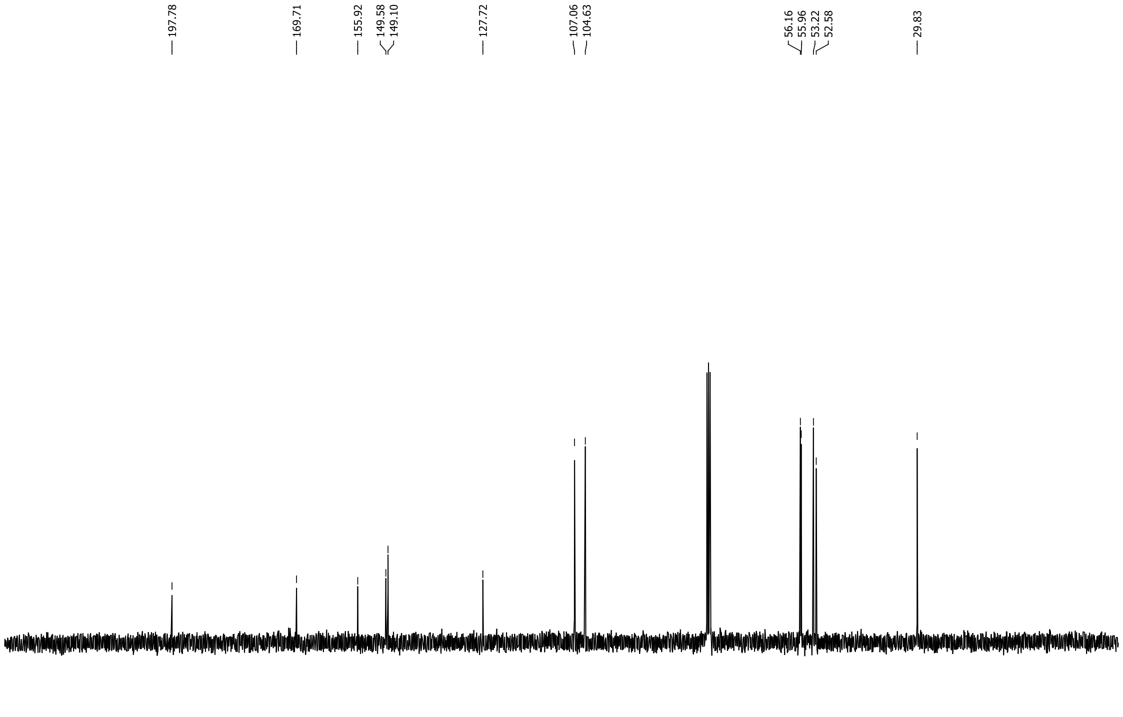

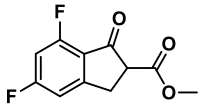

1i

^{1}H NMR (360 MHz, $CDCl_3$)

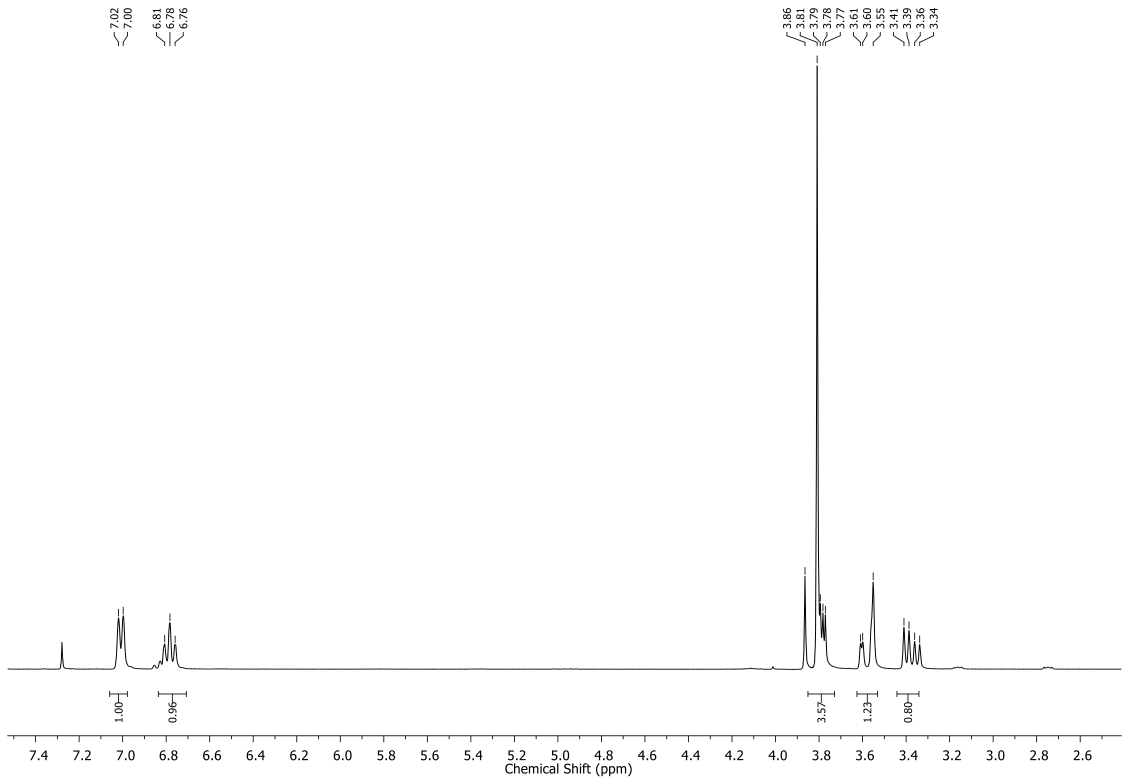

^{19}F NMR (236 MHz, $CDCl_3$)

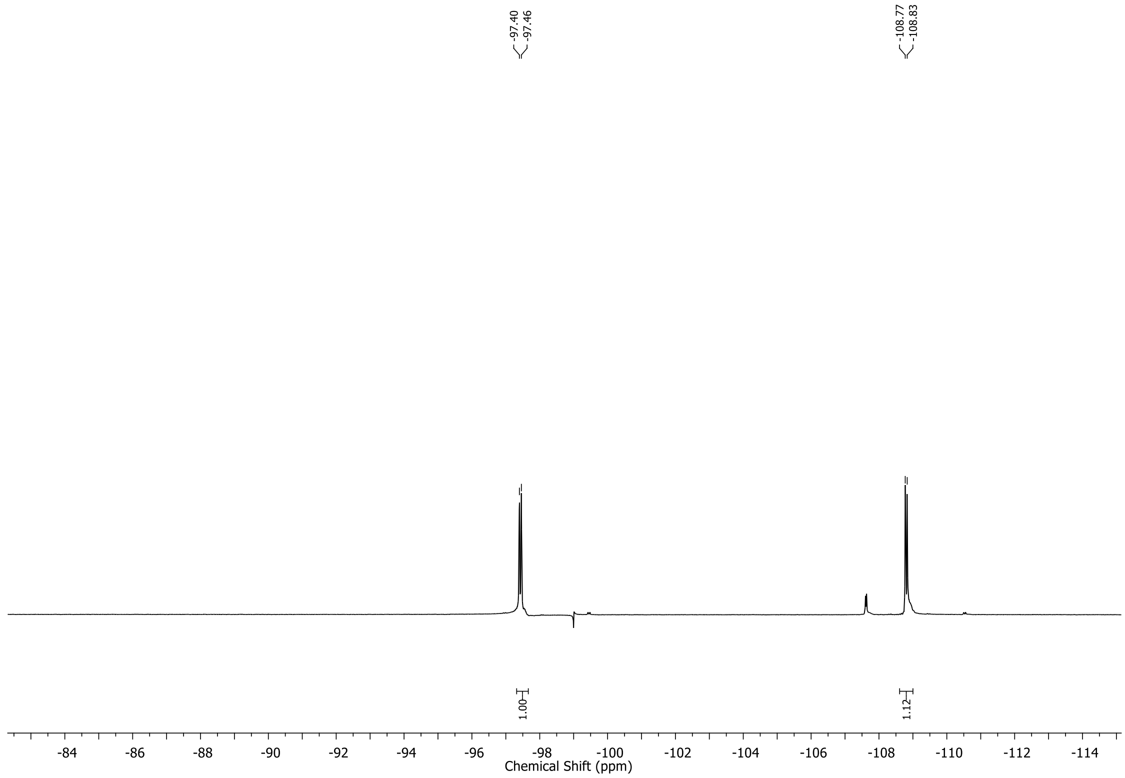

$^{13}C[^1H]$ NMR (91 MHz, $CDCl_3$)

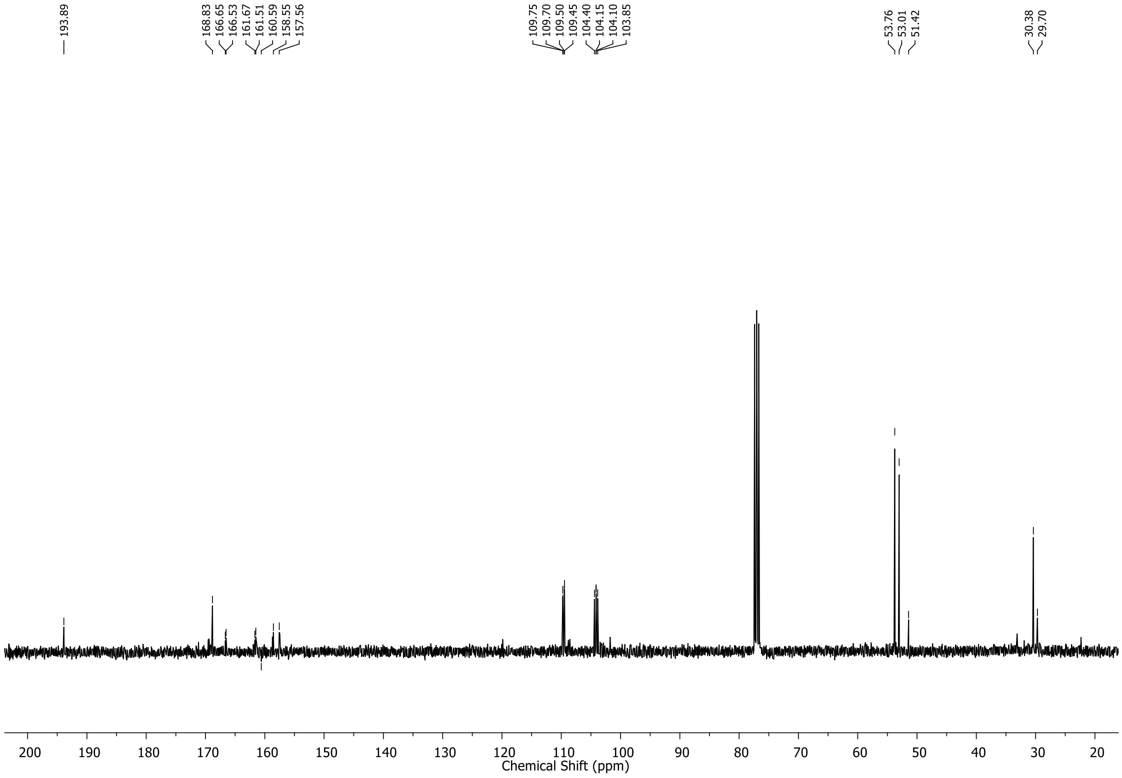

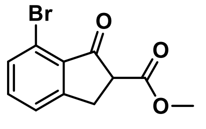

1j

^1H NMR (250 MHz, $CDCl_3$)

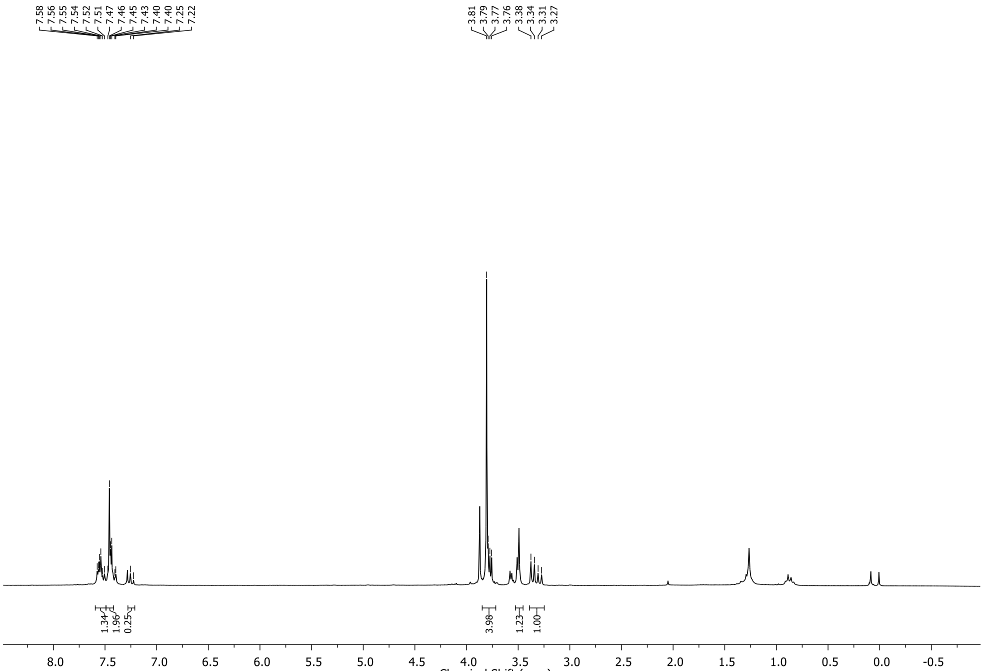

$^{13}C[^{1}H]$ NMR (63 MHz, $CDCl_3$)

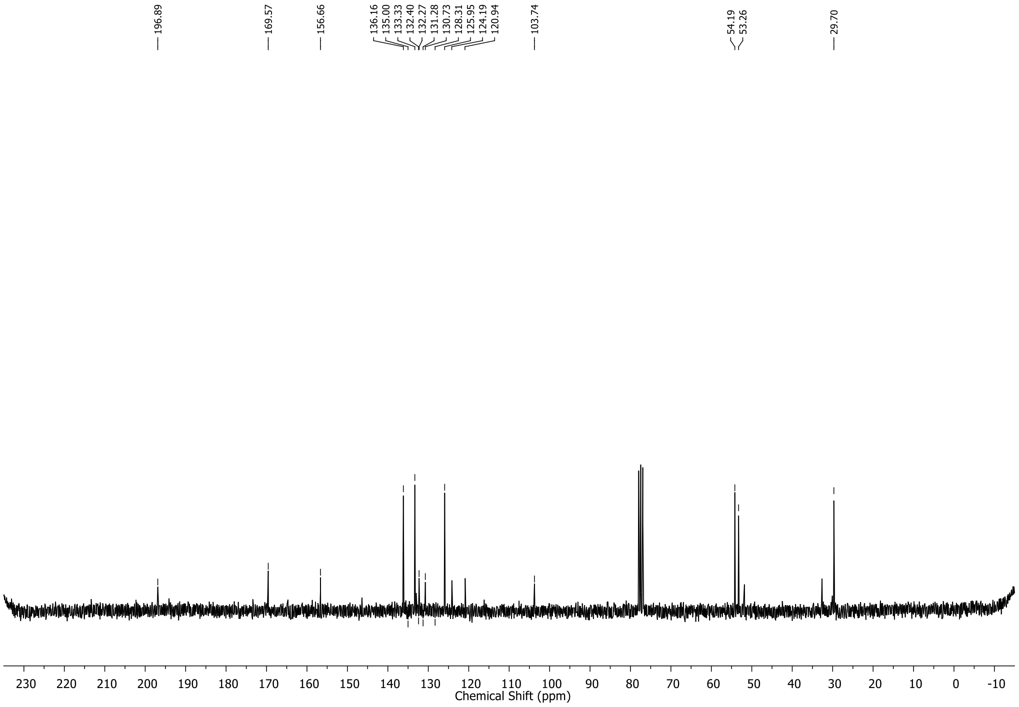

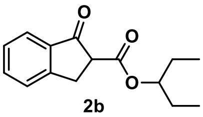

2b

^{1}H NMR (400 MHz, $CDCl_3$)

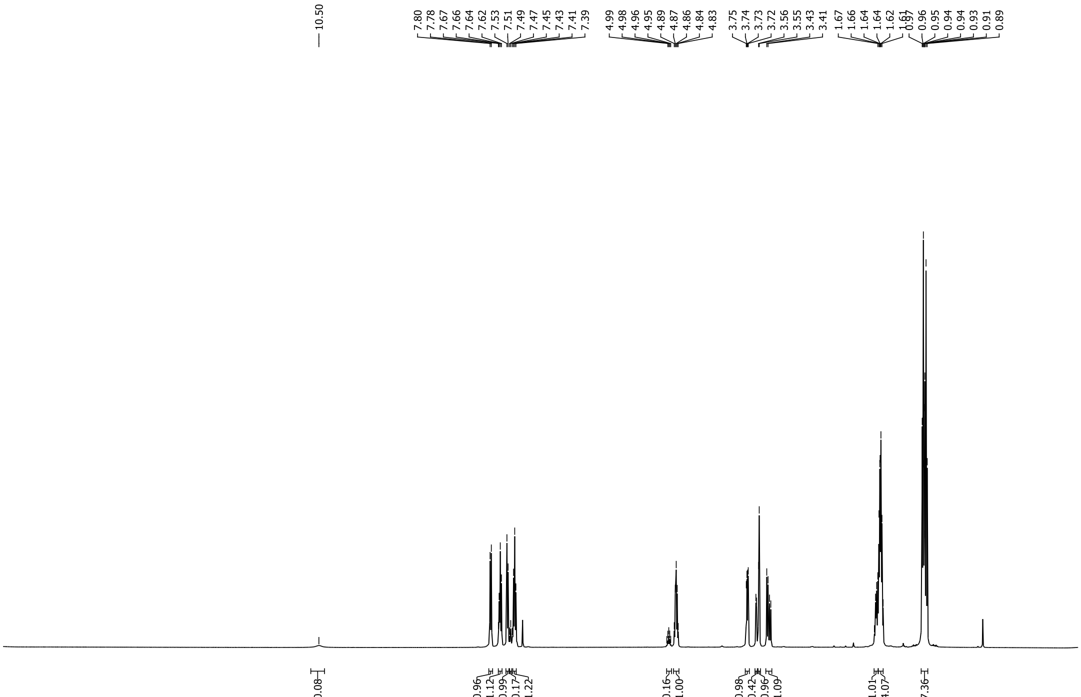

$^{13}C[^{1}H]$ NMR (101 MHz, $CDCl_3$)

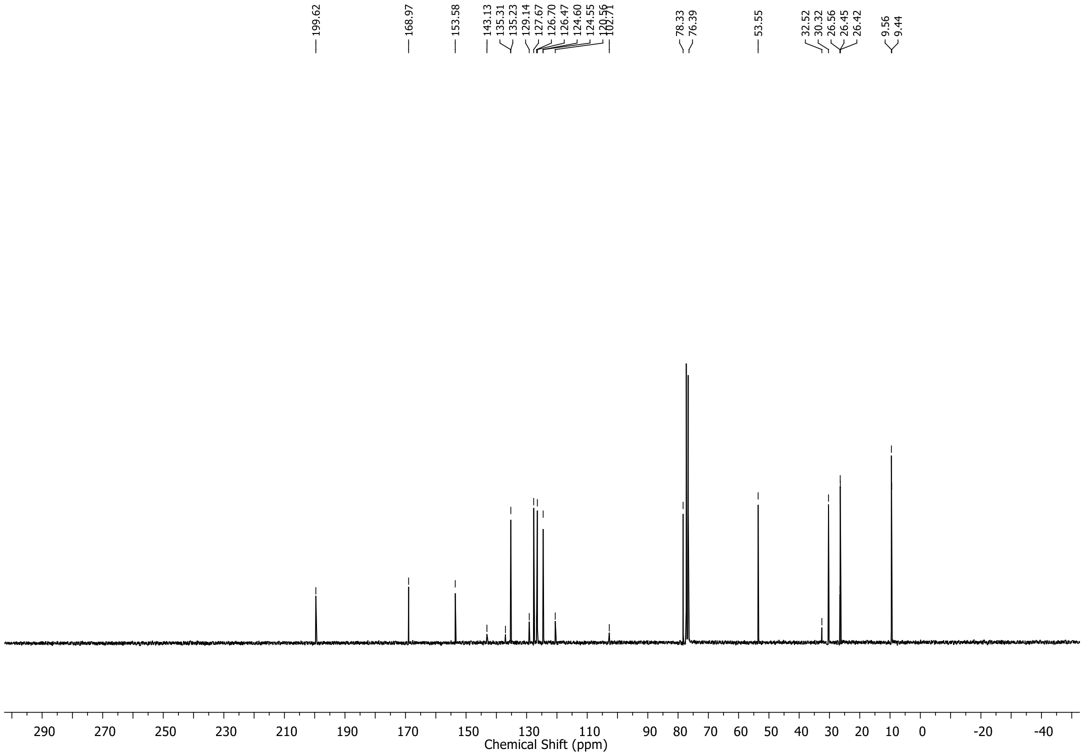

IR (ATR)

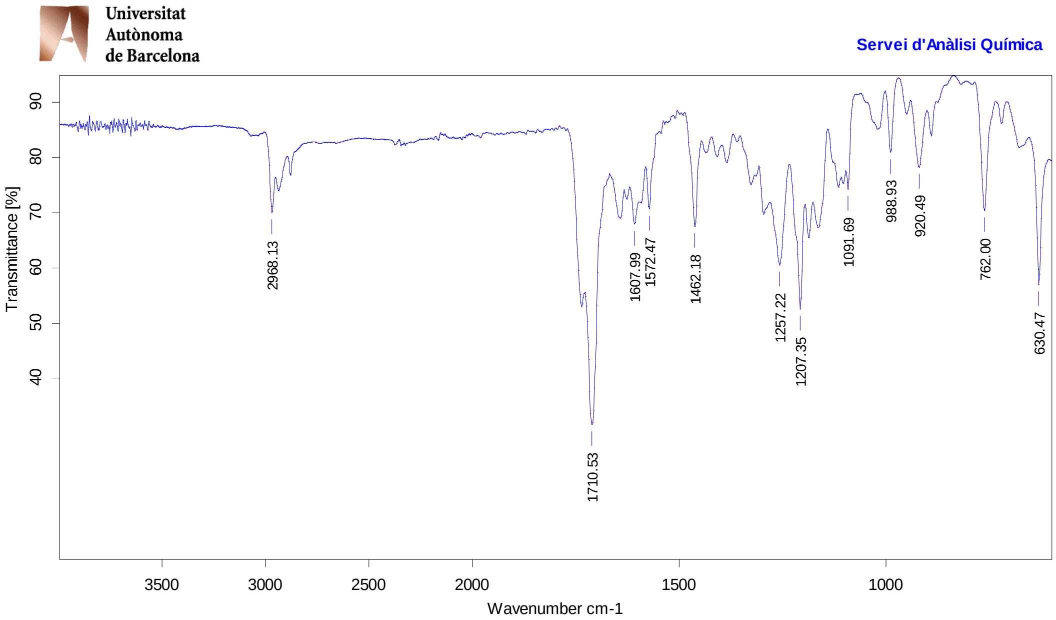

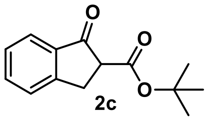

2c

^{1}H NMR (360 MHz, $CDCl_3$)

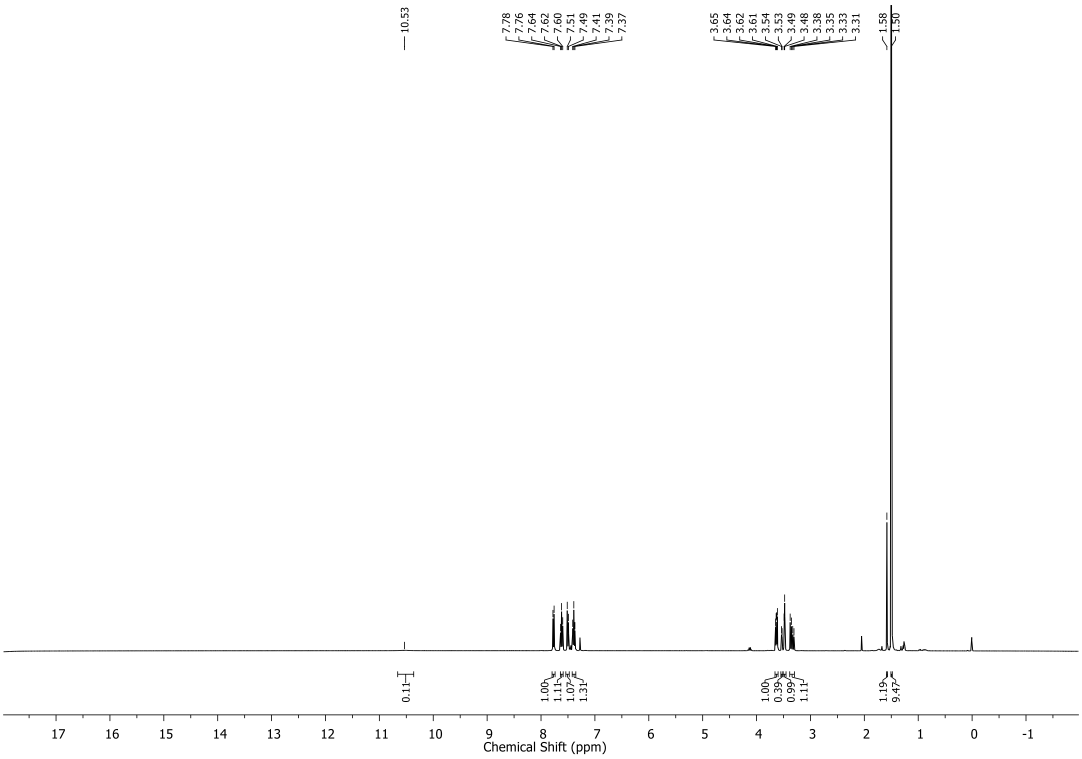

^{13}C[^{1}H] NMR (101 MHz, $CDCl_3$)

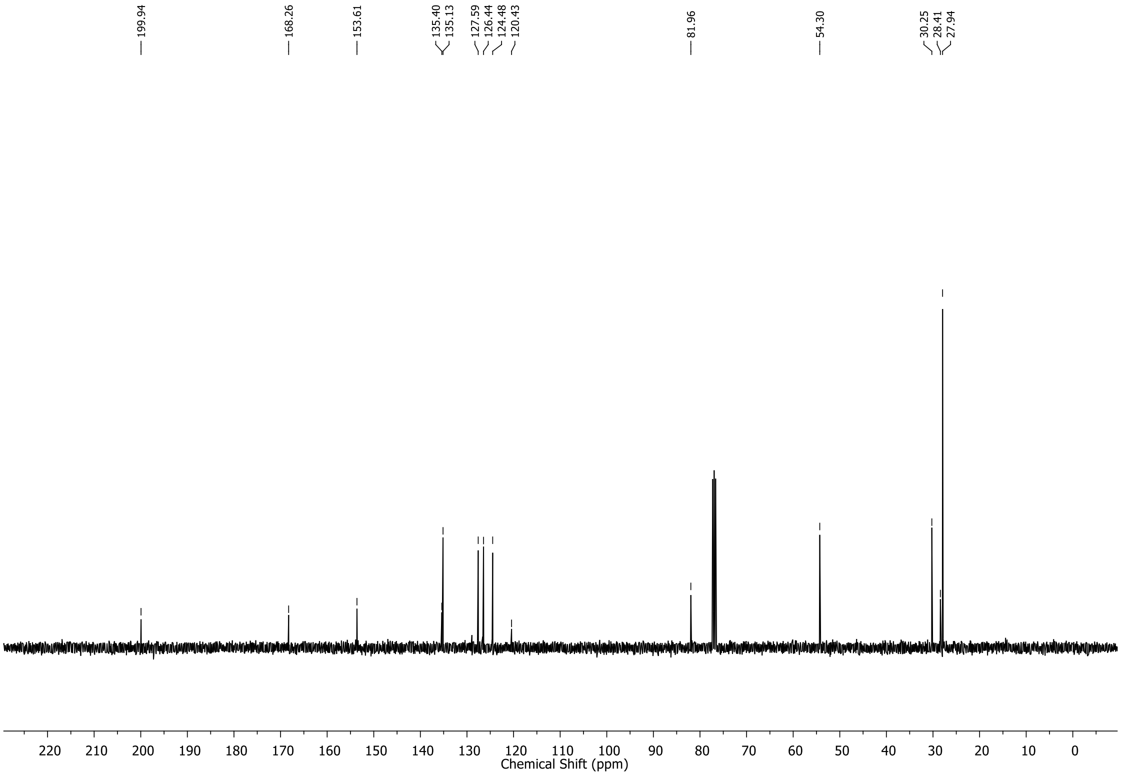

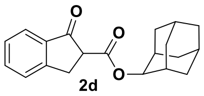

^{1}H NMR (360 MHz, $CDCl_3$)

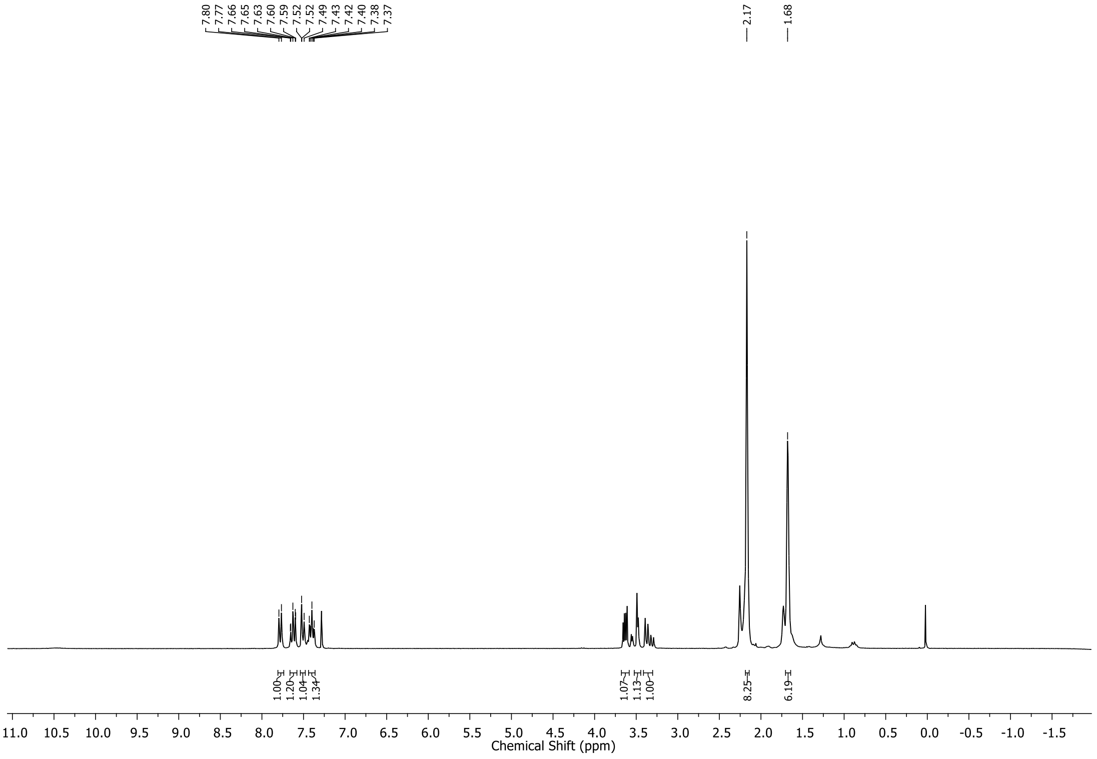

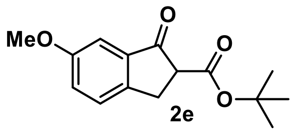

^{1}H NMR (250 MHz, $CDCl_3$)

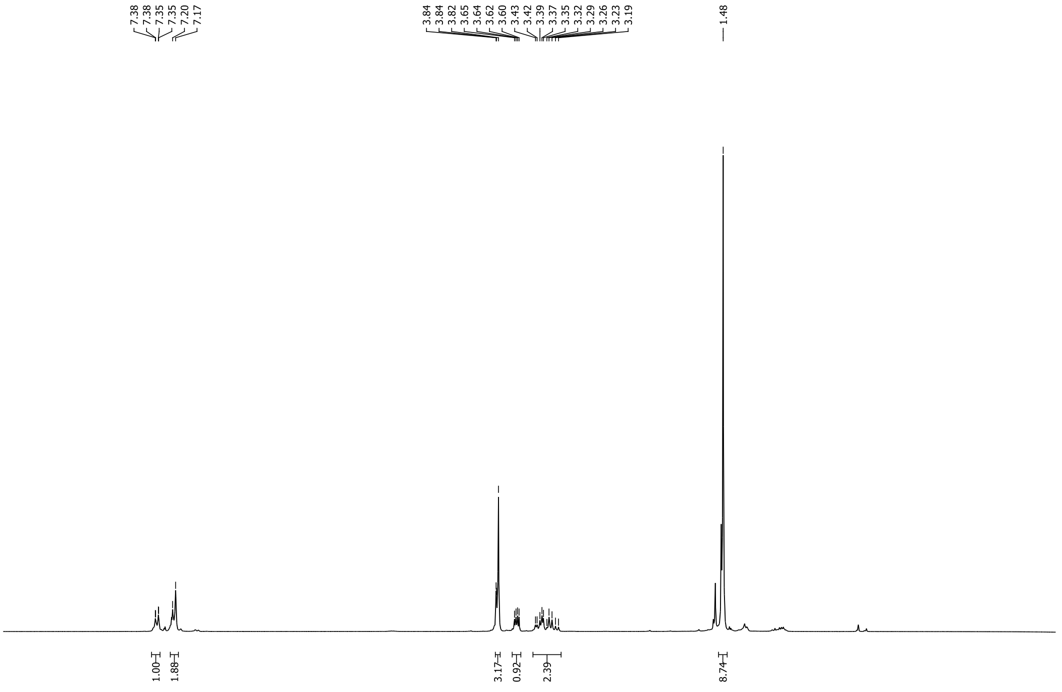

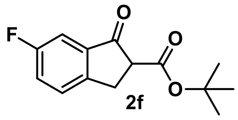

2f

^{1}H NMR (250 MHz, CDCl$_3$)

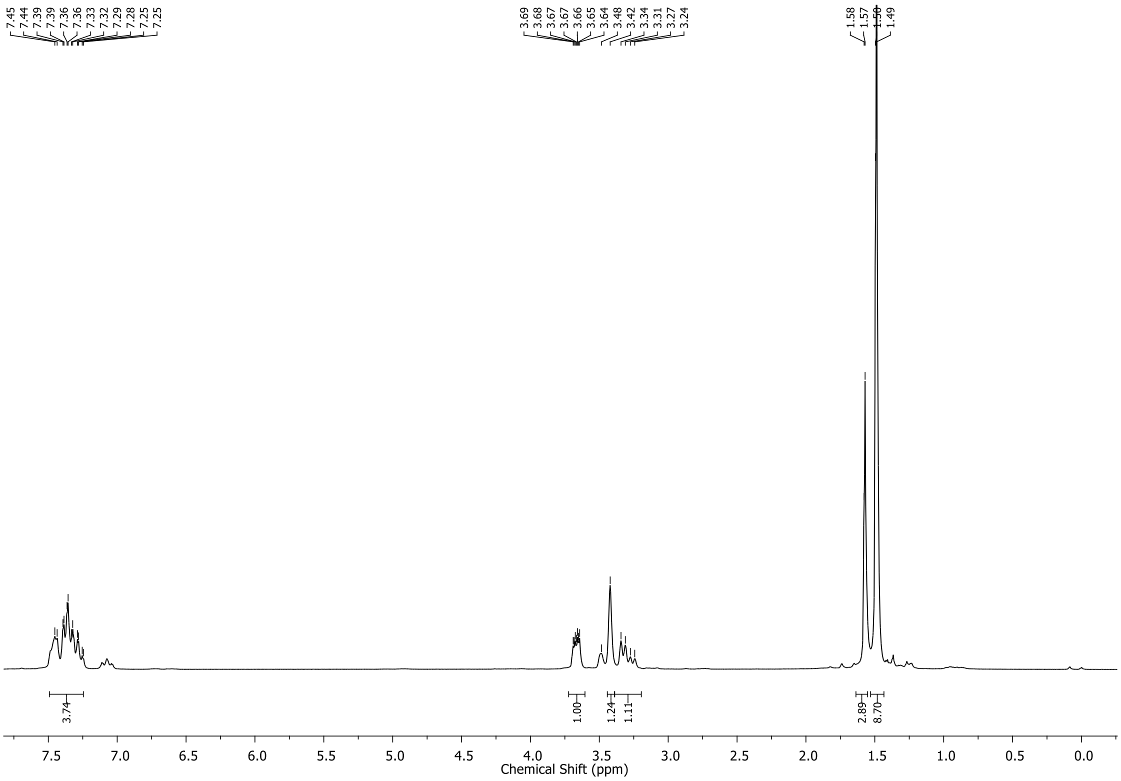

^{19}F NMR (235 MHz, CDCl$_3$)

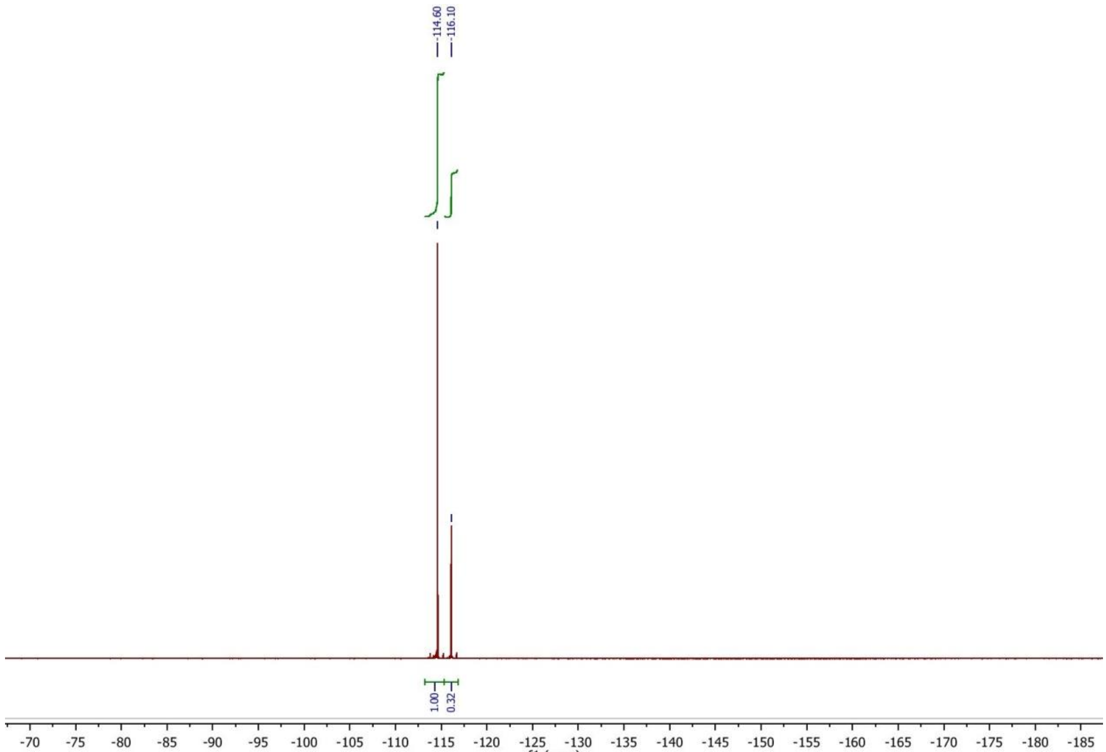

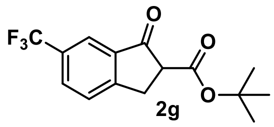

^{1}H NMR (250 MHz, $CDCl_3$)

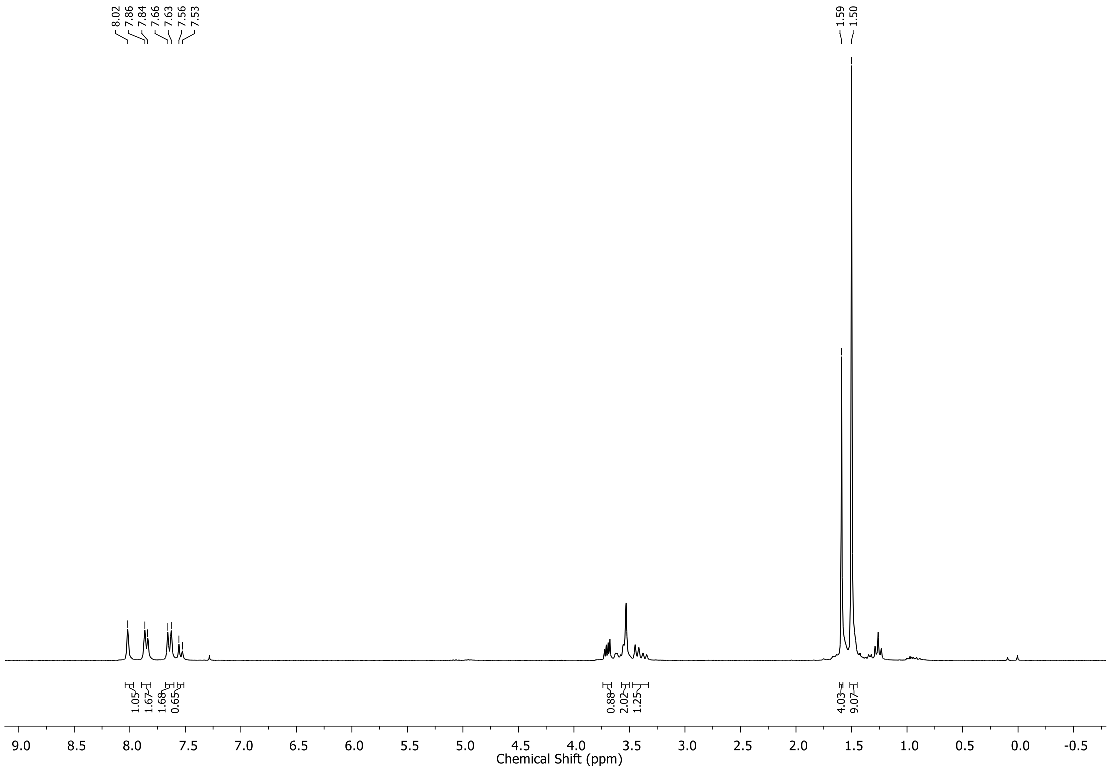

^{19}F NMR(235 MHz, CDCl$_3$)

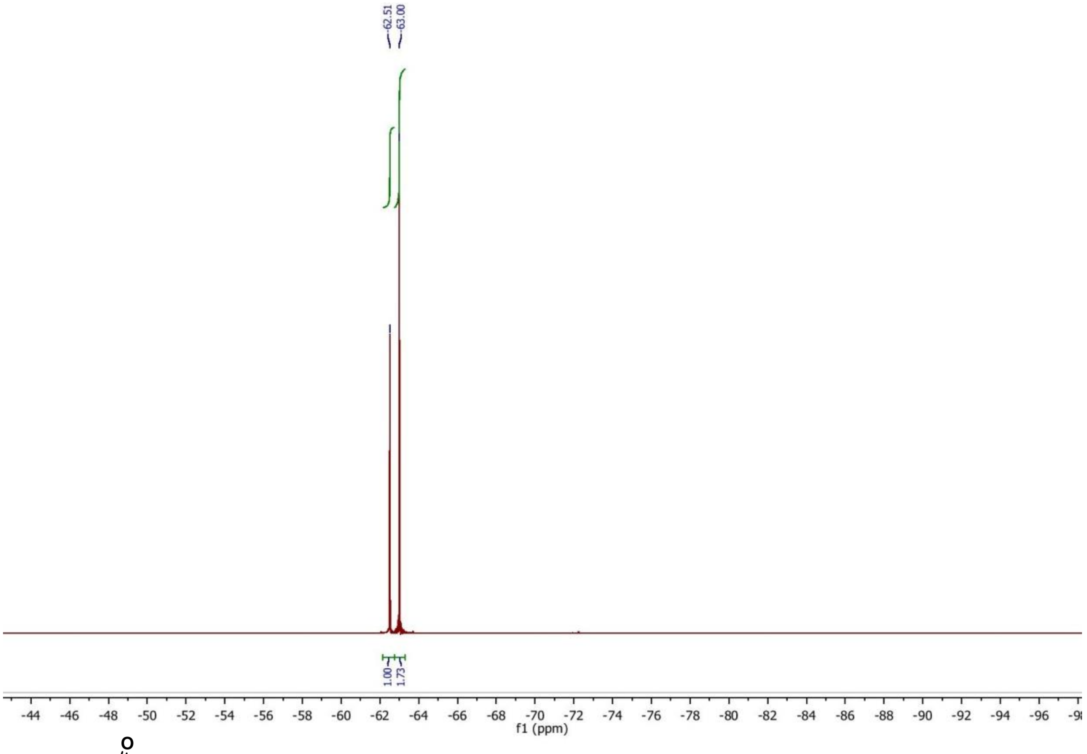

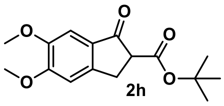

2h

^{1}H NMR (250 MHz, CDCl$_3$)

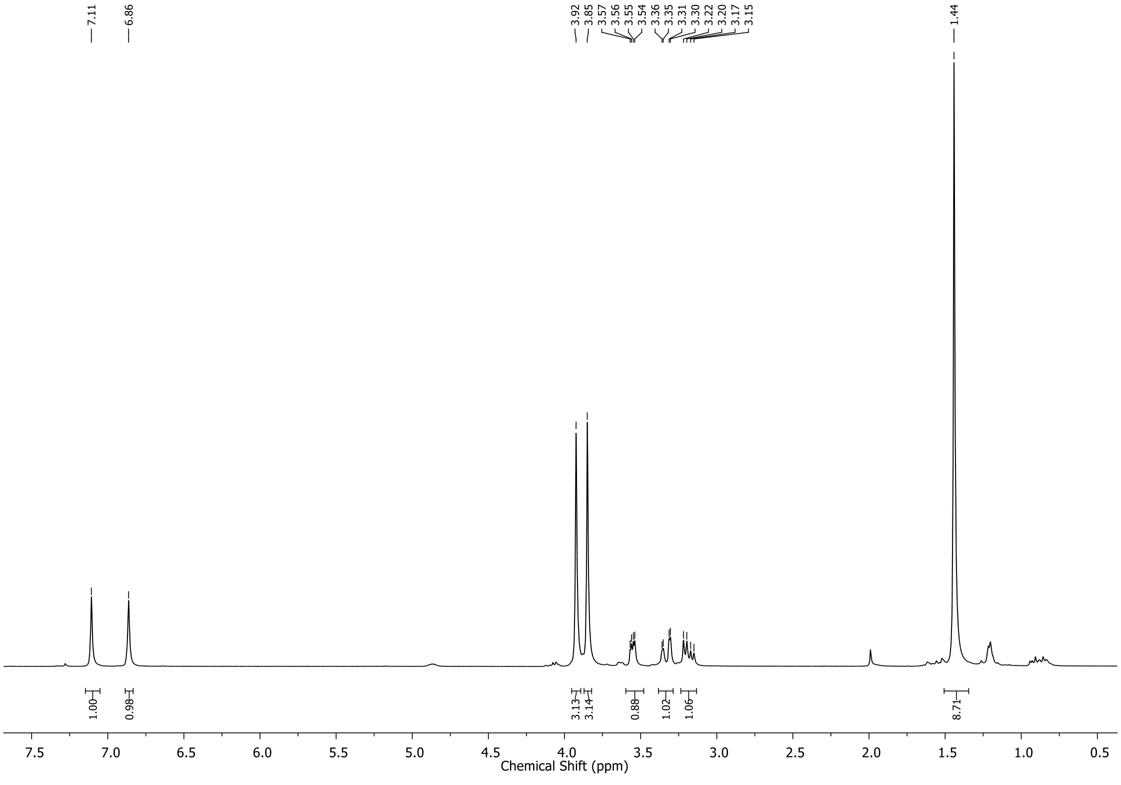

^{13}C NMR (63 MHz, $CDCl_3$)

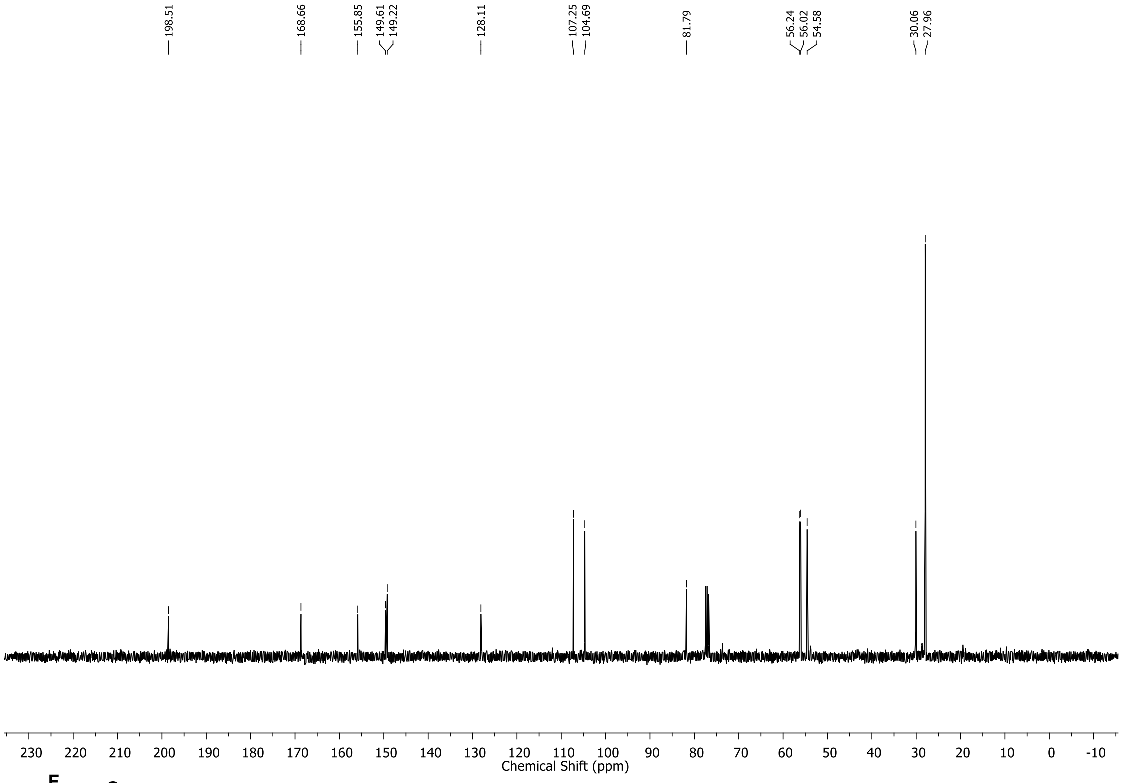

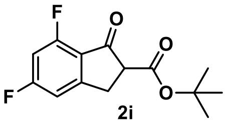

2i

^{1}H NMR (360 MHz, $CDCl_3$)

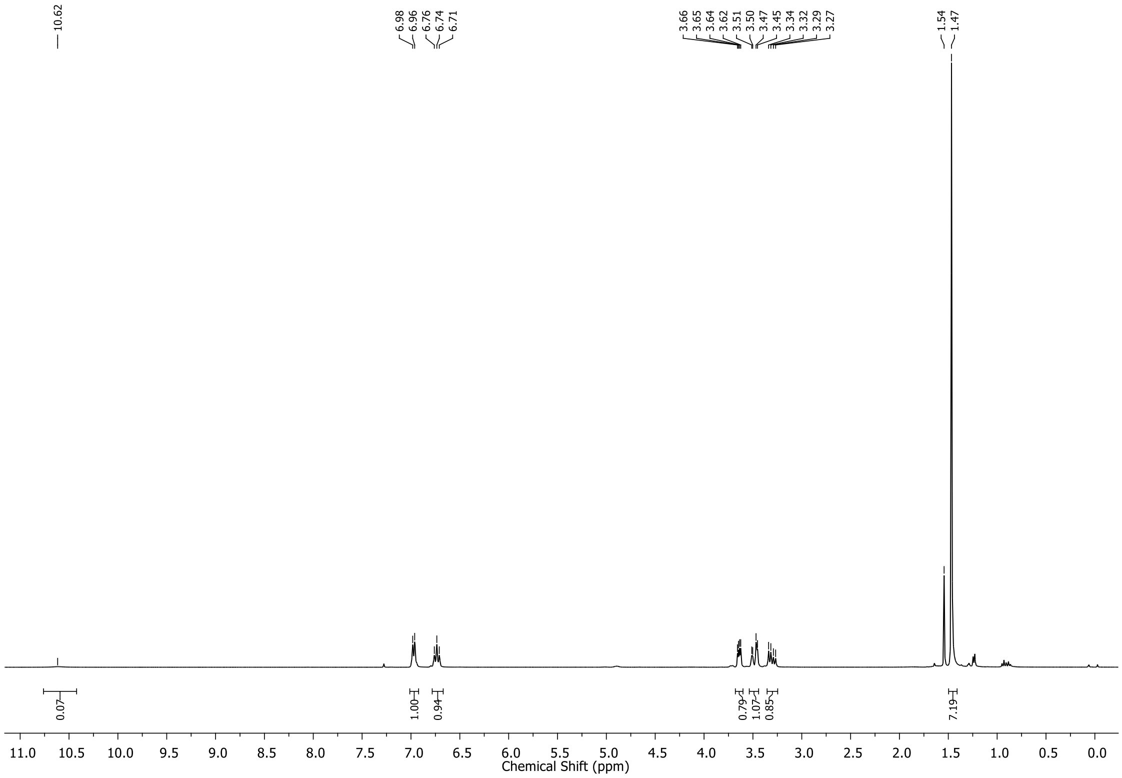

^{13}C NMR (91 MHz, $CDCl_3$)

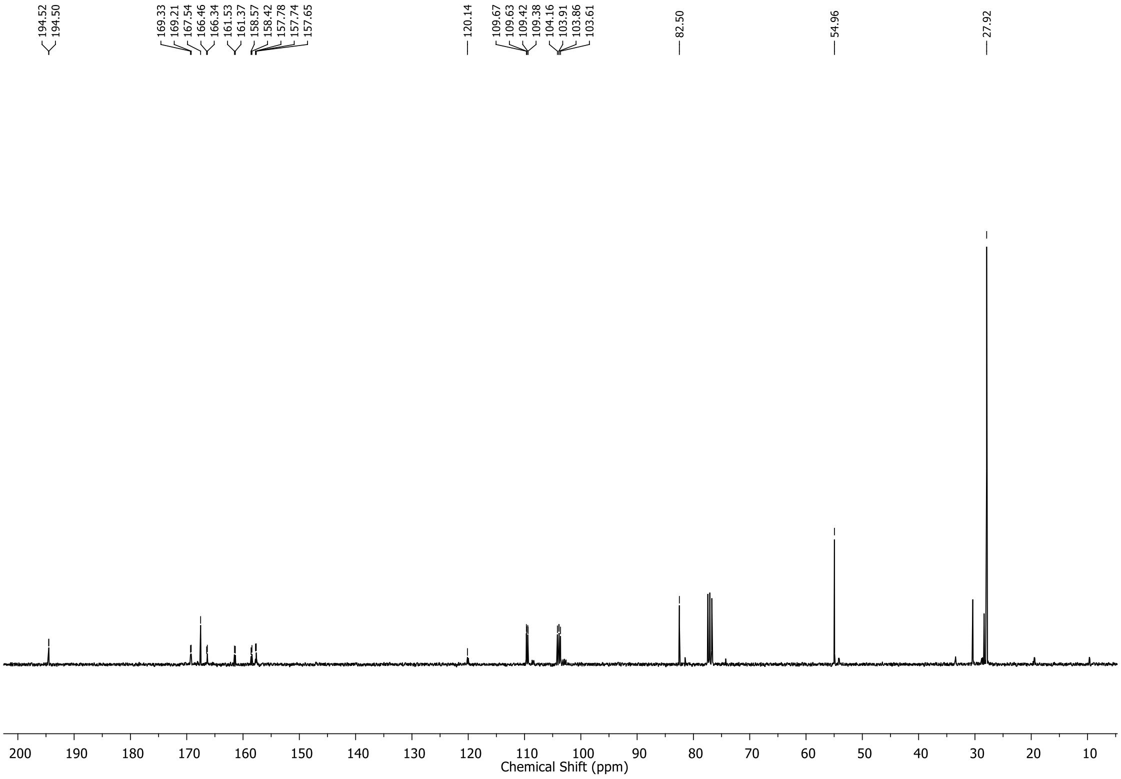

^{19}F NMR (235 MHz, $CDCl_3$)

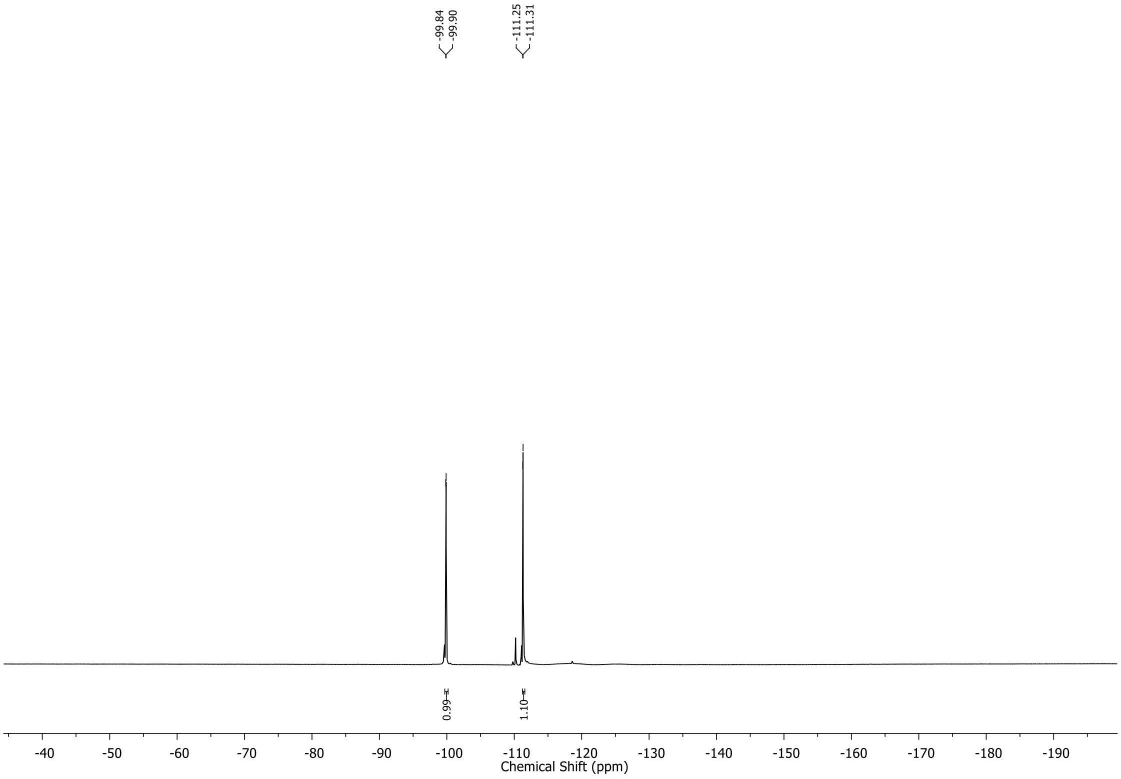

IR (ATR)

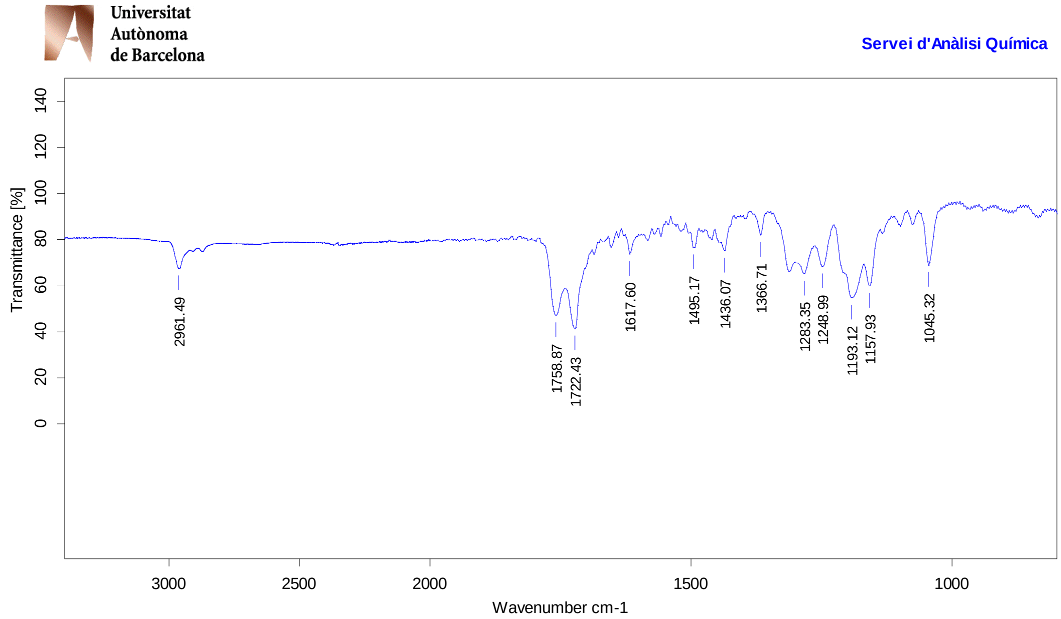

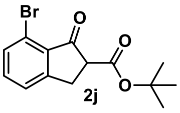

^{1}H NMR (250 MHz, CDCl$_3$)

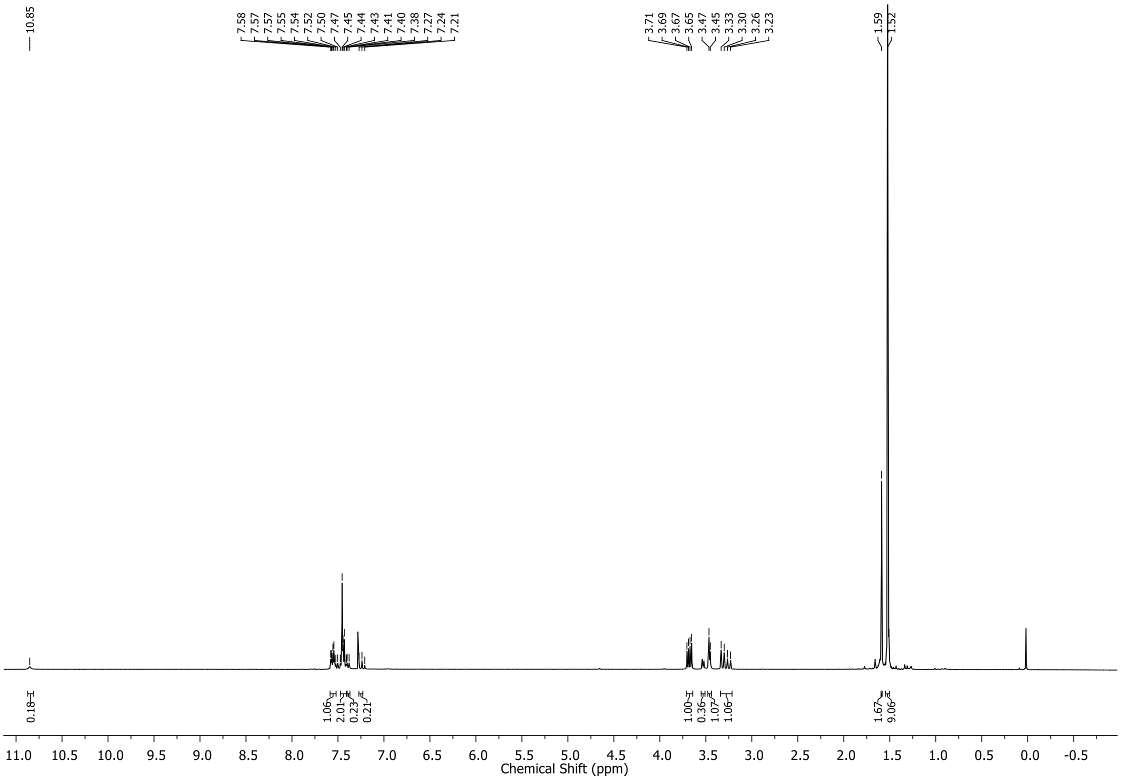

^{13}C NMR (63 MHz, $CDCl_3$)

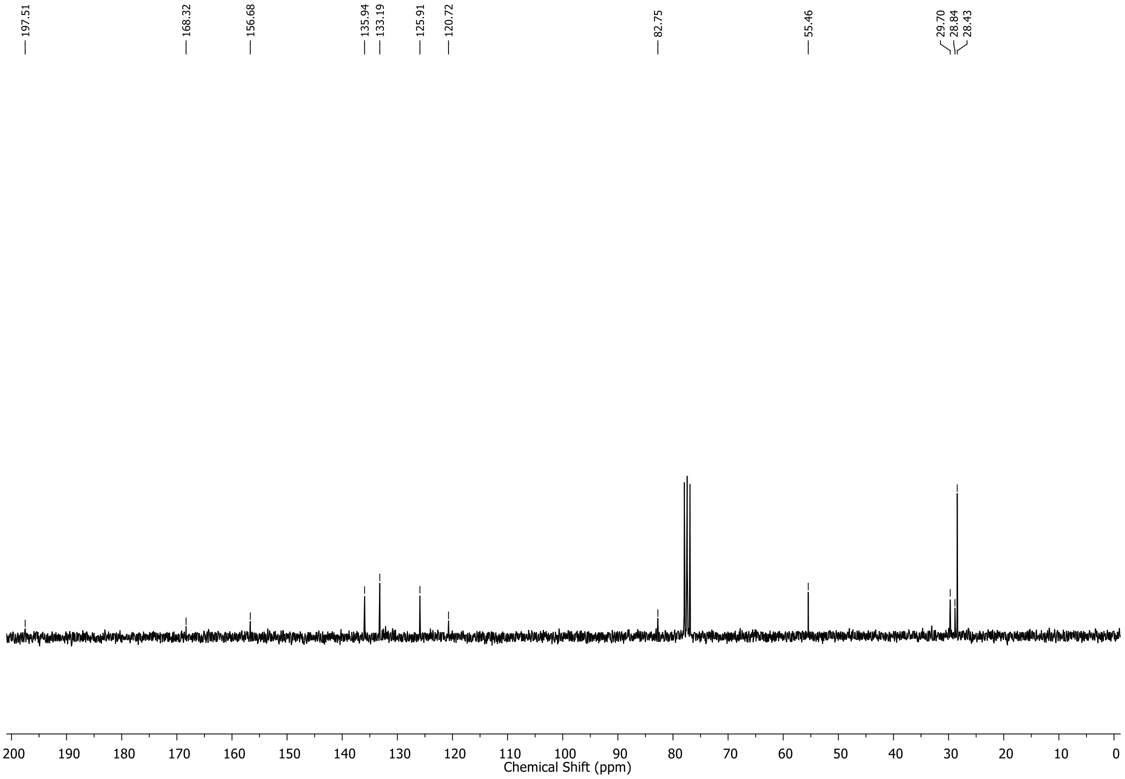

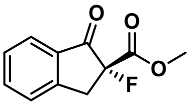

10a

^{1}H NMR (250 MHz, $CDCl_3$), HPLC and ^{19}F NMR(235 MHz, $CDCl_3$)

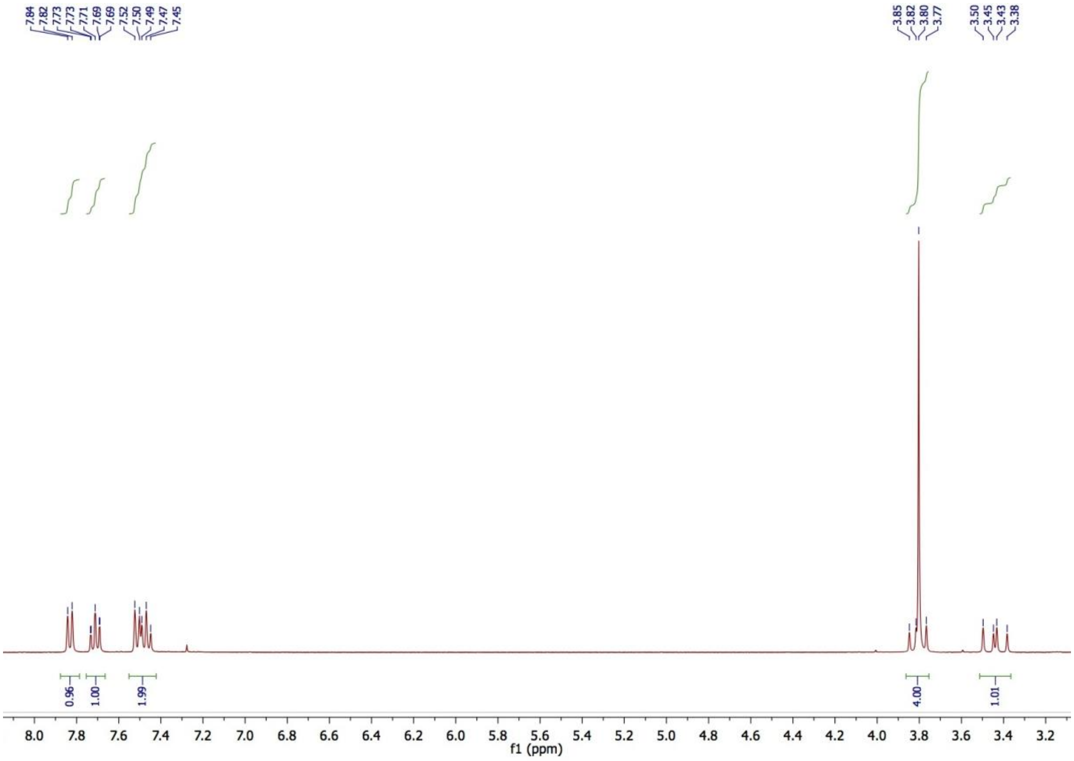

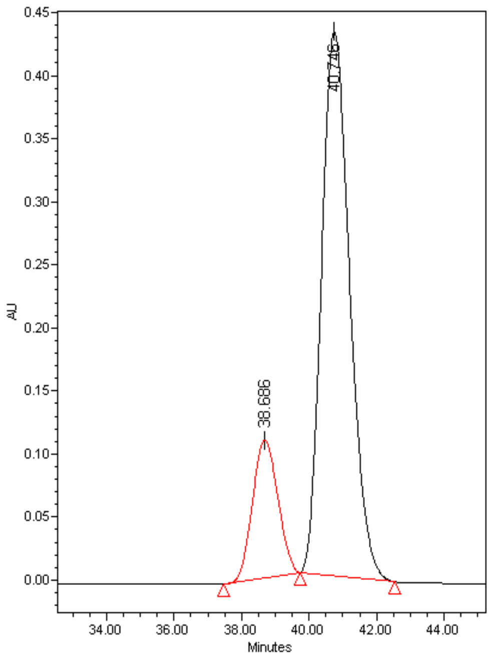

	Retention Time (min)	Purity1 Angle	Purity1 Threshold	PDA/FLR Match1 Spect. Name	PDA/FLR Match1 Angle	PDA/FLR Match1 Threshold	PDA/FLR Match1 Lib. Name	Area (µV*sec)	% Area
1	38.686							5689646	18.55
2	40.746							24989421	81.45

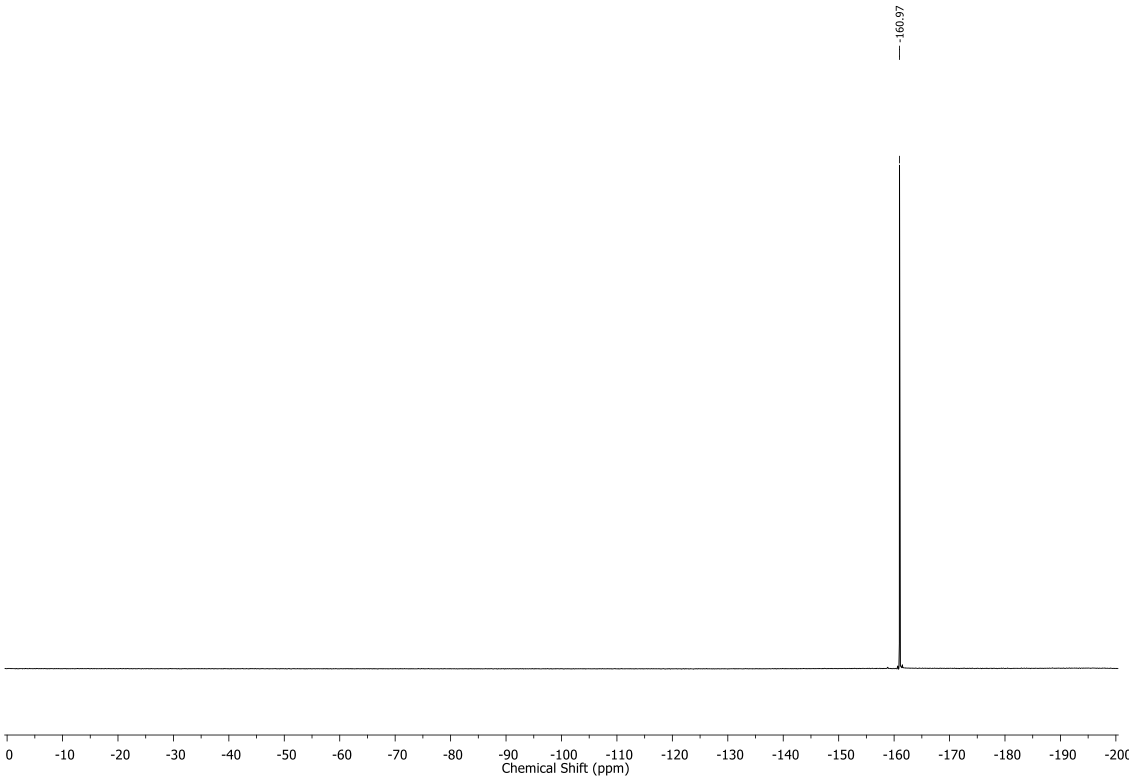

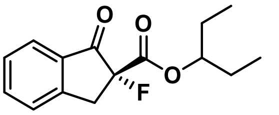

10b

1H NMR (250 Mz, $CDCl_3$)

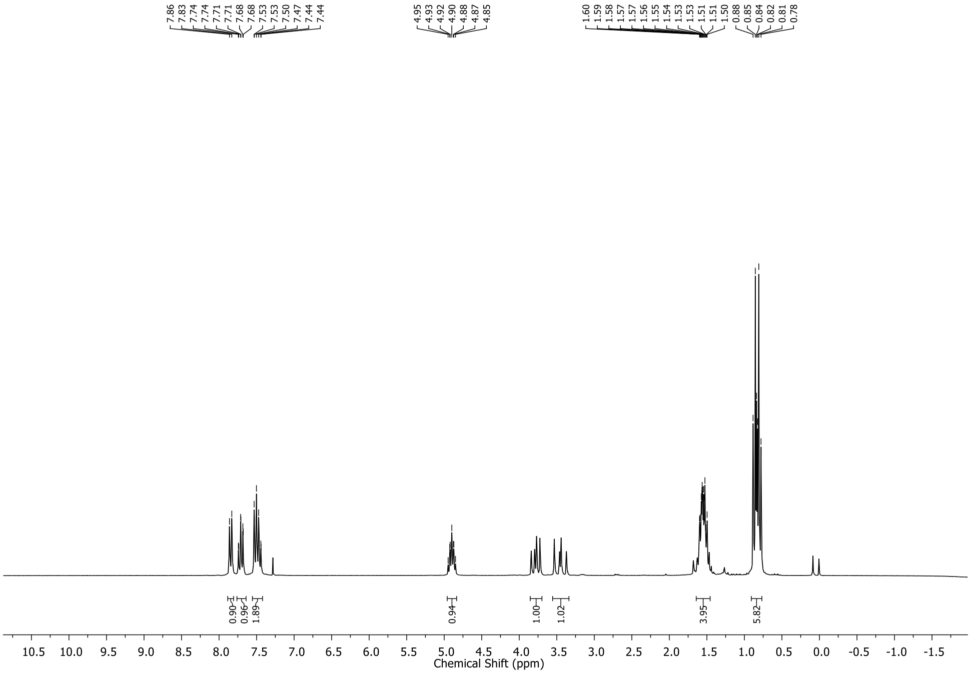

^{13}C NMR(91 MHz, $CDCl_3$)

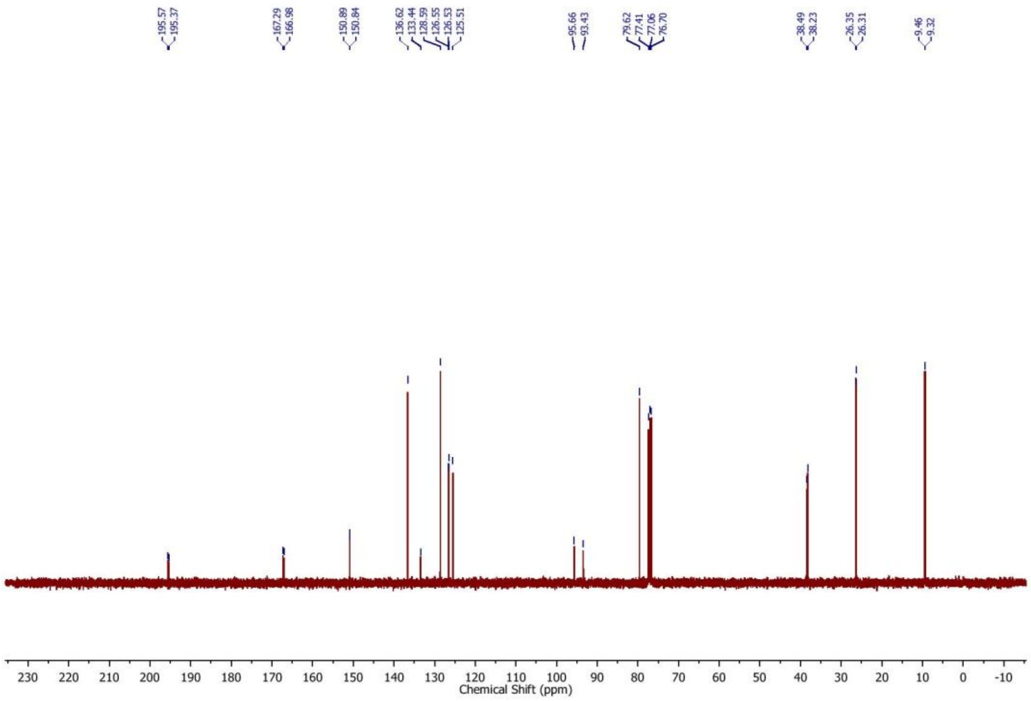

^{19}F NMR(235 MHz, $CDCl_3$)

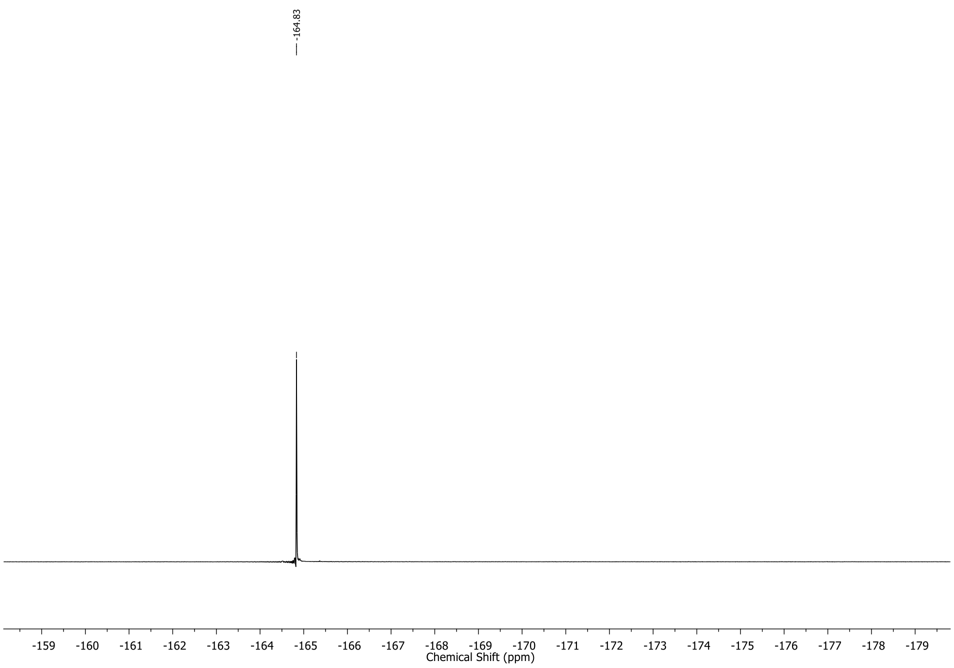

IR (ATR)

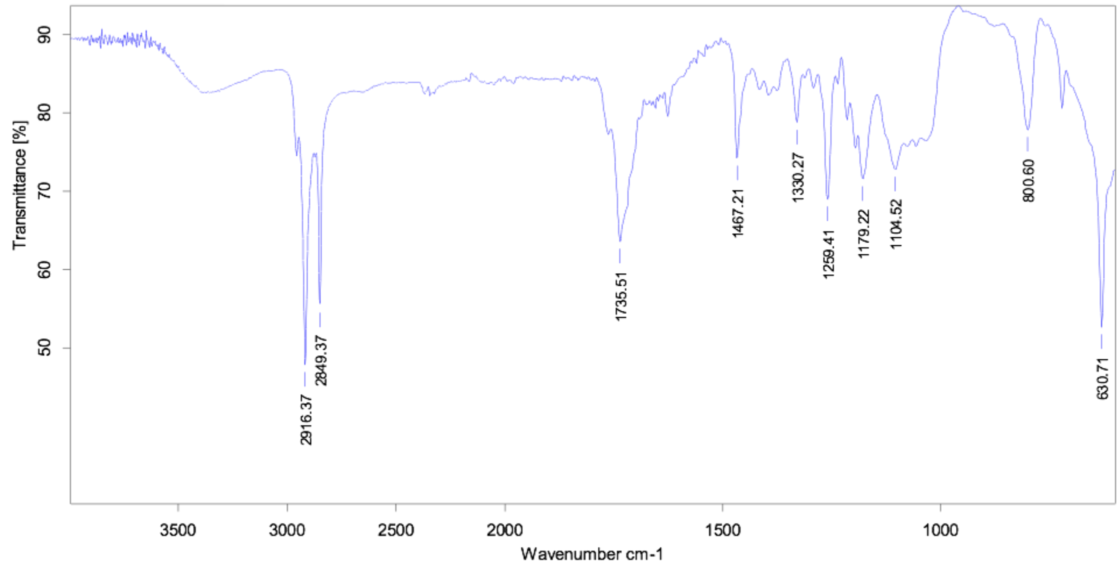

HPLC

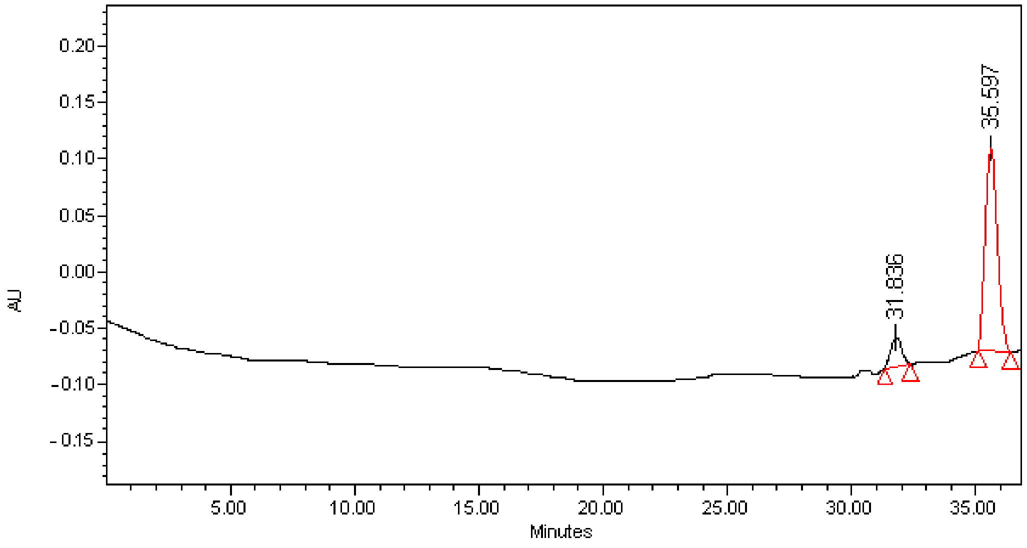

	Name	Retention Time (min)	Area (μV*sec)	% Area	Height (μV)	Int Type
1		31.836	7651	11.09	261	bb
2		35.597	61347	88.91	1806	bb

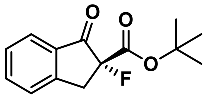

10c

^{1}H NMR (250 MHz, $CDCl_3$)

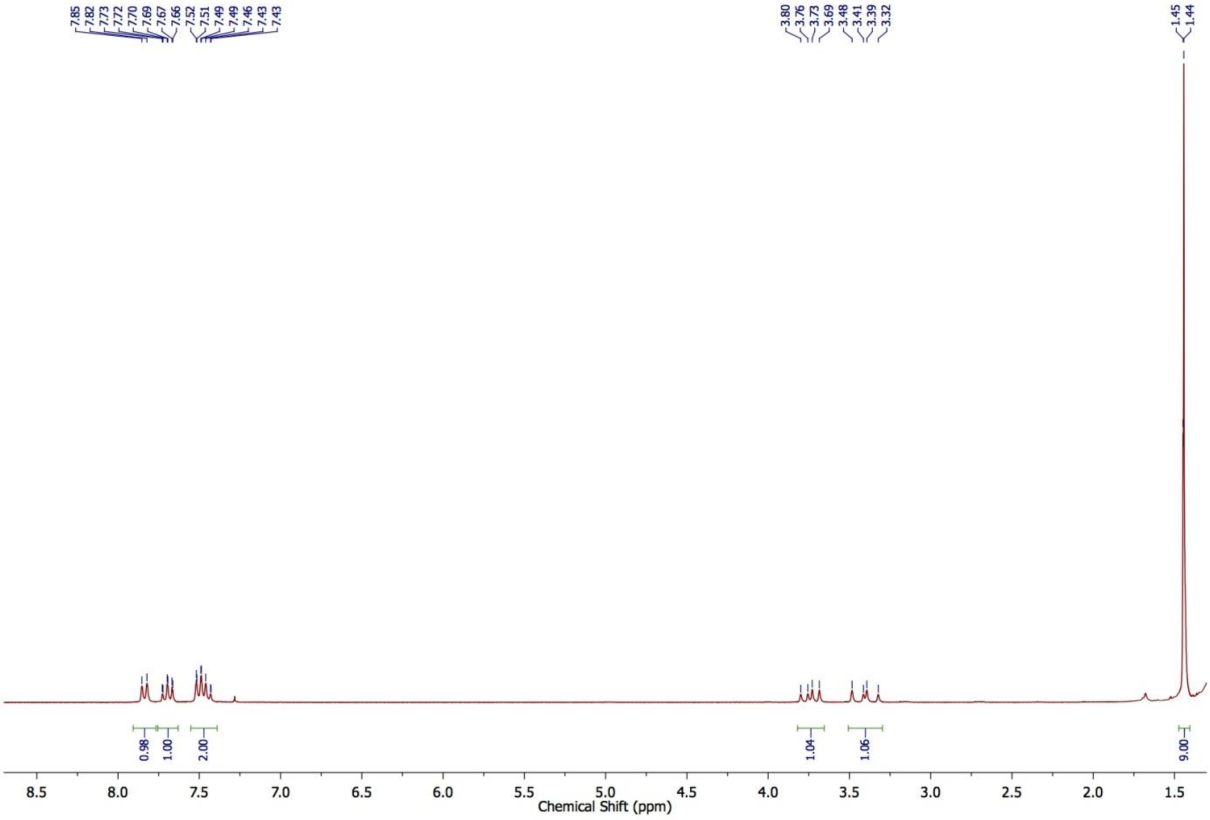

^{19}F NMR(235 MHz, $CDCl_3$)

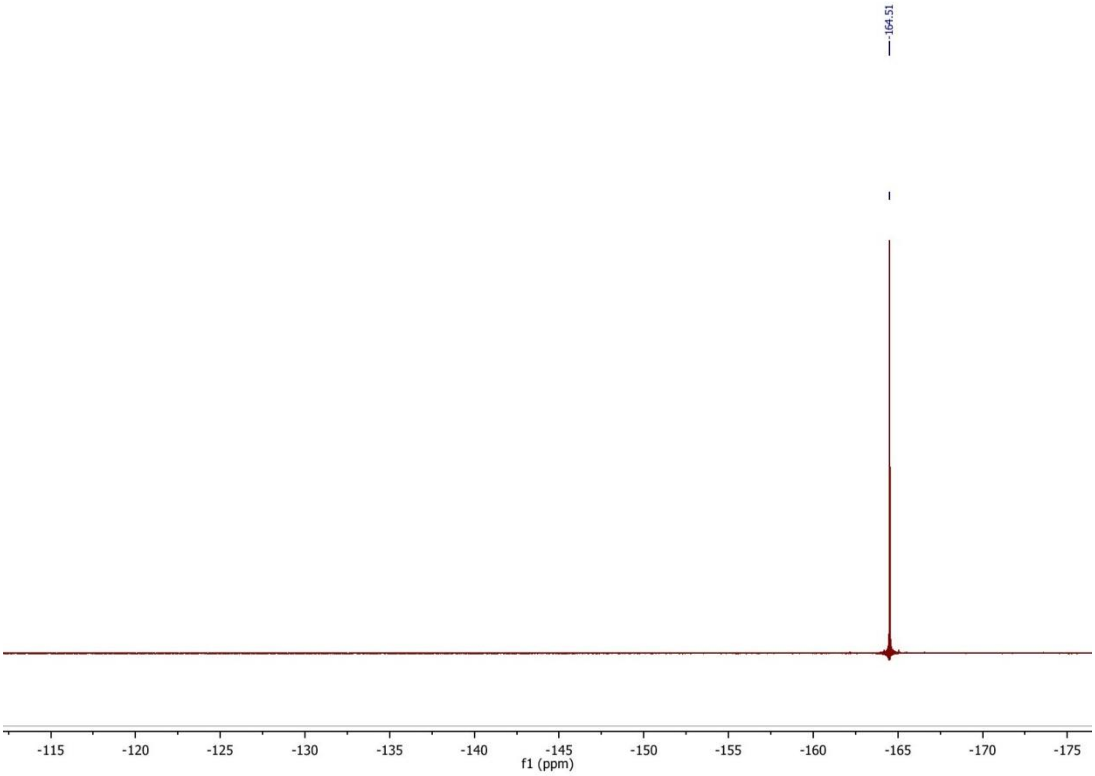

HPLC

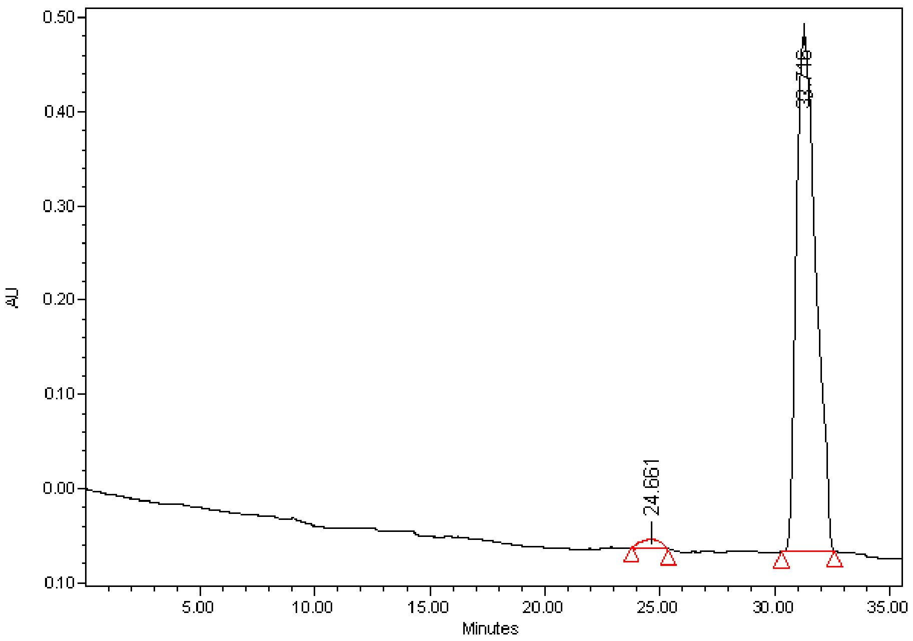

	Name	Retention Time (min)	Area (µV*sec)	% Area	Height (µV)	Int Type
1		24.661	97730	2.49	719	bb
2		33.761	3831190	97.51	24433	bb

CD (MeCN, 25ºC)

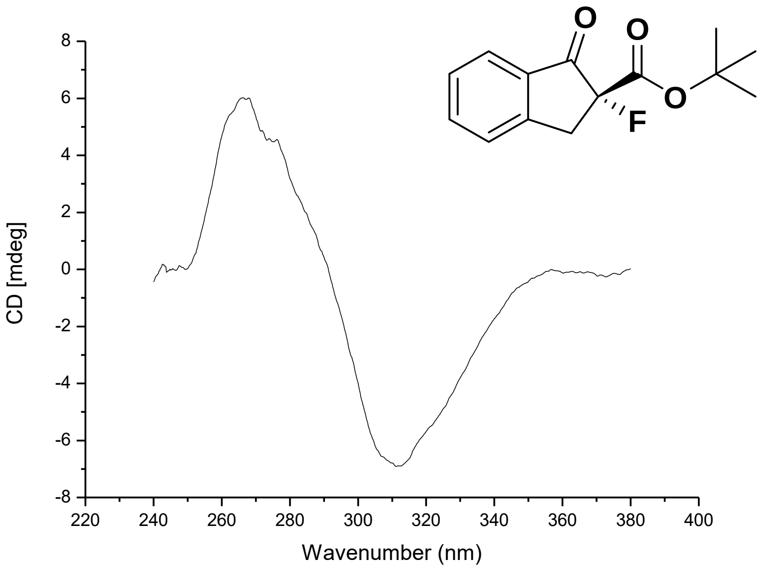

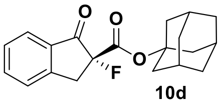

10d

^{1}H NMR (250 MHz, $CDCl_3$)

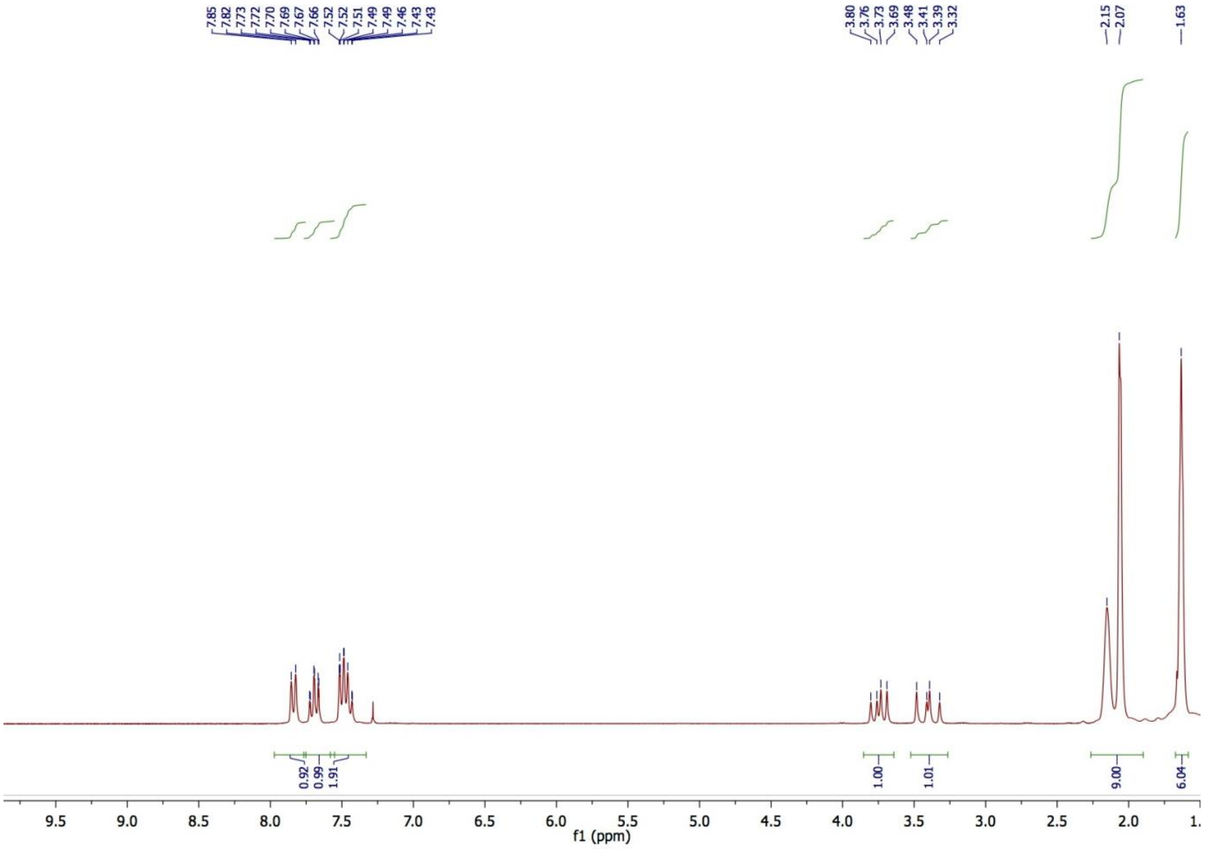

^{19}F NMR (235 MHz, $CDCl_3$)

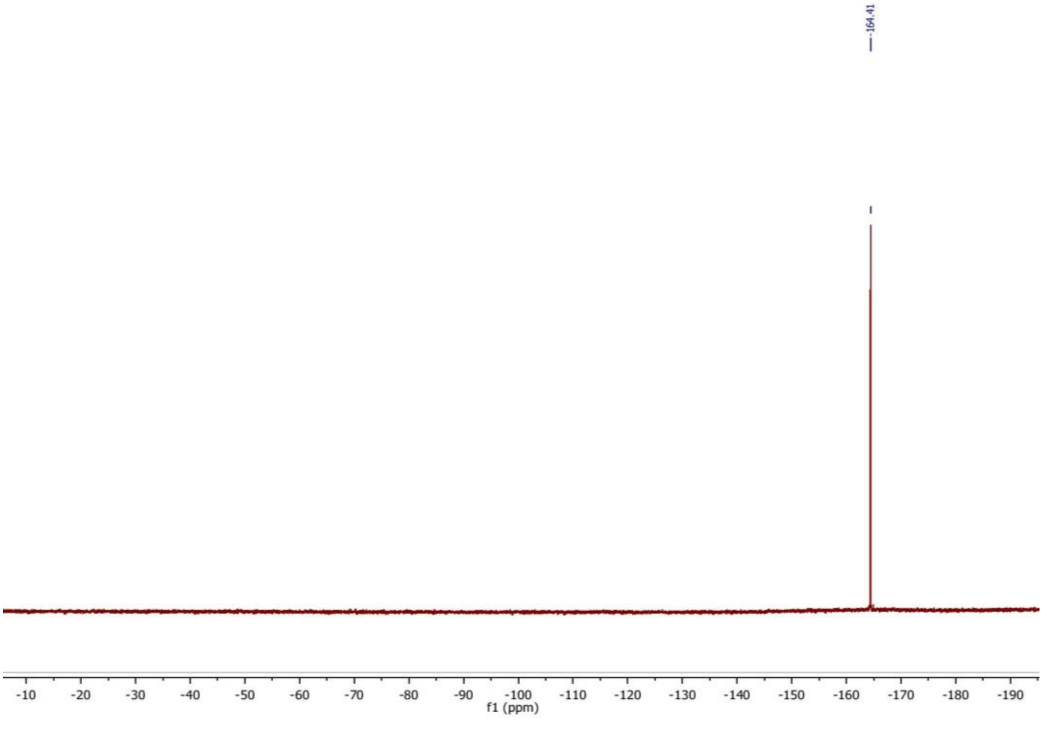

HPLC

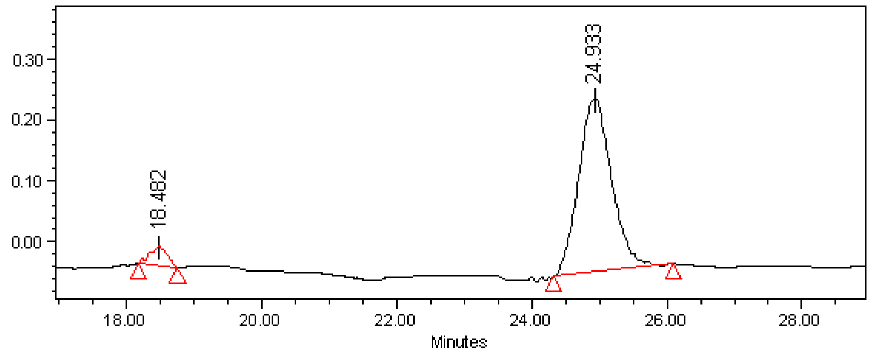

	Name	Retention Time (min)	Area (μV*sec)	% Area	Height (μV)	Int Type
1		18.482	2699	5.33	155	bb
2		24.933	47939	94.67	1411	bb

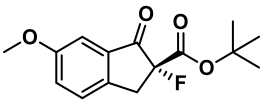

10e

^{1}H NMR (250 MHz, $CDCl_3$)

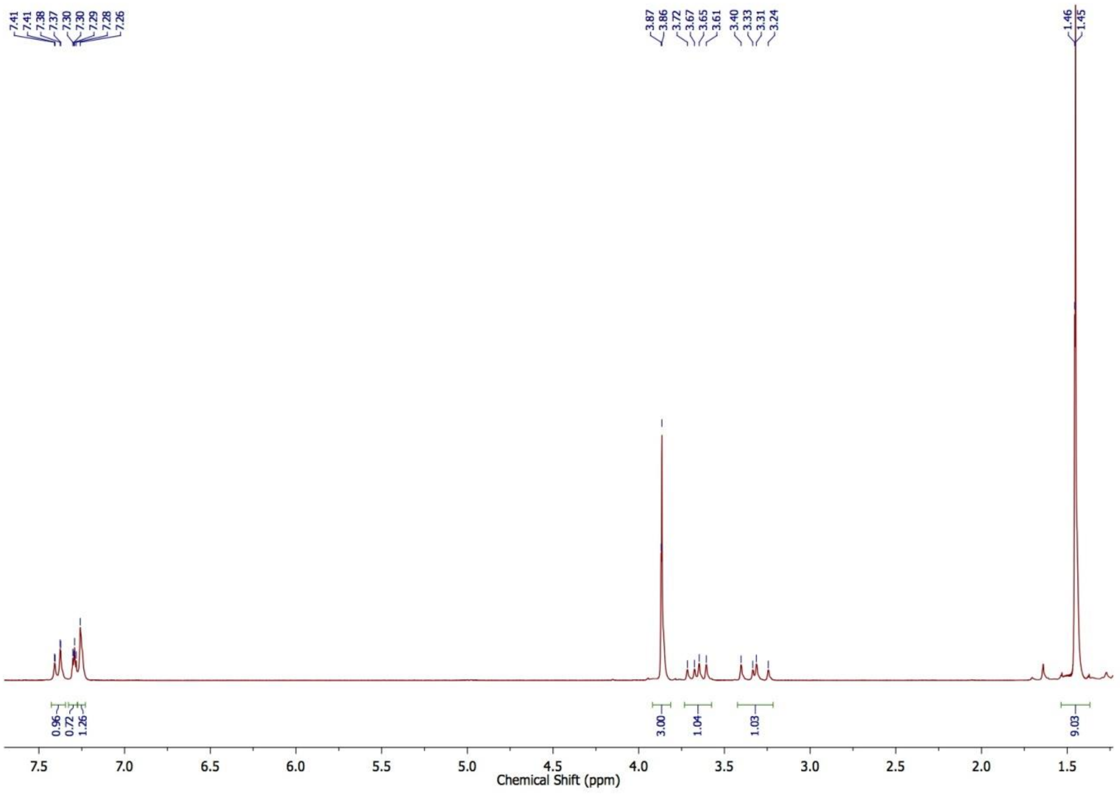

^{19}F NMR (235 MHz, $CDCl_3$)

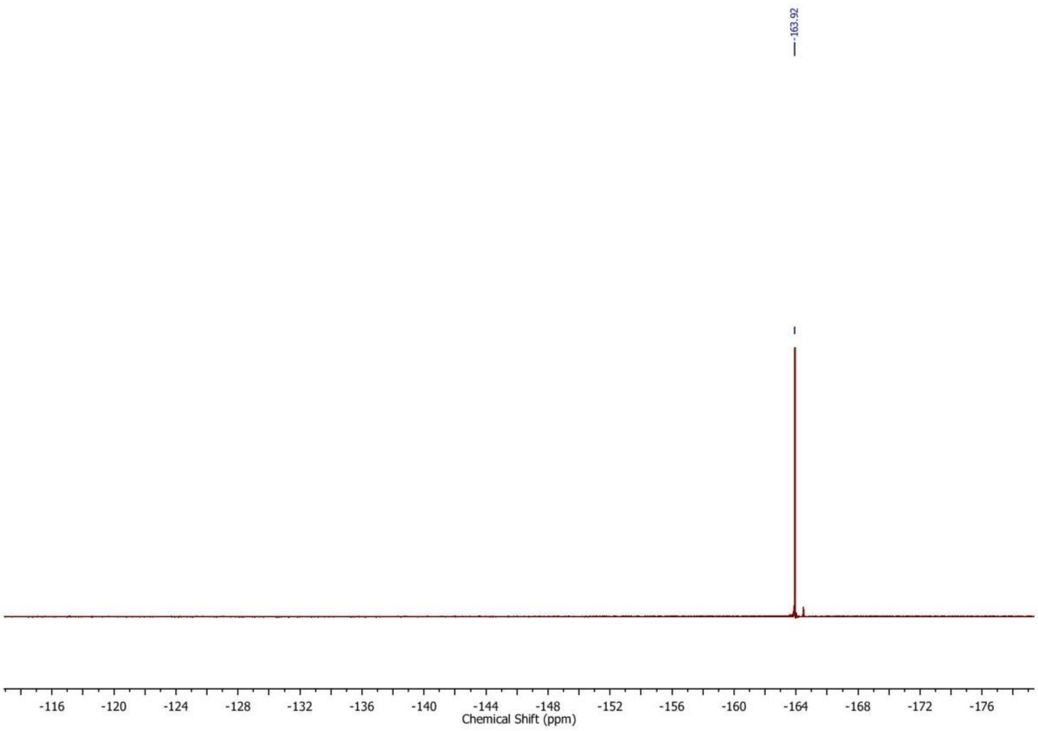

HPLC

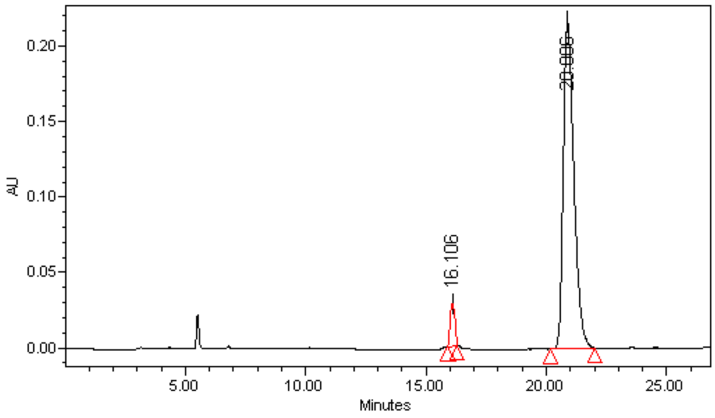

	Name	Retention Time (min)	Area (µV*sec)	% Area	Height (µV)	Int Type
1		16.106	399058	5.69	28434	bb
2		20.886	6619515	94.31	216558	bb

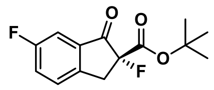

10f

^{1}H NMR (250 MHz, $CDCl_3$)

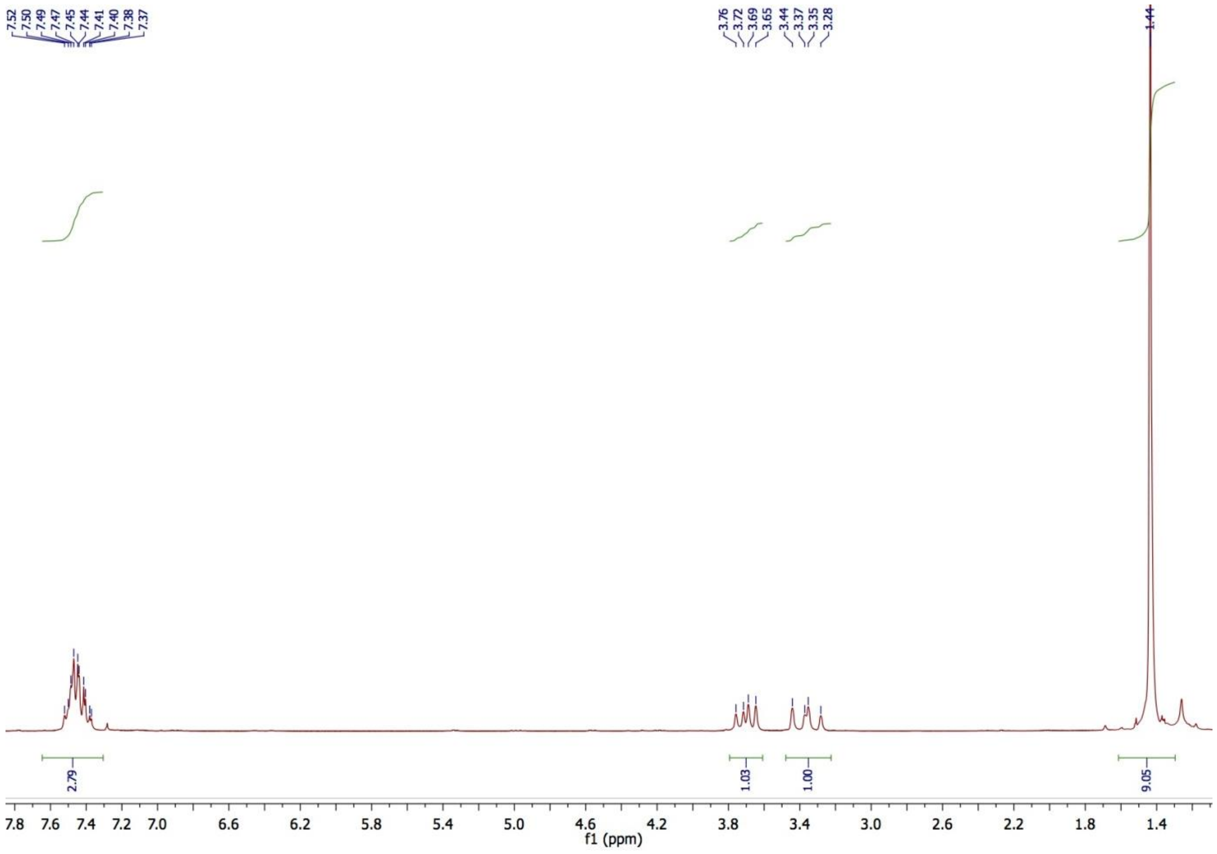

^{19}F NMR (235 MHz, $CDCl_3$)

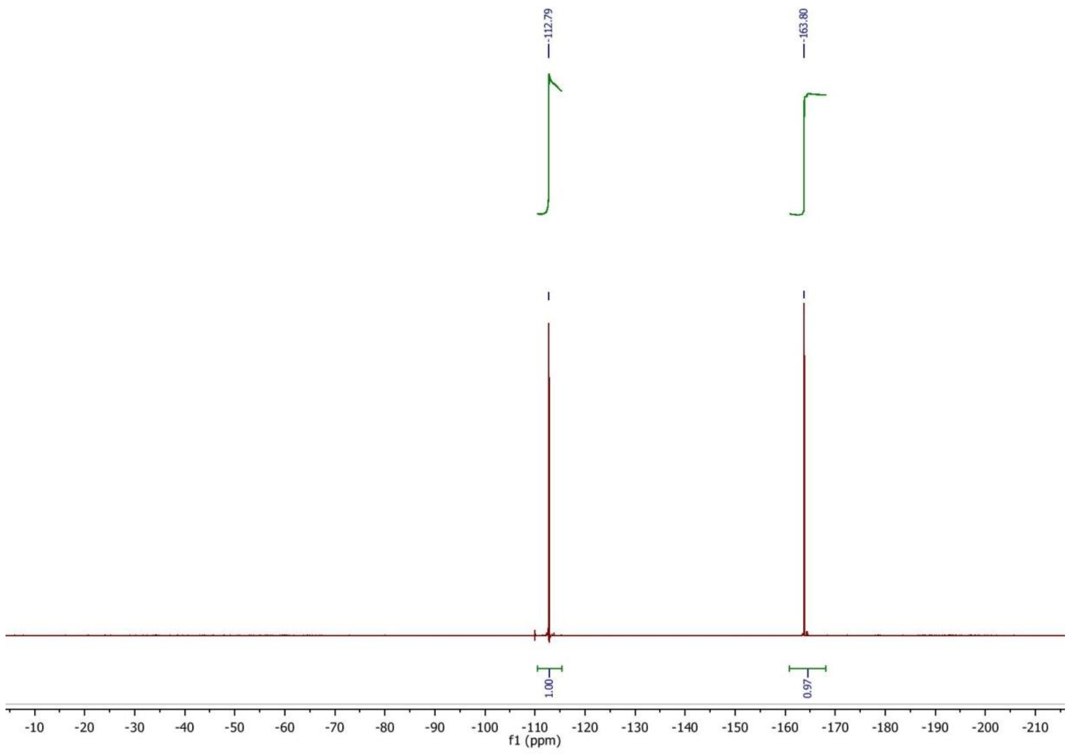

HPLC

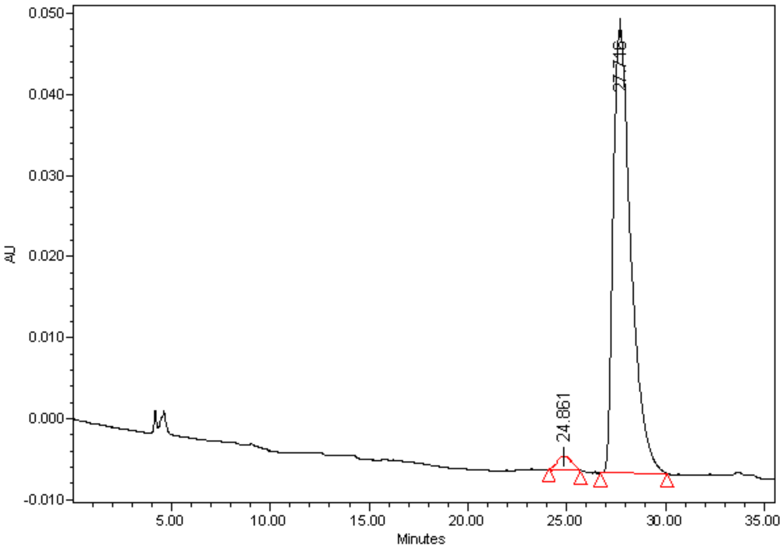

	Name	Retention Time (min)	Area (µV*sec)	% Area	Height (µV)	Int Type
1		24.425	129898	3.65	-3094	bb
2		27.861	3429908	96.35	54929	bb

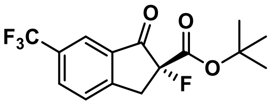

10g

^{1}H NMR (360 MHz, $CDCl_3$)

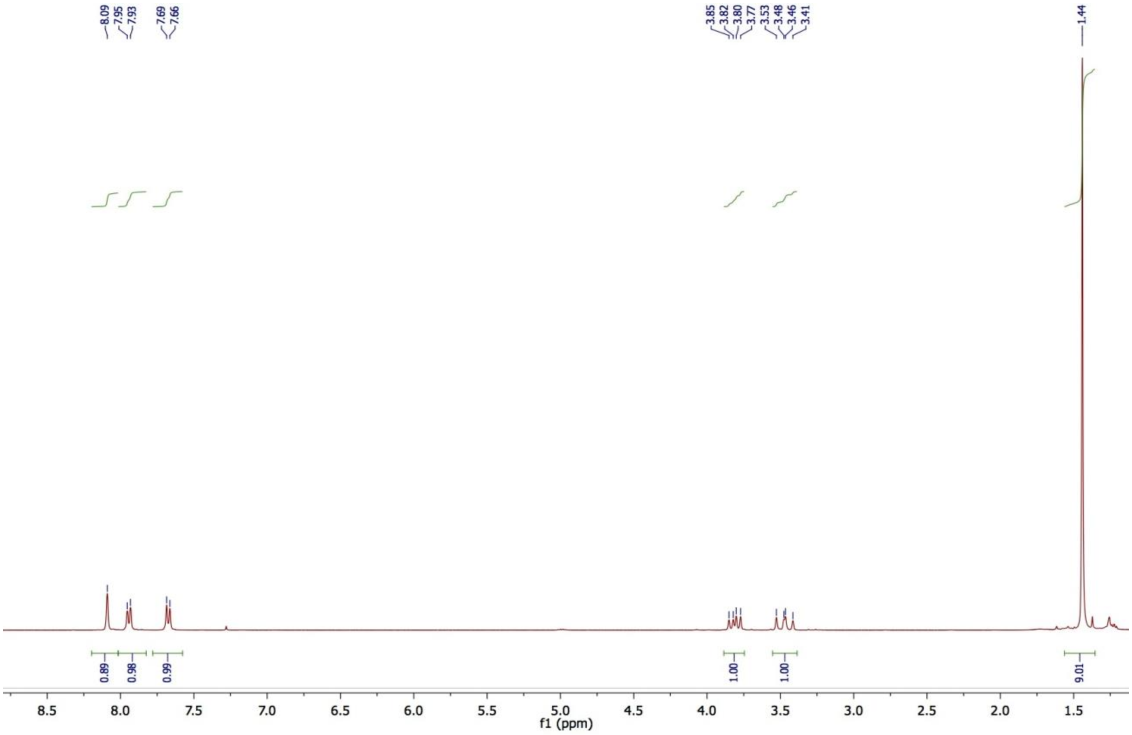

^{13}C NMR (91 MHz, $CDCl_3$)

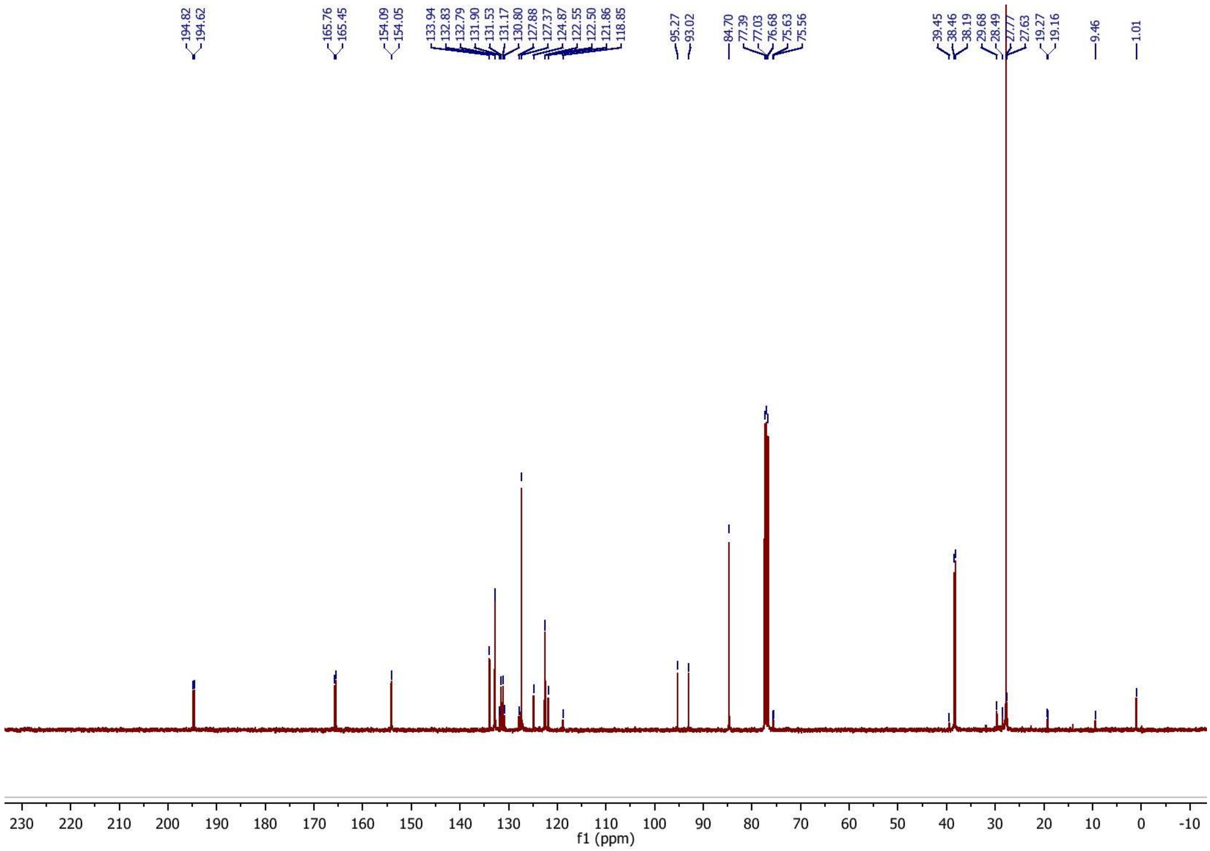

^{19}F NMR (235 MHz, $CDCl_3$)

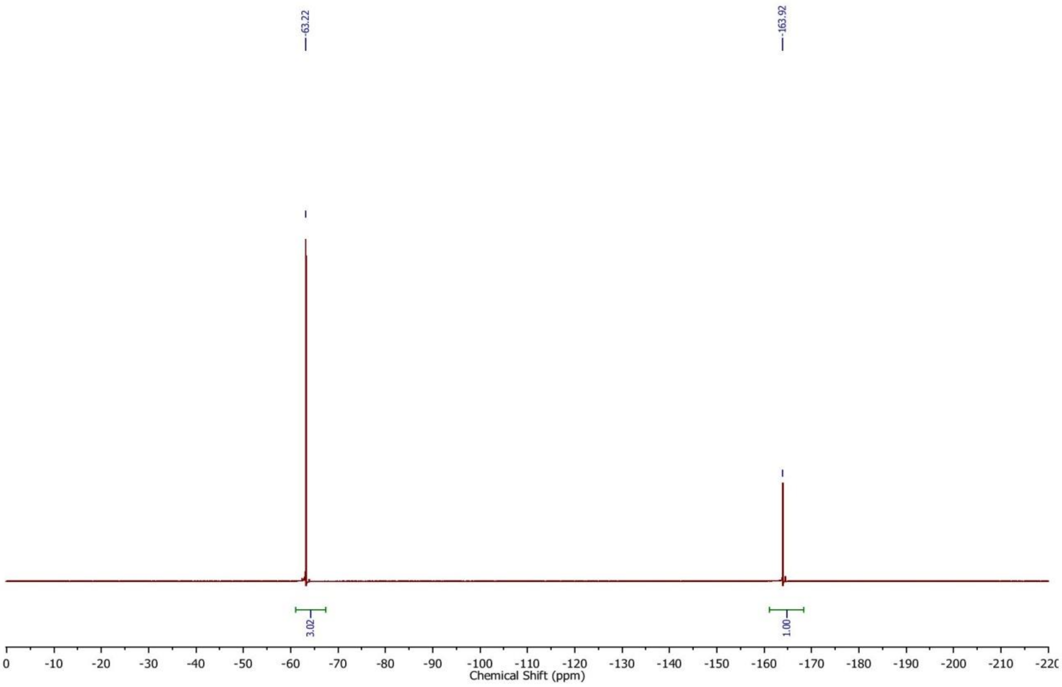

IR

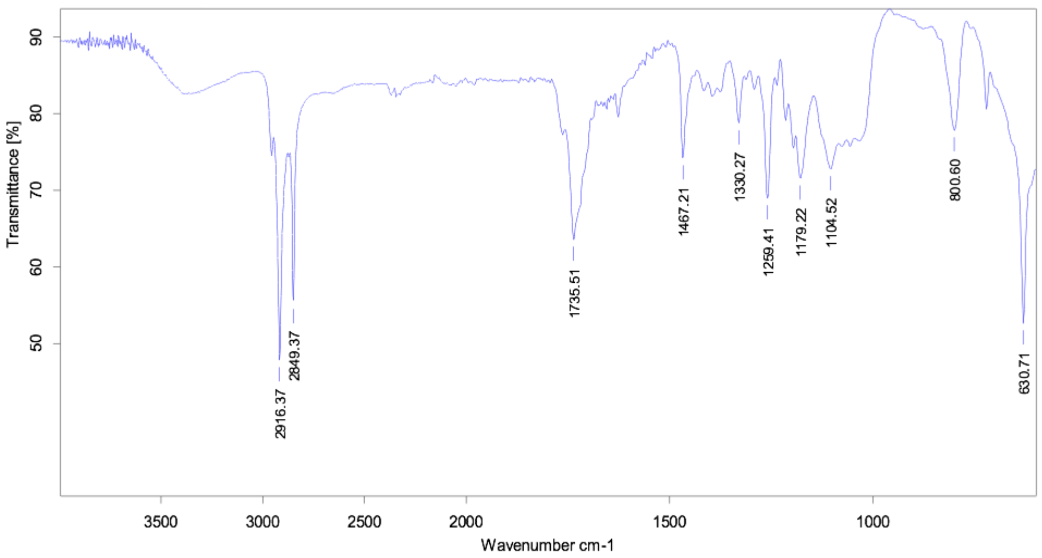

HPLC

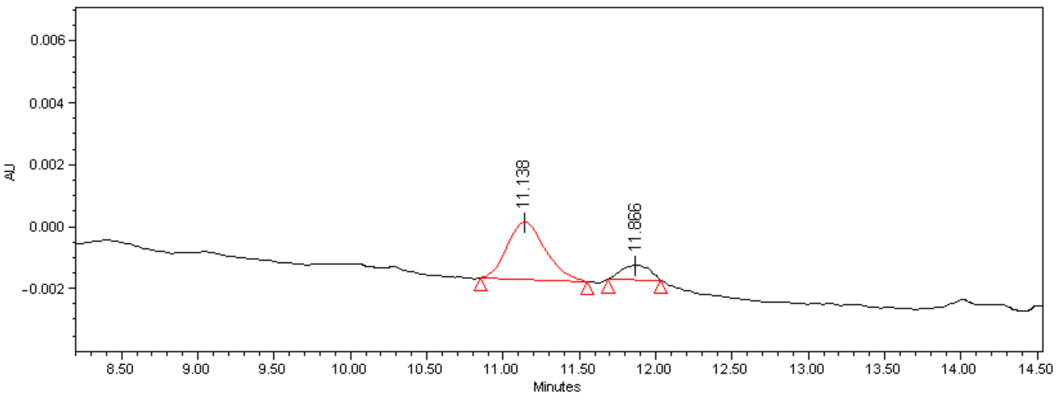

	Name	Retention Time (min)	Area (µV*sec)	% Area	Height (µV)	Int Type
1		11.138	32332	91.40	1850	bb
2		11.866	3040	8.60	281	bb

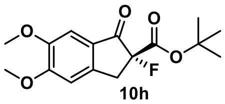

10h

^{1}H NMR (360 MHz, $CDCl_3$)

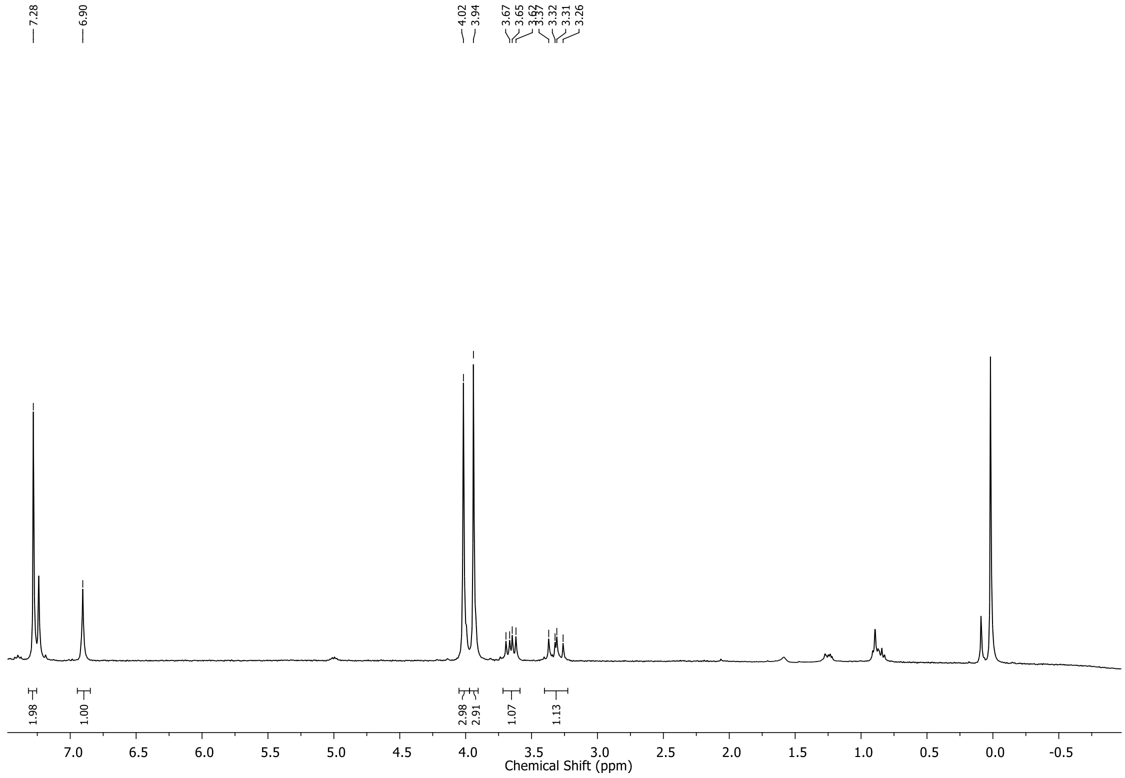

^{19}F NMR (235 MHz, $CDCl_3$)

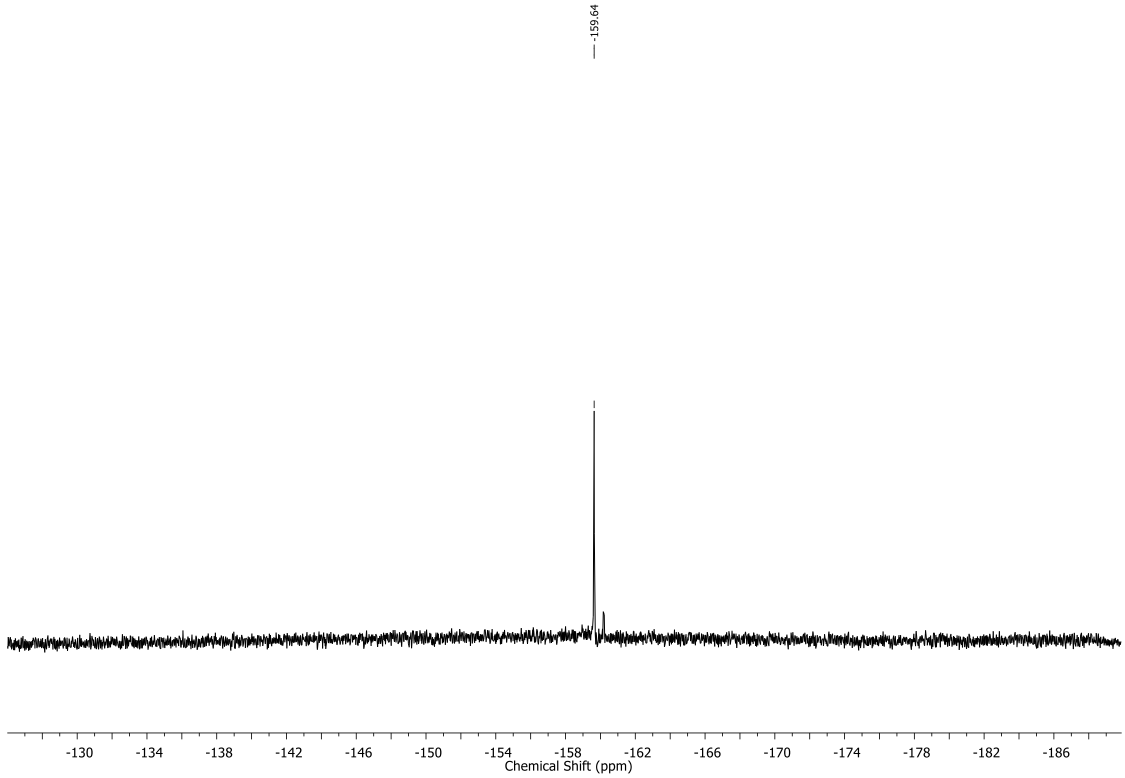

HPLC

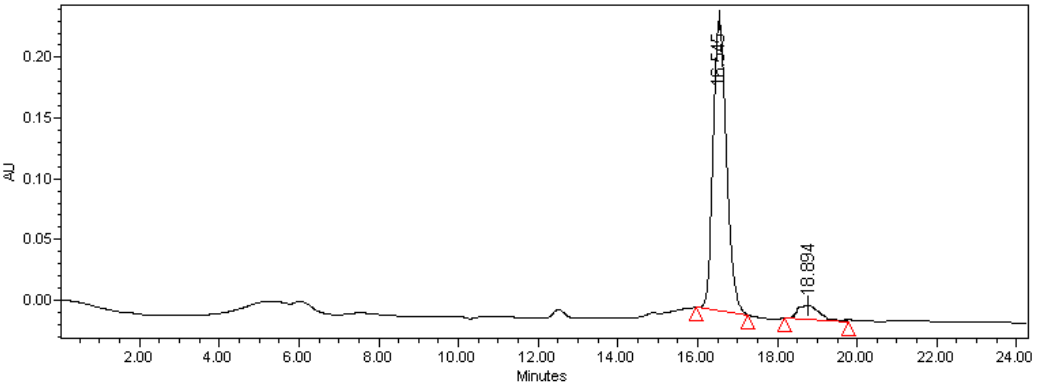

	Name	Retention Time (min)	Area (µV*sec)	% Area	Height (µV)	Int Type
1		16.545	4132861	95.29	179762	bb
2		18.894	87838	4.71	8601	bb

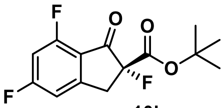

10i

^{1}H NMR (400 MHz, $CDCl_3$)

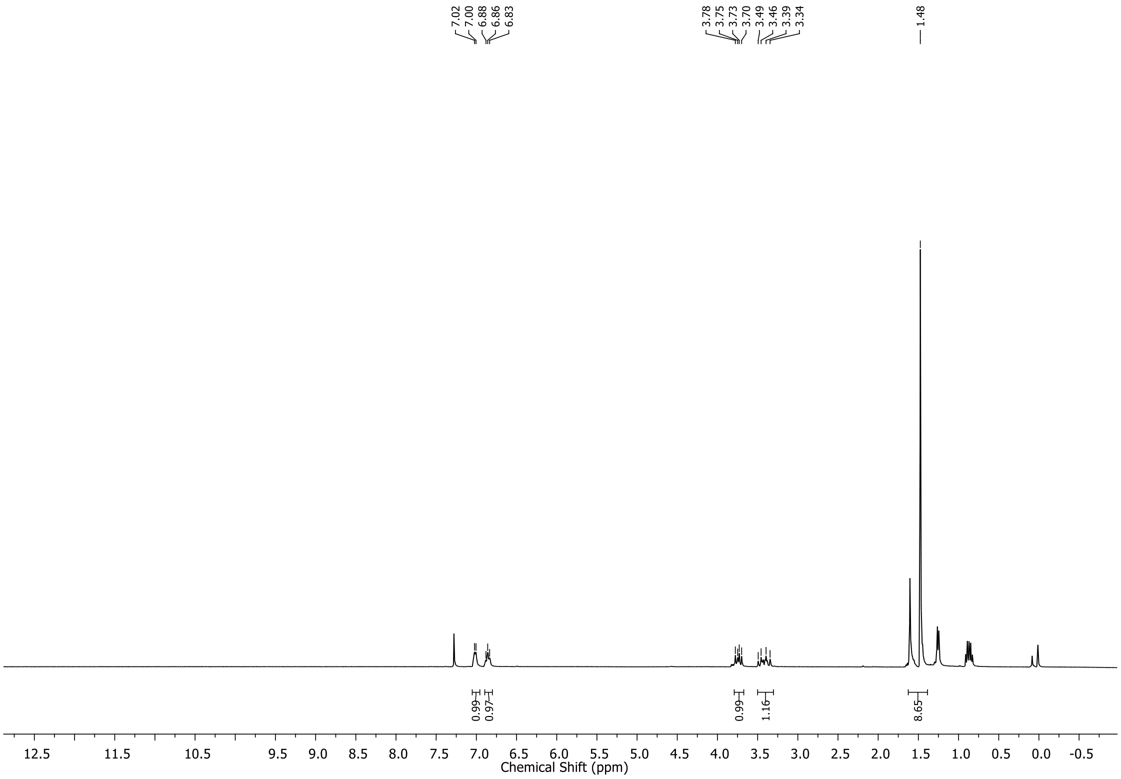

^{13}C NMR (101 MHz, CDCl$_3$)

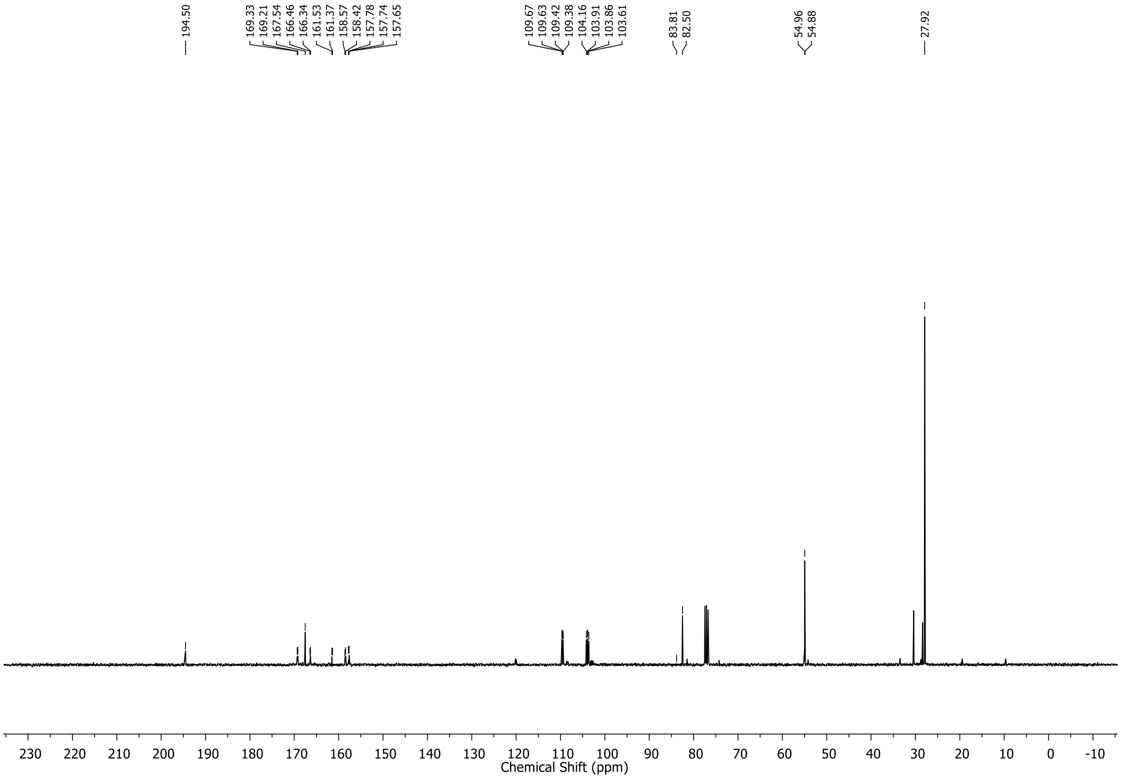

^{19}F NMR (376 MHz, CDCl$_3$)

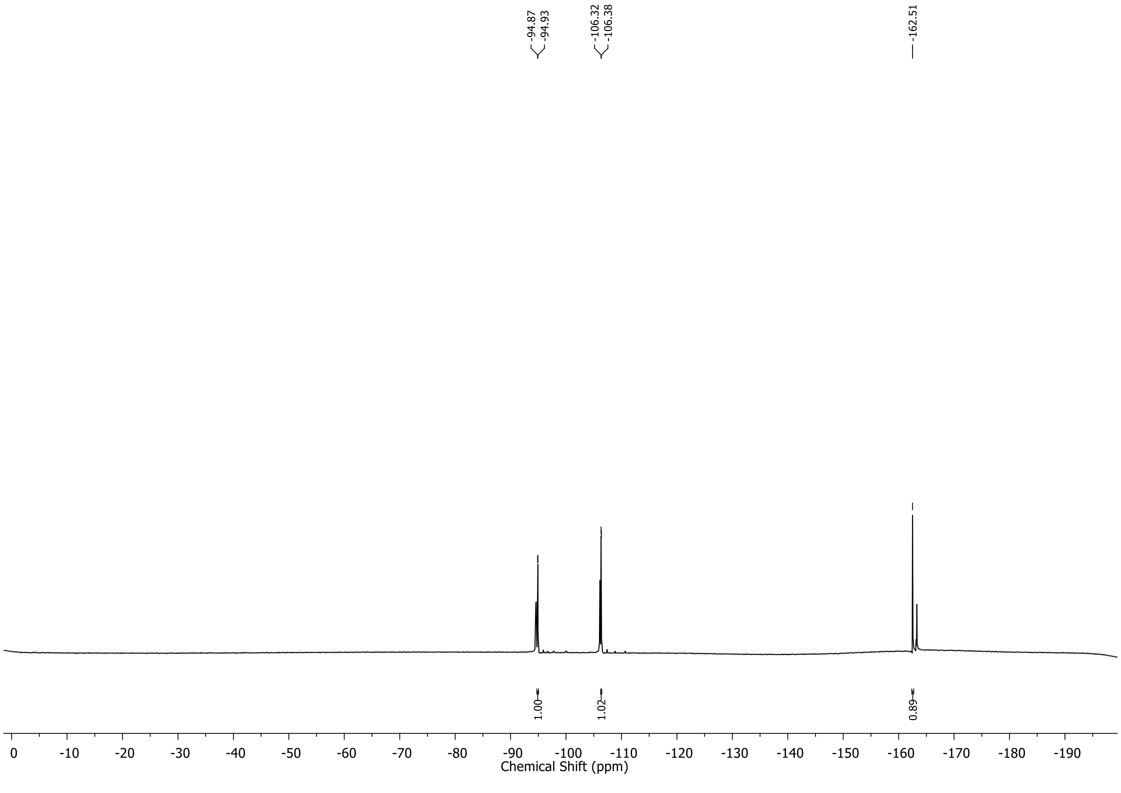

HPLC

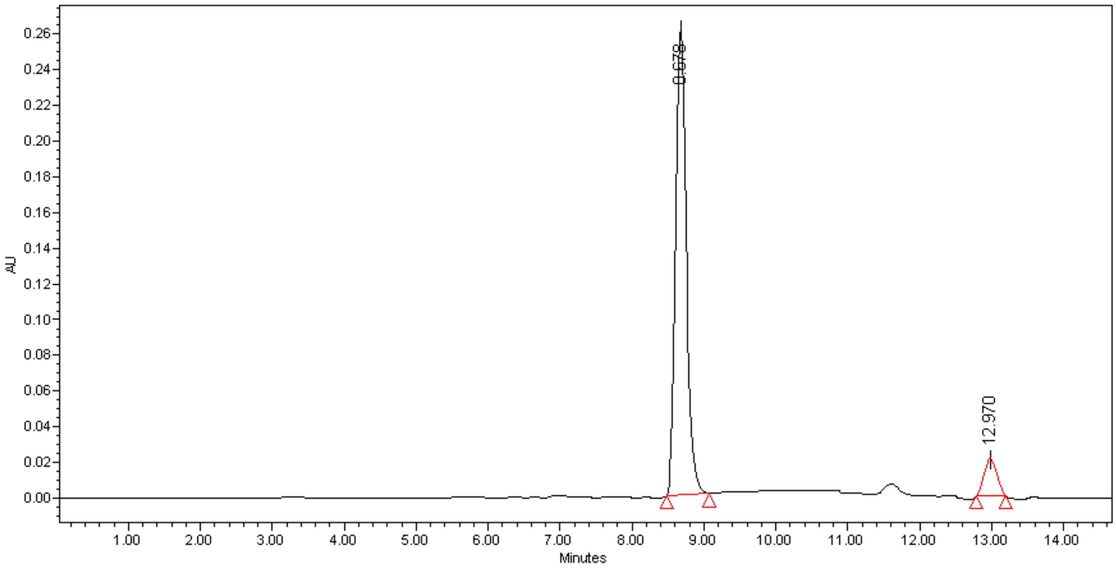

	Name	Retention Time (min)	Area (µV*sec)	% Area	Height (µV)	Int Type
1		8.678	2677746	91.18	261119	bb
2		12.970	259007	8.82	20719	bb

CD (MeCN, 25ºC)

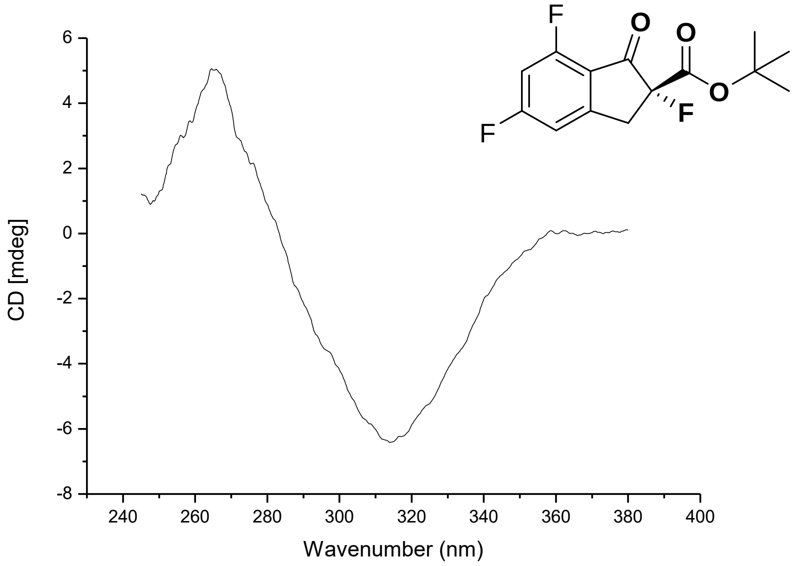

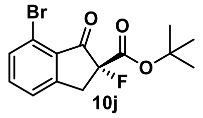

10j

^{1}H NMR (360 MHz, $CDCl_3$)

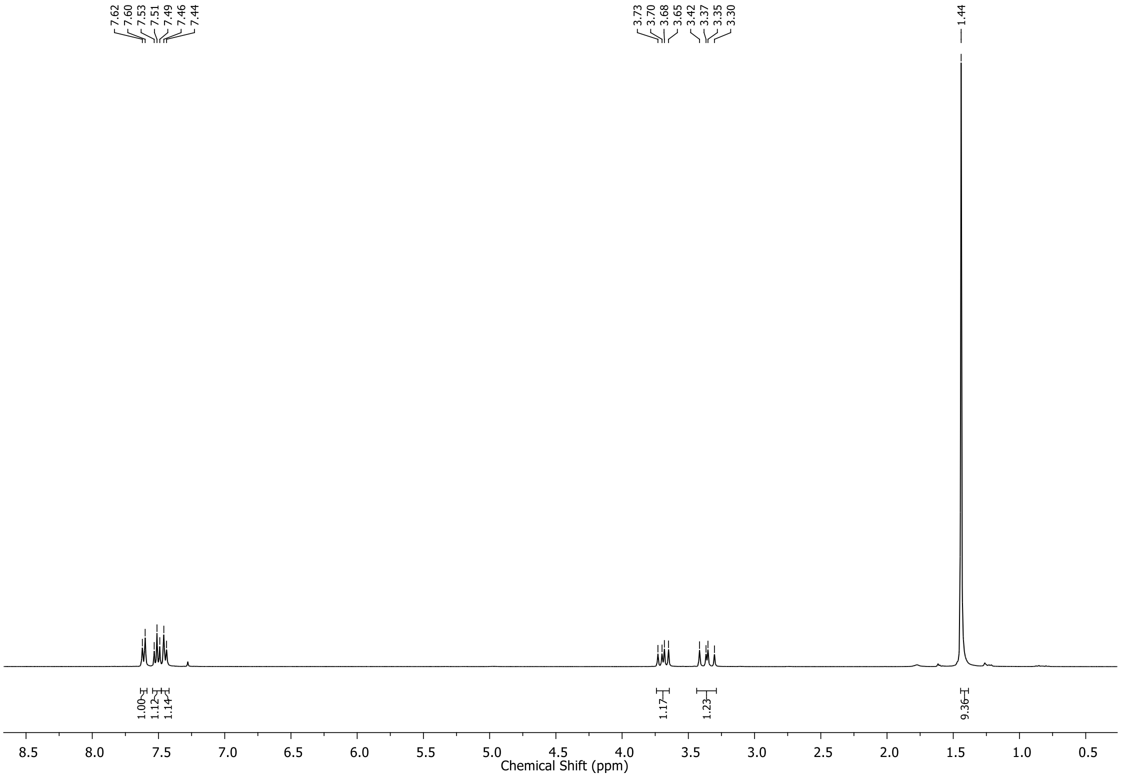

^{13}C NMR (91 MHz, $CDCl_3$)

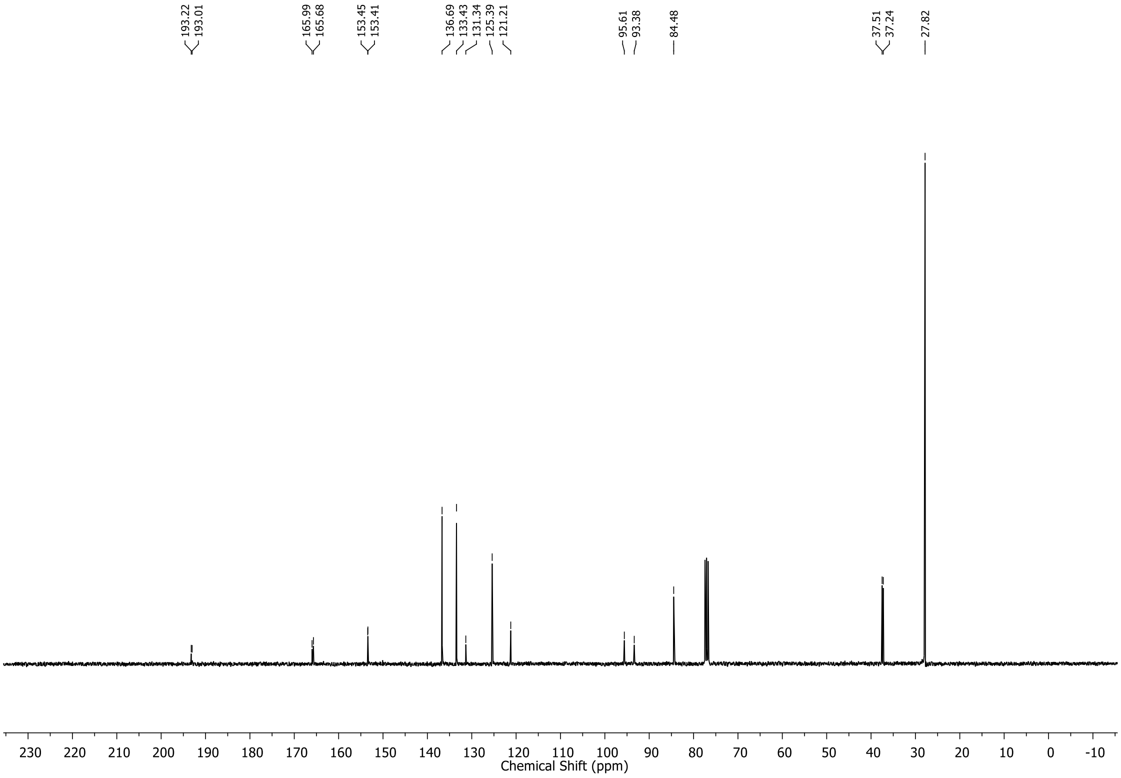

^{19}F NMR (235 MHz, $CDCl_3$)

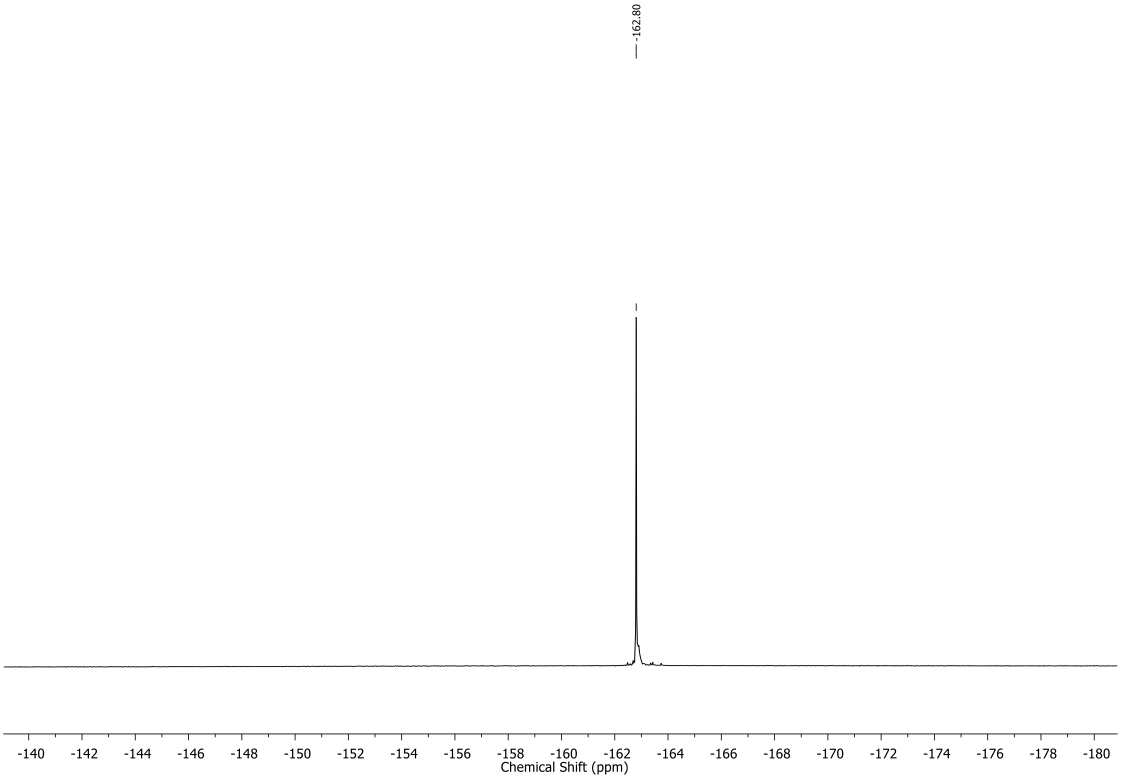

HPLC

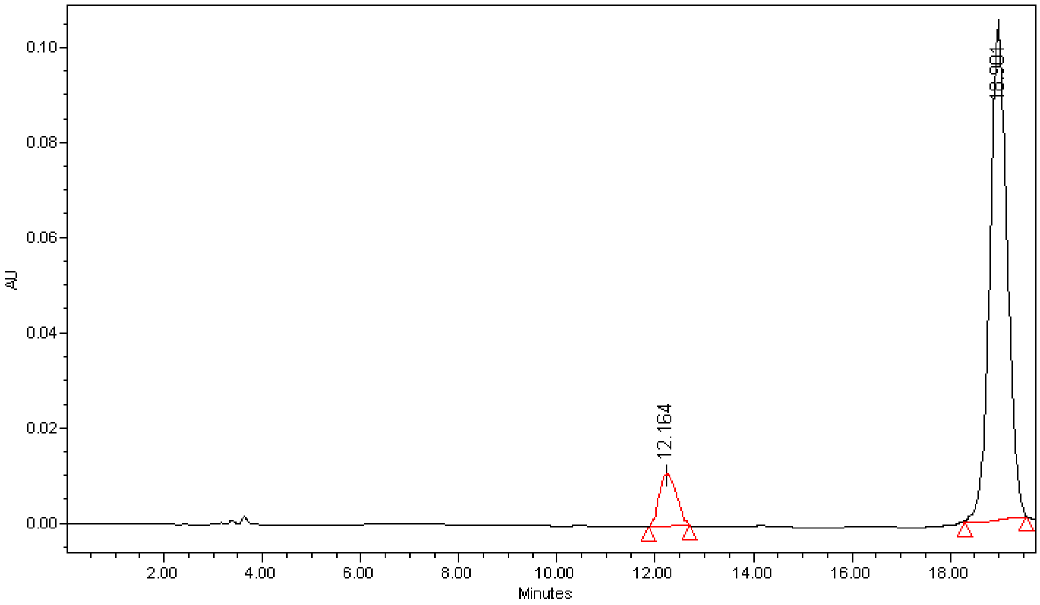

	Name	Retention Time (min)	Area (µV*sec)	% Area	Height (µV)	Int Type
1		18.113	184731	7.45	8991	bb
2		12.164	2293772	92.55	97831	bb

CD (MeCN, 25ºC)

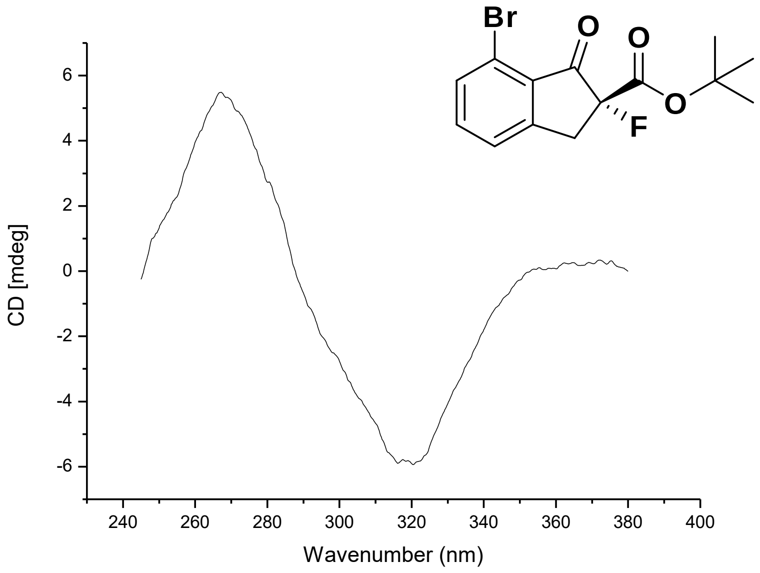

IR (ATR)

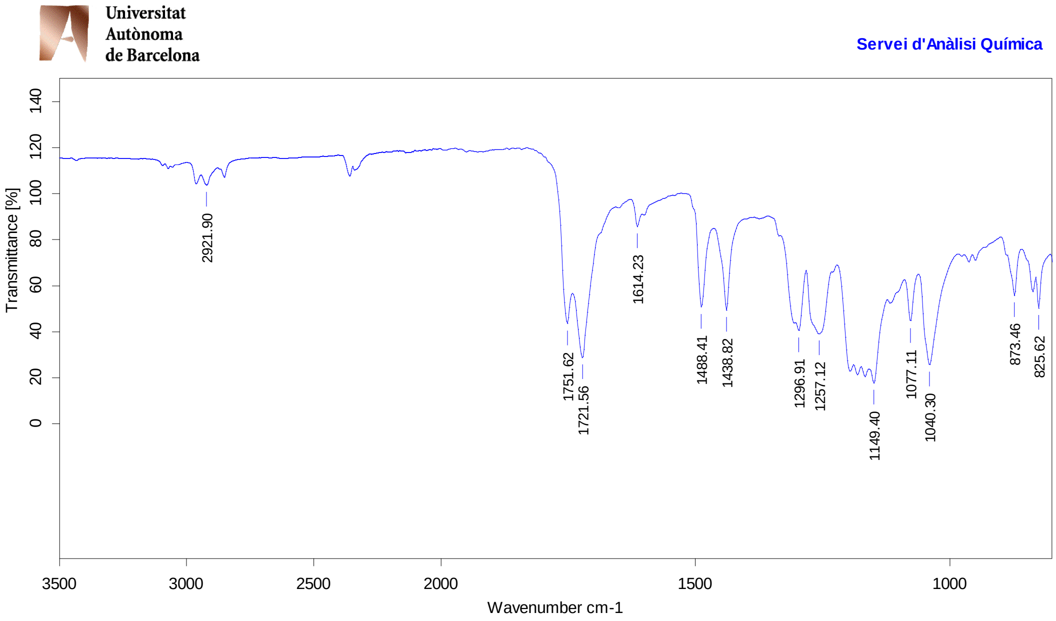

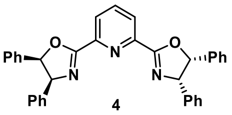

4

^{1}H NMR (250 MHz, $CDCl_3$)

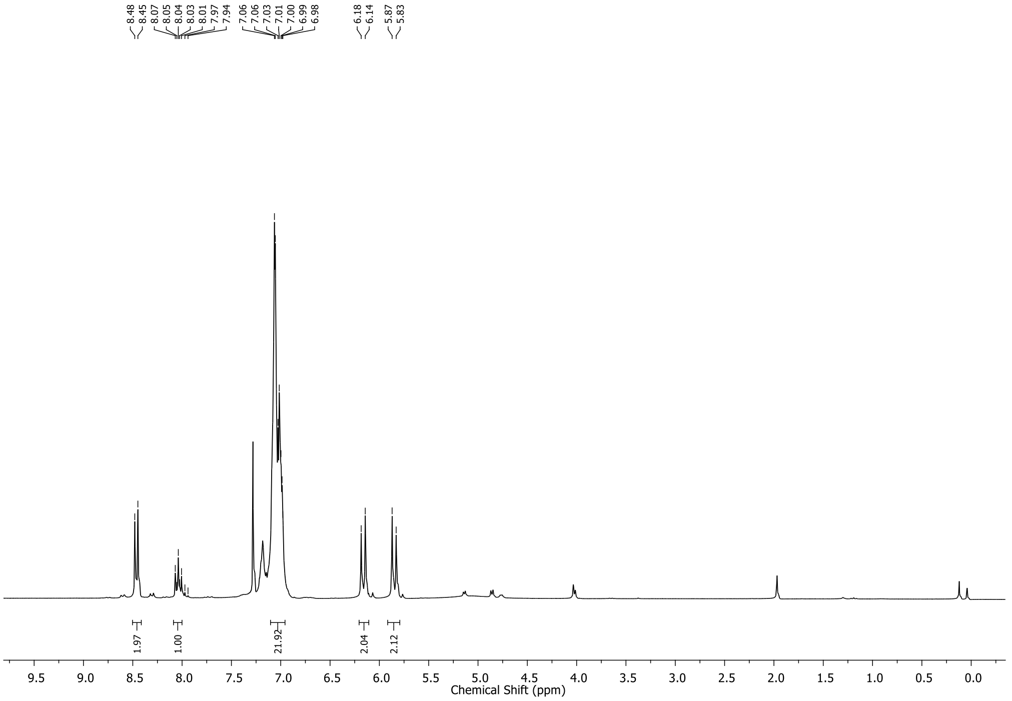

^{13}C NMR (63 MHz, $CDCl_3$)

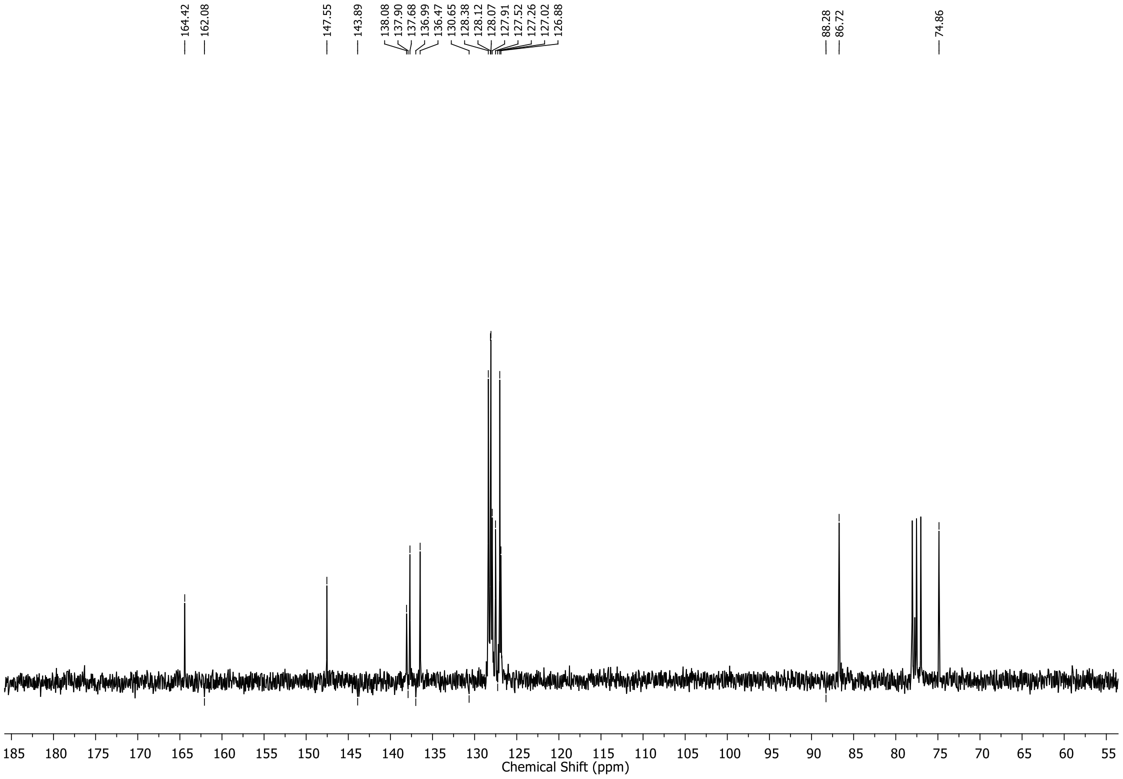

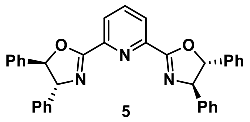

5

^{1}H NMR (250 MHz, $CDCl_3$)

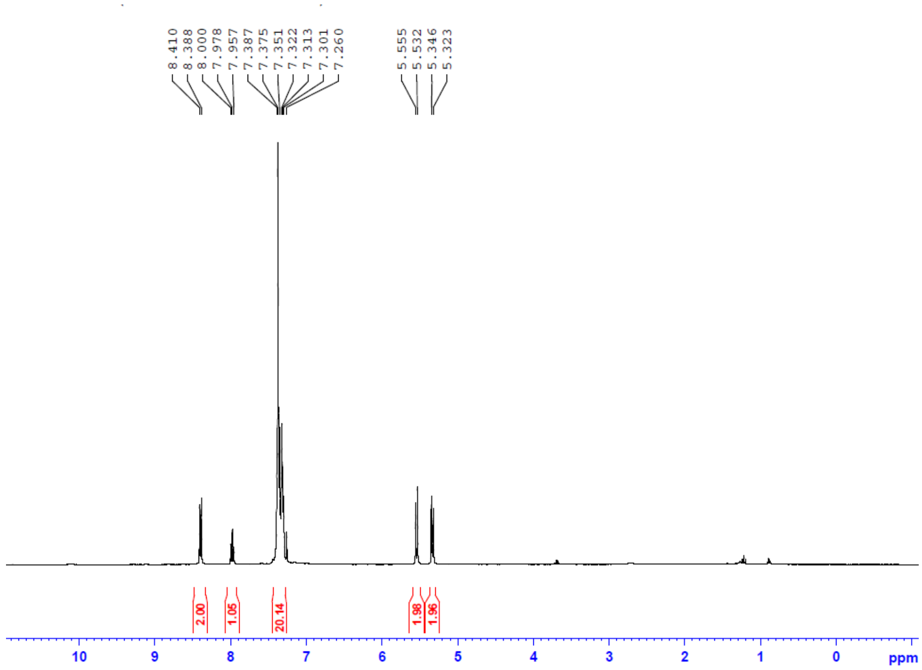

^{13}C NMR (91 MHz, $CDCl_3$)

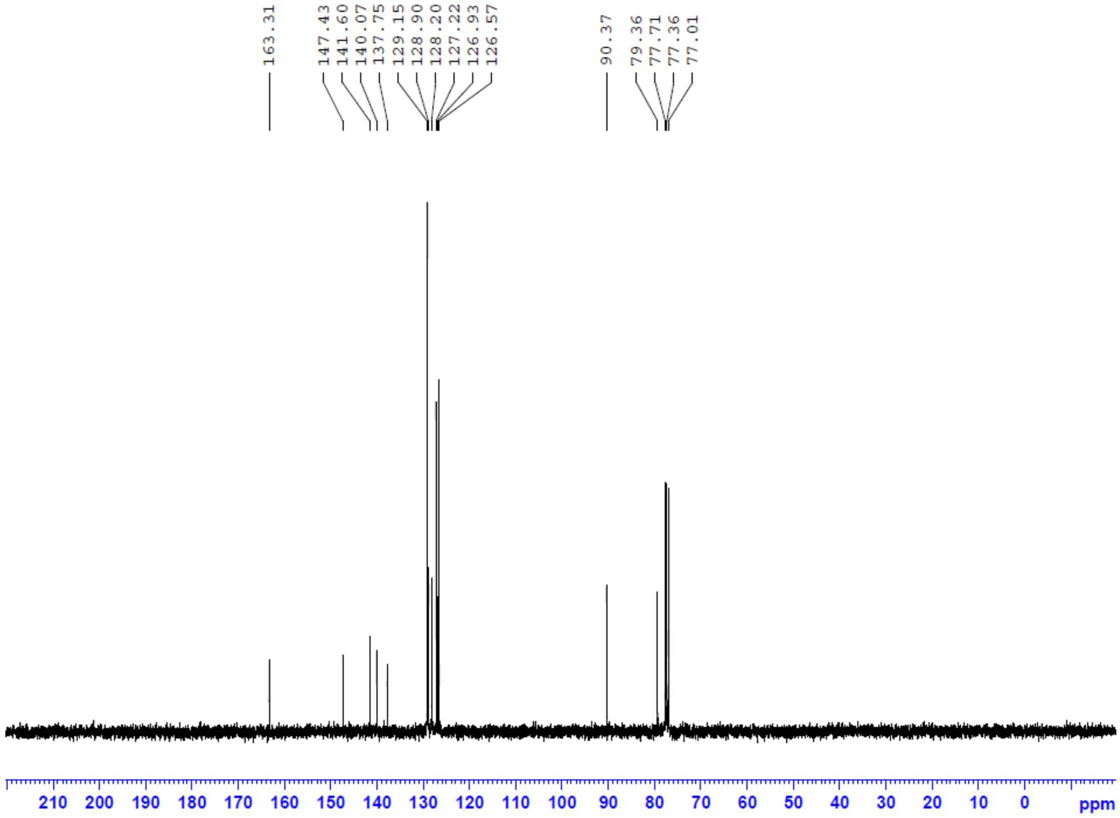

Supplement: Supplementary file 1 [file molecules-24-01141-s001.pdf]
